# Cuproptosis in ccRCC: key player in therapeutic and prognostic targets

**DOI:** 10.3389/fonc.2023.1271864

**Published:** 2023-10-27

**Authors:** Yang Lv, Qiang Li, Lu Yin, Shaohua He, Chao Qin, Zhongwen Lu, Hongqi Chen

**Affiliations:** ^1^ Department of Urology, The Affiliated Jiangsu Shengze Hospital of Nanjing Medical University, Suzhou, China; ^2^ Department of Traditional Chinese Medicine, Hong Kong Baptist University, Hong Kong, Hong Kong SAR, China; ^3^ Department of Urology, The Affiliated Hospital of Nanjing Medical University, Nanjing, China

**Keywords:** clear cell renal cell carcinoma, cuproptosis, tumor immune microenvironment, immunotherapy, prognosis

## Abstract

**Background:**

Classical biomarkers have been used to classify clear cell renal cell carcinoma (ccRCC) patients in a variety of ways, and emerging evidences have indicated that cuproptosis is closely related to mitochondrial metabolism, thereby accelerating the development and progression of ccRCC. Nevertheless, the specific relationship between cuproptosis and the prognosis and treatment of ccRCC remains unclear.

**Methods:**

We comprehensively integrated several ccRCC patient datasets into a large cohort. Following that, we systematically analyzed multi-omics data to demonstrate the differences between two cuproptosis clusters.

**Results:**

We identified two cuproptosis clusters in ccRCC patients. Among the two clusters, cluster 1 patients showed favorable prognosis. We then confirmed the significant differences between the two clusters, including more typical cancer hallmarks were enriched in cluster 2 patients; cluster 2 patients were more susceptible to develop mutations and had a lower level of gistic score and mRNAsi. Importantly, both Tumor Immune Dysfunction and Exclusion analysis and subclass mapping algorithm showed that cuproptosis 1 patients were more susceptible to be responded to immunotherapy. In addition, a prognostic signature was successfully developed and also showed prominent predictive power in response to immunotherapy.

**Conclusion:**

As a result of our findings, we were able to classify ccRCC patients according to cuproptosis in a novel way. By constructing the cuproptosis clusters and developing the signature, patients with ccRCC could have a more accurate prognosis prediction and better immunotherapy options.

## Introduction

Renal cell carcinomas are a group of malignancies originating from renal tubular epithelial cells with different biological characteristics, aggressive behavior, biomarkers and clinical prognosis (1). Clear cell renal cell carcinoma (ccRCC), papillary RCC, and chromophobe RCC are the three dominant subtypes, accounting for more than 90% of all RCC types. Most prominently, ccRCC accounts for approximately 70% of real-world clinical cases of RCC and has highly malignant features (2). There are approximately 400,000 new cases of ccRCC diagnosed each year (3). At the same time, the mechanisms underlying the development of ccRCC are increasingly elucidated, including genetic mutational features, activation of pro-oncogenic pathways, and tumor microenvironment crosstalk (4).

Regulated cell death (RCD) is a biologically sophisticated regulatory mechanism that is widely present in physiological and pathological pathways (5). Disruptions in the normal regulatory program of RCD could subsequently lead to various pathological diseases such as immune disorders, infectious diseases, and malignancies (6–8). Cuproptosis is a newly identified cell death pathway that is closely related to mitochondrial metabolism. Based on the study of Tsvetkov and colleagues, FDX1 is the core gene of the copper death metabolic pathway and has been identified as a gene related to the copper death process together with LIAS, LIPT1, DLD, DLAT, PDHA1, PDHB, MTF1, GLS, and CDKN2A (9). Still today, despite some studies focused on the association between cuproptosis and ccRCC existing, the potential therapeutic role of cuproptosis- related genes in ccRCC remains inadequate.

In this study, not only comprehensive bioinformatics analyses including consensus clustering method, prognosis modeling analysis, genome analysis, immune infiltration analysis, and therapeutic response analysis were performed, *in- vitro* experiments were also conducted to validate our findings. Multiple approaches were used to predict immunotherapeutic efficiency, and the results revealed that patients in cuproptosis 1 group or with low riskScore were more likely to be susceptible to immunotherapy. Consistent with this, *in- vitro* experiments indicated that XXXX identified through our analyses were highly expressed in the ccRCC cells.

## Methods

### Retrieval of the data and correction of the batch effect

After a comprehensive review and synthesis of a wide range of public databases, data of RNA sequences and related clinical information of ccRCC patients were gathered from The Cancer Genome Atlas (TCGA) database (https://tcga-data.nci.nih.gov/tcga/), Gene Expression Omnibus database (https://www.ncbi.nlm.nih/geo/query/), ArrayExpress database (https://www.ebi.ac.uk/arrayexpress/), International Cancer Genome Consortium database (https://dcc.icgc.org/), and Clinical Proteomic Tumor Analysis Consortium database (https://proteomics.cancer.gov/programs/cptac/). In order to construct a validation cohort with enough samples, ccRCC samples from different databases were integrated as a whole dataset using the “ComBat” algorithm based on R software (4.2.0). Then, 539 ccRCC patients in testing cohort and 669 patients in validation cohort were enrolled in the further study. Finally, all the high-throughput sequencing data were transformed into transcripts per million values to make them better match microarray data, and low-abundance genes were filtered to ensure them closer to the signal strength chip data (10, 11).

### Unsupervised consensus clustering and functional analysis

To determine the differentially abundant features of cuproptosis across different ccRCC patients, the “ConsensusClusterPlus” R package was used to separate patients into two clusters based on the expression of 13 cuproptosis-related genes and the survival curve analysis was also conducted between two clusters. Additionally, different clusters were compared in terms of clinical characteristics. To evaluate pathway enrichment, gene set variation analysis (GSVA) was applied to the hallmark gene set through the “gsva” R package. In addition, Gene Ontology (GO) annotation and Kyoto Encyclopedia of Genes and Genomes (KEGG) pathway enrichment analysis were used to identify the pathway and function of differentially expressed genes (DEGs) between different clusters.

### Comparison of genomic characteristics between clusters

To explore the genomic landscape of molecules involved in two clusters, the differential analyses of tumor mutation burden (TMB), copy number variation (CNV), and tumor stemness index (mRNAsi) were subsequently performed. Considering the importance of immune infiltration in tumor microenvironment, stromal, immune, and estimate scores were calculated for each sample based on the “ ESTIMATE” R package. In addition, the representation of 22 immune cells and 29 immune functions were quantified using the CIBERSORT and ssGSEA algorithms. As part of our study, we also evaluated 50 immune checkpoints in terms of their expression across different clusters of patients.

### Analysis of therapeutic sensitivity

Using Tumor Immune Dysfunction and Exclusion (TIDE) and subclass mapping, each sample’s response to immune therapy was predicted. Using the “pRRophetic” R package, the candidate agents with different drug sensitivity between the two cluster samples were identified based on Genomics of Drug Sensitivity in Cancer (GDSC, https://www.cancerrxgene.org), the Cancer Therapeutics Response Portal (CTPR, https://portals.broadinstitude.org/ctrp), and Profiling Relative Inhibition Simultaneously in Mixtures (PRISM) repurposing dataset (https://depmap.org/portal/prism/).

### Construction of prognostic signature based on cuproptosis-related genes

Weighted Gene Co-expression Network Analysis (WGCNA) was used to examine the associations between coexpressed gene modules and clinical traits. We selected genes within the modules with the most significant *P*-values for further analyses. To construct prognostic signature, random forest algorithm was used to identify hub genes. The prognostic value of the signature in both testing data and validation was explored through survival analysis and area under the curve (AUC). In addition, we compared the expression levels of hub genes between normal and tumor tissues.

### Analysis of immune items and study of treatment response

To further determine the role of the signature in ccRCC genesis and treatment, we collected immunotherapy-related signatures from the known literature (12) and hallmark gene signatures from Molecular Signatures Database (http://software.broadinstitute.org/gsea/msigdb) to perform correlation analyses. Furthermore, correlation analyses between genomic characteristics including mRNAsi, EREG-mRNAsi, CNV gain, loss, and riskScore were also conducted. In addition to TIDE analysis, we further included patients administered immune checkpoint inhibitor (ICI) therapy from two independent cohorts (IMvigor210 and GSE78220) to verify the role of the riskScore in predicting different treatment outcomes including complete response (CR), partial response (PR), stable disease, and progressive disease (PD). The riskScore of each patient was calculated using the same formula to assess its relationship with ICI therapy effectiveness.

### Quantitative real-time polymerase chain reaction

Combined with TCGA- expression data and Kaplan–Meier (K-M) survival curve, FUCA1, SLC16A12, CYFIP2, and LIMCH1 were selected to further verify their expression in 10 pairs of ccRCC tissues and corresponding paracancer tissues. All tissues were derived from radical nephrectomy specimens and were pathologically confirmed as ccRCC. Informed consent was obtained from all patients before taking samples, and the study was approved by the ethics committee of the medical institution.

We used RNA Isolation Kit (Vazyme, Nanjing, China) to extract total RNA from ccRCC and adjacent normal tissues. For reverse transcription PCR (RT-PCR), RNA was reverse transcribed using the Reverse Transcription Kit (Vazyme # R333, Nanjing, China). The StepOnePlus™ PCR instrument (Thermo Fisher Scientific, Waltham, MA, U SA) was used for quantitative real-time polymerase chain reaction (qRT-PCR) using SYBR Green Master Kit (Vazyme, Nanjing, China) as fluorescent dye. The primers we used were purchased from GenScript (Nanjing, China). The sequences of the primers are listed here: FUCA1: 5′- GAAGCCAAGTTCGGGGTGTT -3′ (forward) and 5′-GGGTAGTTGTCGCGCATGA-3′ (reverse); SLC16A12: 5′-TCACTCAGGATTACGCACAAAC-3′ (forward) and 5′-TCCCACTTGACAGGATAAATGGT-3′ (reverse); CYFIP2: 5′- CAACGTGGACCTGCTTGAAGA -3′ (forward) and 5′- AGTTTGTGTCAAAGTTAGCCTGG -3′ (reverse); LIMCH1: 5′- CAGACGCCTTCACCAGATGTA -3′ (forward) and 5′- GATGAGGCAAGTCGGATTCAG -3′ (reverse). β-actin: 5′-CCCATCTATGAGGGTTACGC-3′ (forward) and 5′-TTTAATGTCACGCACGATTTC-3′ (reverse). Each qRT-PCR experiment was performed in triplicate, and β-actin was selected to normalize the expression level of target genes.

## Results

### ccRCC patients sort into two clusters according to cuproptosis-related genes

**Figure 1:**
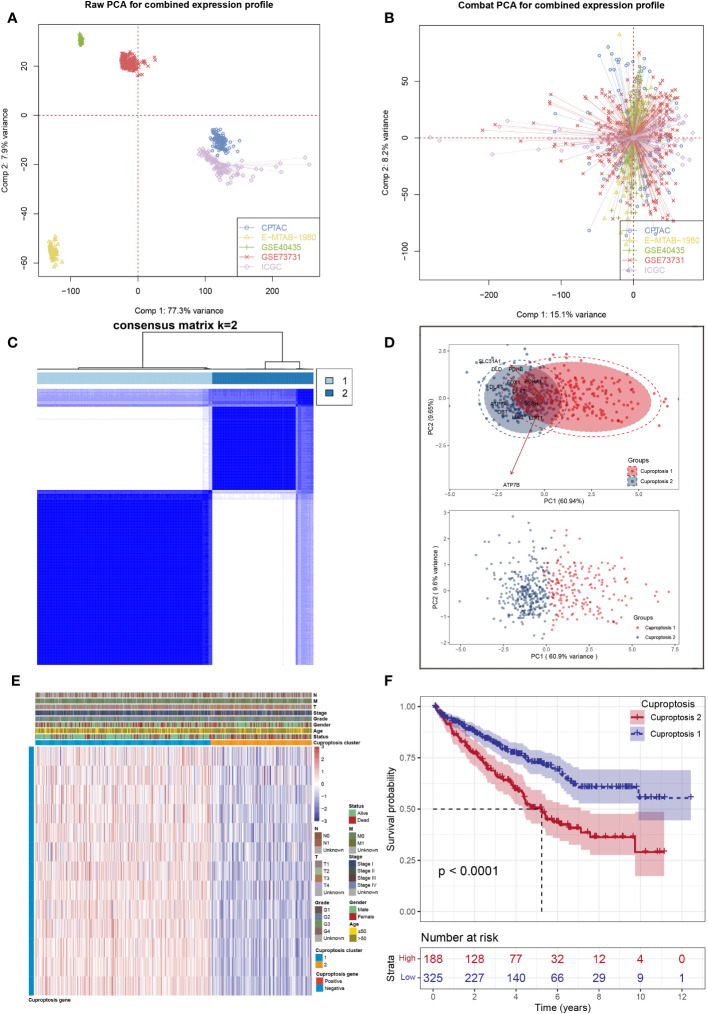
Data processing and clustering. **(A)** Five ccRCC cohorts available have noticeable batch differences; **(B)** reducing the batch difference and the integration of these ccRCC cohorts; **(C)** consensus map of clustering; **(D)** PCA plot for the expression profles of cuproptosis-related genes to distinguish cuproptosis subtypes; **(E)** the heat map based on the expression levels of cuproptosis-related genes between two clusters; **(F)** Kaplan–Meier analysis showed significantly different overall survival time between two clusters.

First, five ccRCC datasets were acquired with complete survival data as the validation cohort, from which a significant batch effect was observed (**Figure 1A**). Then, batch effects were removed to correct biases based on the “ComBat” algorithm (**Figure 1B**). Second, unsupervised clustering analysis was conducted using 13 cuproptosis-related genes to investigate expression patterns of cuproptosis-related genes and divide patients into two clusters (**Figure 1C**). In addition, the principal component analysis (PCA) results showed there was a clear distinction of distribution in the samples between two clusters (**Figure 1D**). In addition, a heat map was created to show the expression of 13 cuproptosis-related genes and different clinical characteristics between patients in two clusters (**Figure 1E**). Finally, a substantial difference was observed between two clusters in terms of overall survival (**Figure 1F**).

### Analyses of clinical characteristics and functional enrichment

**Figure 2:**
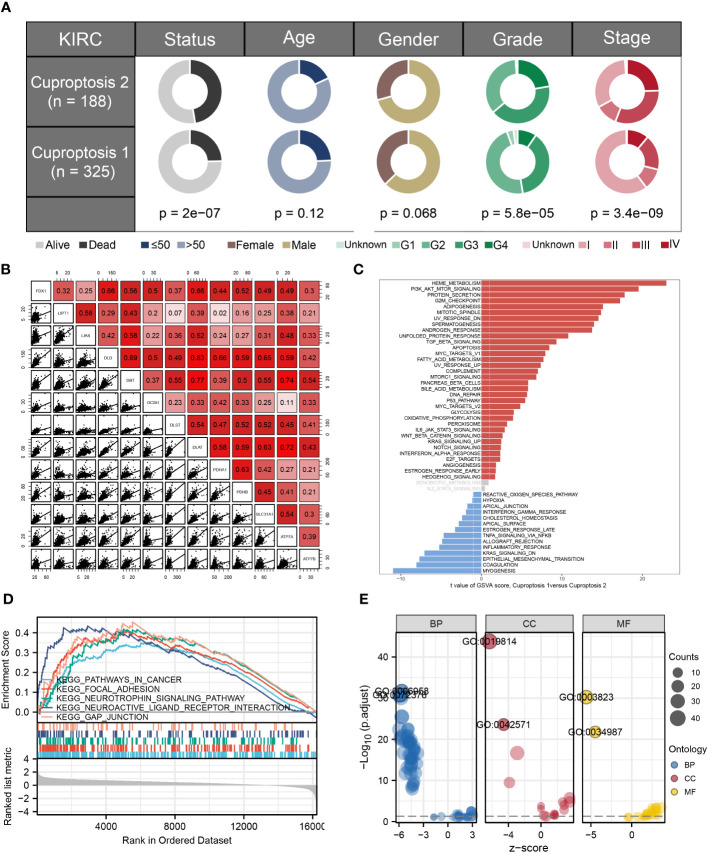
Clinical relevance analysis and functional enrichment analysis. **(A)** Comparisons of different clinicopathological features between two clusters; **(B)** results of correlation analyses between cuproptosis-related genes; **(C)** GSVA analysis based on commonly regulated hallmarks between two clusters; **(D, E)** functional enrichment analyses including KEGG and GO analysis between two clusters.

To identify whether two clusters associated with the clinical characteristics, we compared two clusters’ clinical characteristics and found that survival status, grade, and stage varied between the clusters (**Figure 2A**). **Figure 2B** presented the correlations between all these cuproptosis-related genes. Then, to investigate enriched functions associated with cuproptosis-related genes, the hallmark gene set was used for enrichment analysis based on DEGs between two clusters (**Figure 2C**). In addition, GO and KEGG analysis were also conducted and the results showed the top 5 enriched terms were pathway in cancer, focal adhesion, neurotrophin signaling pathway, neuroactive ligand receptor interaction, and gap junction (**Figures 2D, E**).

### Alterations in the genome related to clusters

**Figure 3:**
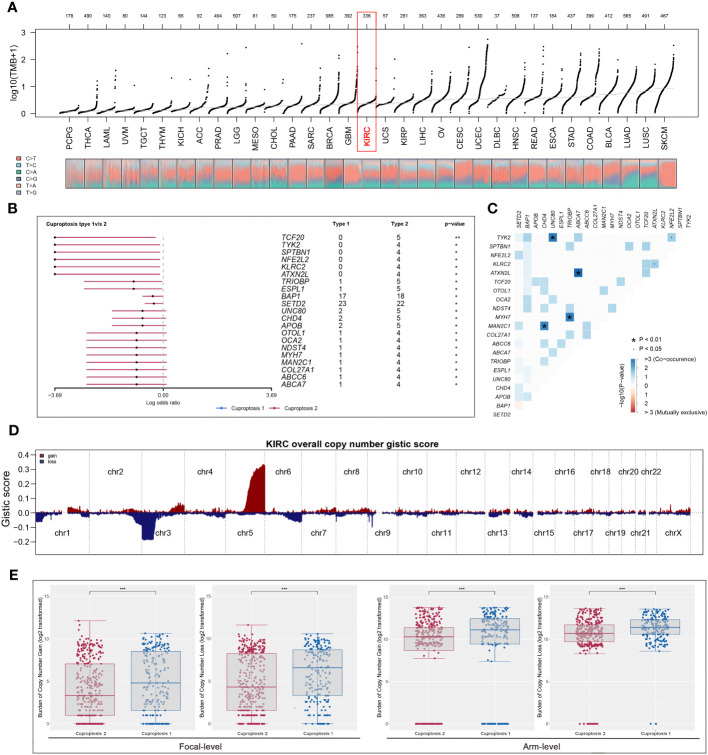
The exploration of differential genomic difference between two clusters. **(A)** The levels of TMB among all tumors based on TCGA dataset; **(B)** cuproptosis 2 patients were more susceptible to develop mutations; **(C)** the relationship between the top 20 differential mutant genes; **(D, E)** cuproptosis 2 cluster patients had significantly higher scores of amplification and deletion mutations. * is equal to P < 0.05; *** is equal to P < 0.001.

Based on TMB analysis of 33 types of cancer, we found that the mutations were relatively low in the ccRCC cohort (**Figure 3A**). Furthermore, the top 20 differential mutant genes were identified, and it was found that all these genes were distributed into cuproptosis 2 patients (**Figure 3B**), then **Figure 3C** showed the co-occurrence and exclusive relationship between these differential mutant genes. There were significant amplifications and deletions in the ccRCC genome that patients in cuproptosis 2 cluster had significantly higher scores of amplification and deletion mutations than those in cuproptosis 1 cluster (**Figures 3D, E**).

### Analysis of immune infiltration and prediction of therapeutic response

**Figure 4:**
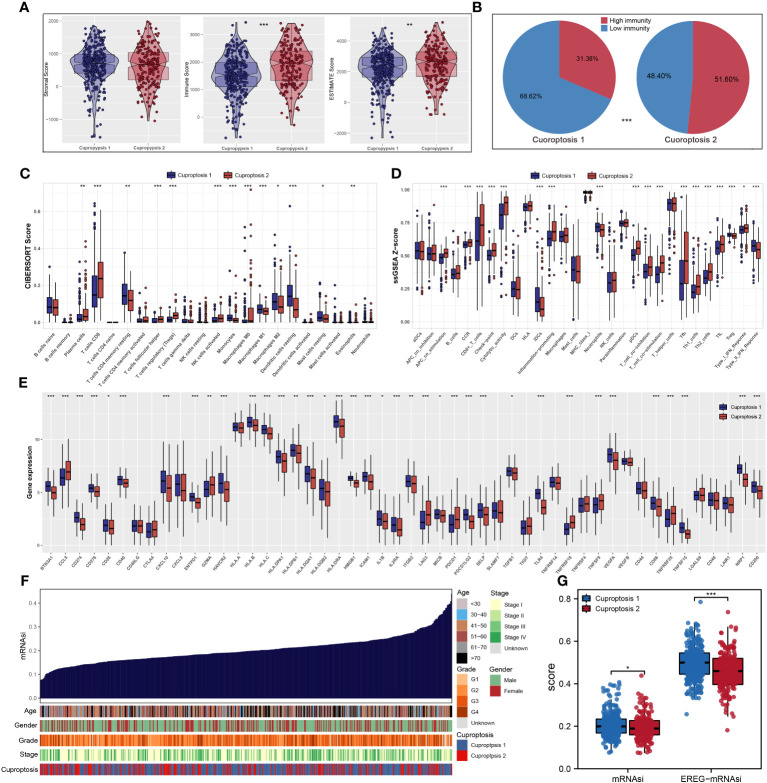
The landscape of immune infiltration and the comparison of mRNAsi score. **(A)** Cuproptosis 2 cluster patients had significantly higher scores of immune and ESTIMATE scores; **(B)** most patients with high immunity were in cuproptosis 2 cluster based on ssGSEA algorithm; **(C, D)** CIBERSORT and ssGSEA algorithms showed that cuproptosis 2 cluster patients had a high level of most immune cells abundance and immune function scores; **(E)** the expression levels of most common immune checkpoints between two clusters; **(F, G)** the quantification of mRNAsi for each patient and cuproptosis 1 cluster patients had a high level of mRNAsi. * is equal to P < 0.05; ** is equal to P < 0.01; *** is equal to P < 0.001.

**Figure 5:**
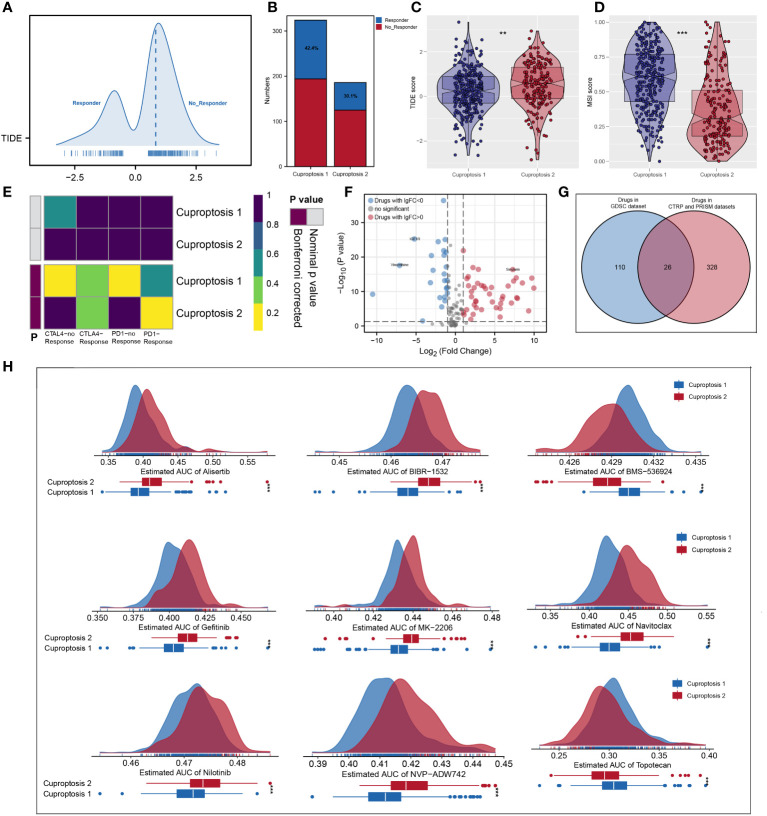
Predictive value in immunotherapy response and mining of appropriate agents. **(A, B)** The quantification of TIDE score for each patient and cuproptosis 1 cluster had more responders; **(C, D)** patients in cuproptosis 1 cluster had a lower TIDE score and a higher MSI score; **(E)** subclass mapping analysis indicated patients in cuproptosis 1 could be more sensitive to the PD-1 inhibitor; **(F, G)** identification of the potential agents based on GDSC dataset, CTRP and PRISM datasets; **(H)** the top 9 drugs with significant differences in sensitivity AUC. ** is equal to P < 0.01; *** is equal to P < 0.001.

According to ESTIMATE analysis, patients in cuproptosis 1 cluster had lower immune and ESTIMATE scores than those in cuproptosis 2 cluster (**Figure 4A**). Then, based on ssGSEA algorithm, the immune score of each patient was quantified and we found that most high- immunity patients were in cuproptosis 2 cluster (**Figure 4B**). For each cluster, the CIBERSORT and ssGSEA algorithms were used to assess immune cell abundance and immune function scores, from which we could see that patients in cuproptosis 1 cluster had a high level of most immune cells, while patients in cuproptosis 2 cluster had a higher level of most immune functions and CD8+ T cell (**Figures 4C, D**). Next, we examined immune checkpoint expression levels between two clusters, and we found that cuproptosis 1 cluster patients were more highly expressed (**Figure 4E**). For each sample, **Figure 4F** showed the mRNAsi distribution and clinical characteristics including age, gender, grade, stage, and cluster, and the lower mRNAsi and EREG-mRNAsi were observed in the cuproptosis 2 cluster from differential expression analysis (**Figure 4G**). According to TIDE analysis, patients with lower TIDE scores are more likely to respond to immunotherapy (13). Only 30.1% of patients in cuproptosis 2 cluster responded to immunotherapy, compared to 42.4% of patients in cuproptosis 1 cluster (**Figures 5A, B**). Microsatellite instability (MSI) is a phenomenon of hypermutation that presents at genomic microsatellites and is caused by the insertion or deletion of a repeat unit (14). We found that cuproptosis 1 cluster patients had a lower TIDE score and a higher MSI score than cuproptosis 2 cluster patients (**Figures 5C, D**), which was in harmony with submap analysis indicating that anti–PD-1 treatments were more effective in cuproptosis 1 patients (**Figure 5E**). Then we identified the potential drugs for ccRCC patients using GDSC dataset, CTRP and PRISM datasets, respectively (**Figures 5F, G**). Moreover, the top 9 drugs with significant differences in sensitivity AUC were shown in **Figure 5H**.

### Construction of a gene signature based on cuproptosis-related genes

**Figure 6:**
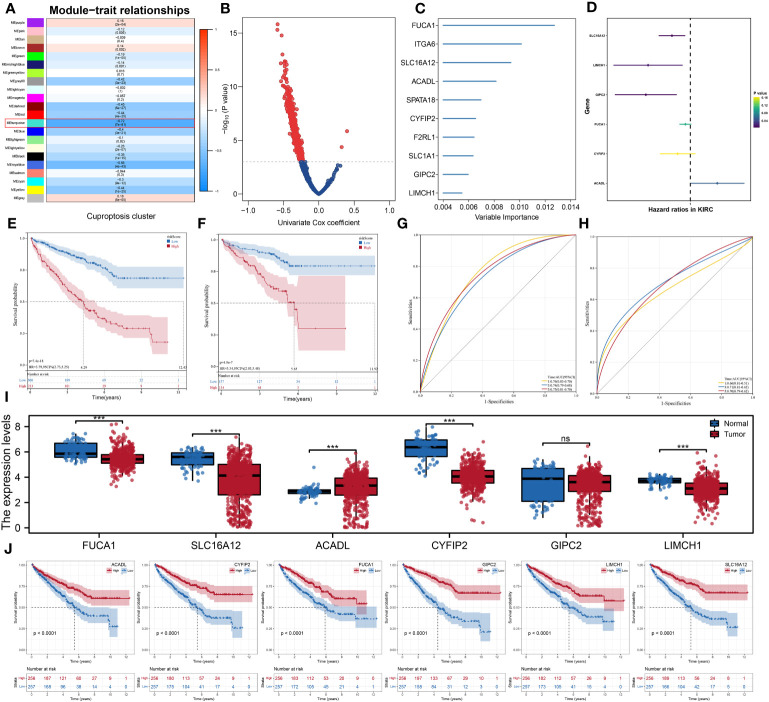
Development of the prognostic signature using WGCNA algorithm and Cox regression analysis. **(A)** The gene co-expression networks of patients based on the WGCNA algorithm; **(B)** Volcano plot of the results of univariate cox regression analysis; **(C)** the 10 hub genes were identified using the random forest algorithm; **(D)** the results of multivariate cox regression analysis; **(E, F)** Kaplan–Meier analysis showed significantly different OS between two risk groups in both testing and validation cohorts; **(G, H)** ROC analysis showed good predictive power of the signature in both testing and validation cohorts; **(I)** the comparison of the expression levels of the six signature genes between two risk groups; **(J)** the Kaplan–Meier survival curves of the six signature genes. *** is equal to P < 0.001. ns, not significant.

The gene co-expression networks of the ccRCC patients were developed through the WGCNA algorithm and the genes in the turquoise module were identified with the most correlation with cuproptosis (**Figure 6A**). Using univariate Cox regression analysis, we further selected prognostic genes from the module and then used the random forest algorithm to identify hub genes to establish the prognostic signature (**Figures 6B, C**). Finally, the signature consisting of six genes, namely, SLC16A12, LIMCH1, GIPC2, FUCA1, CYFIP2, and ACADL, was constructed using multivariate Cox regression analysis (**Figure 6D**). According to the optimal cutoff value calculated through “maxstat” algorithm for each sample, we classified patients into high- and low-risk groups, and there was a significant difference in overall survival time between two groups in both testing and validation cohorts (**Figures 6E, F**). In addition, the receiver operating characteristic (ROC) curves also showed promising results in both testing and validation cohorts (**Figures 6G, H**). Then, we found that the expression of ACADL was increased in ccRCC tissues, while SLC16A12, LIMCH1, GIPC2, FUCA1, and CYFIP2 were decreased (**Figure 6I**). **Figure 6J** showed the results of K-M analyses between these signature genes.

### Characterization of the immune landscape and immunotherapy response

**Figure 7:**
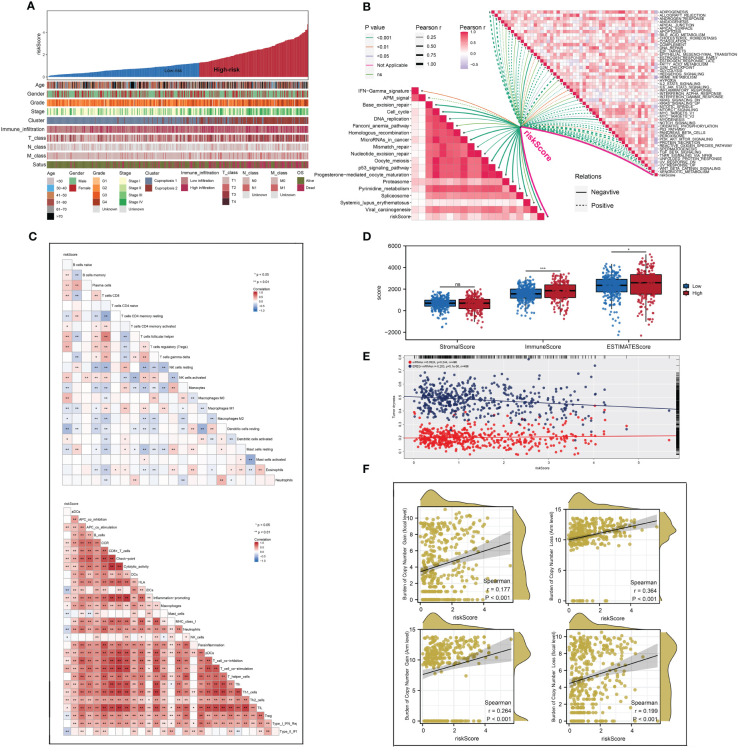
The difference of genomic feature and immune infiltration between two risk groups. **(A)** The distribution of riskScore of each patient with their clinical features; **(B)** the correlations between riskScore and the scores of immunotherapy-related signatures and hallmark gene signatures; **(C, D)** the landscape of immune infiltration in each patient and patients in high-risk group had a higher level of immune infiltration; **(E, F)** patients in low- risk group had a higher tumor dryness and lower levels of amplification and deletion mutations. * is equal to P < 0.05; ** is equal to P < 0.01; *** is equal to P < 0.001. ns, not significant.

**Figure 8:**
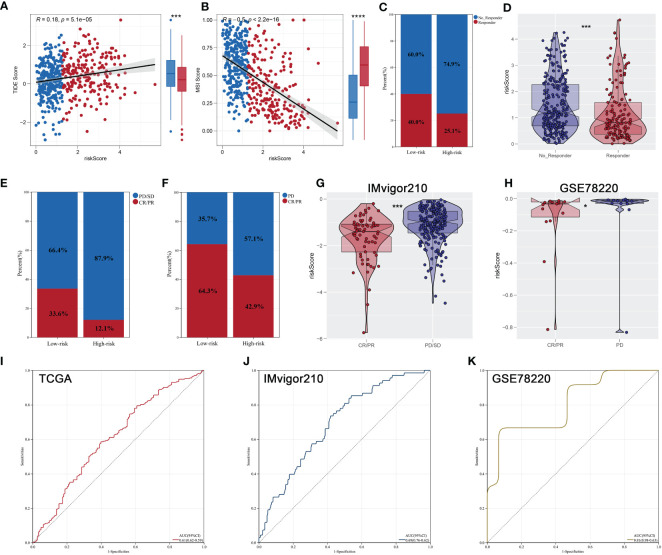
Immunotherapeutic response prediction. **(A, B)** Patients with low riskScore had a lower level of TIDE and higher level of MSI score; **(C)** patients in low- risk group have a higher percentage of responders; **(D)** the responders had a lower riskScore; **(E, F)** the proportion of patients with response to immunotherapy in IMvigor210 and GSE78220 cohorts; **(G, H)** comparison of riskScore between two risk groups of different response to immunotherapy in IMvigor210 and GSE78220 cohorts; **(I–K)** ROC curves indicated superior predictive accuracy of immunotherapeutic response in TCGA, IMvigor210, and GSE78220 cohorts. * is equal to P < 0.05; *** is equal to P < 0.001.

Each patient’s riskScore and different clinical features were displayed in **Figure 7A**. Furthermore, 18 immunotherapy-related signatures from the known literature and hallmark gene signatures were quantified to conduct correlation analysis with riskScore, from which we found that riskScore had a negative association with most immunotherapy-related signatures (**Figure 7B**). In addition, the relationship between riskScore and immune infiltration signatures was also explored (**Figures 7C, D**). In addition, a higher tumor dryness including mRNAsi and EREG-mRNAsi and lower levels of amplification and deletion mutations at both focal and arm levels were observed in low-risk group (**Figures 7E, F**). Considering the immunotherapy holds great promise in the treatment of ccRCC, a particular focus was placed on the potential role of riskScore in predicting the response to immunotherapy. TIDE analysis was performed on patients in TCGA cohort, and the result showed that patients with low riskScore had a lower level of TIDE and higher level of MSI score (**Figures 8A, B**). In addition, responders in low-risk group made up 40.0%, while responders in high-risk group made up 25.1% (**Figure 8C**), and the riskScore of responders were significantly lower than those of non-responders (**Figure 8D**). To strengthen the credibility of our findings, two independent cohorts of patients receiving ICI therapy including IMvigor210 and GSE78220 were selected to validate the predictive power of the signature. Our findings revealed that most patients with the outcome of CR or PR were in the low-risk group and exhibited a significantly lower level of riskScore (**Figures 8E–H**). Finally, ROC analyses between these three cohorts also demonstrated satisfactory accuracy, indicating that the riskScore was strongly associated with the response to immunotherapy (**Figures 8I–K**).

### Quantitative PCR analysis

**Figure 9:**
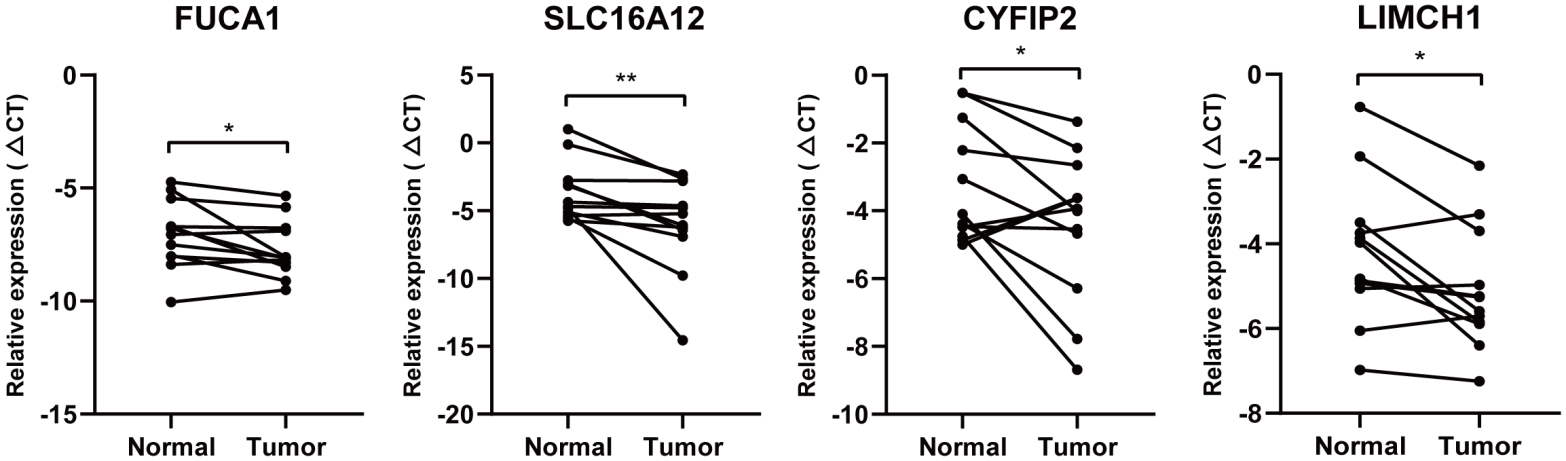
Quantitative real-time PCR. FUCA1, SLC16A12, CYFIP2, and LIMCH1 mRNA level in 10 paired clinical ccRCC samples. * is equal to P < 0.05; ** is equal to P < 0.01.

Given that FUCA1, SLC16A12, CYFIP2, and LIMCH1 are lowly expressed in ccRCC tissues and that low expression of the four genes has a worse clinical prognosis, we further validated the expression of FUCA1, SLC16A12, CYFIP2, and LIMCH1 mRNA levels in 10 pairs of paired ccRCC and matched adjacent tissues. qPCR results demonstrated that the expression of FUCA1, SLC16A12, CYFIP2, and LIMCH1 was downregulated in ccRCC tissues compared with adjacent normal tissues (**Figure 9**).

## Discussion

As the malignant tumor with the highest mortality rate in the urinary system, ccRCC has brought a heavy burden to the world health system. Although surgical resection of the tumor offers promising treatment prospects, disease progression still occurs in approximately 30% of patients (15). It is urgent to find a new mechanism for the occurrence and development of ccRCC.

RCD is a form of regulatory death that is different from accidental cell death. The regulatory mechanisms of apoptosis, entosis, necroptosis, pyroptosis, and ferroptosis have been found in solid tumors (8). Meanwhile, a growing number of RCD-related genes have been shown to be involved in the development of ccRCC (16–19). Metal ions are important cofactors widely present in biological sessions. Since the elucidation of the ferroptosis regulatory program in 2012 (20), there have been numerous studies confirming that ferroptosis affects the malignant progression of various cancers, including ccRCC (18, 19, 21). Noteworthy, copper ion is an essential trace metal element in the human body and plays a pivotal role in body composition, biotransformation, and signaling chain (22). Recently, cuproptosis has come to the attention of researchers, which has been identified as a new type of cell death. Ten genes with FDX1 as the core gene were identified to be intimately associated with the cuproptosis process (9).

In this study, two cuproptosis clusters were identified for subgrouping patients with ccRCC. GSVA analysis showed that some pivotal pathways including HEME metabolism, PIK3/AKT/mTor pathways, secreted protein, and G2M checkpoint were activated in cuproptosis 2 cluster patients, partially explaining the dismal prognosis. In addition, KEGG and GO analyses also showed that functional enrichment pathways varied considerably across the two clusters. More importantly, genome analyses indicated that almost all differential mutated genes occurred in cuproptosis 2 cluster patients, and the level of mRNAsi was lower in cuproptosis 2 cluster patients. Then, we performed a highly comprehensive immune analysis between two clusters patients, from which a high expression level of most immune infiltration terms including CD8+ T cells, B cells, macrophages, and TIL were found in cuproptosis 2 cluster patients. All these findings provide us evidence that cuproptosis 2 cluster patients may have a better immunotherapy response. Therefore, in order to interrogate and confirm the therapeutic role of the expression level of cuproptosis, a variety of methods to predict therapeutic response were conducted deeply. In combination with TIDE analysis, patients with low TIDE scores who are at cuproptosis 1 cluster are more promising in responding to ICB. Other than this, to complete the validation of the immunotherapy response prediction, subclass mapping analysis also indicated that PD1 could be more effective in cuproptosis 1 cluster when treated. It was still noteworthy that the discrepancy of sensitivity AUC values of drugs in different datasets including GDSC, CTRP and PRISM datasets was also observed between the two cuproptosis clusters. All these findings strongly showed that it would be possible to differentiate between tumor immune microenvironment patterns and to identify patients who might benefit from ICI treatment using the established cuproptosis clusters.

Considering the multifaceted heterogeneities cuproptosis subtypes displayed, our group considered that such heterogeneities and the creation of individual, integrative assessments could be quantified by creating a prognostic signature. In line with expectations, a close correlation was also observed between the constructed signature and clinicopathological features, typical cancer hallmarks and genomic features. Among these signature genes, SLC16A12 has been previously reported to have excellent effectiveness and clinical application value in ccRCC (23). There is an unfavorable association between LIMCH1 protein expression and distant metastasis-free survival in breast cancer (24). In addition, as well as being a component of exosomes, the GIPC2 paralog plays a key role in WNT signaling pathways associated with tumor progression and was shown to be robustly stimulating the adhesion, invasion, and migration of prostate cancer (25). As a key member of the cytoplasmic FMR1-interacting protein family, CYFIP2 may be a novel prognostic gene that is related to immune infiltration in ccRCC (26). Moreover, as ACADL expression was restored in hepatocellular carcinoma cells, the Hippo/YAP signaling pathway was suppressed, resulting in growth suppression and cell cycle arrest (27). Taken together, there is no doubt that these cuproptosis-related signature genes are involved in the occurrence and the development of various cancers. However, a more in-depth association between the processes of cuproptosis and these genes and how these genes affect the occurrence and development of ccRCC need to be explored in the future.

Until now, according to classical biomarkers, ccRCC patients have been classified in so many ways. Even more, few cuproptosis-associated gene signatures have been developed and offered some help in the diagnosis, treatment, and prognosis of ccRCC patients (28, 29). Compared with the previous classifications of ccRCC patients, the advantage of our cuproptosis subtyping was its ability to show multi-dimensional heterogeneity. Moreover, most of all, differently from other approaches to distinguishing ccRCC patients, our study was more comprehensive than the previous study, including clinicopathological features, commonly regulated hallmarks, genomic characteristics, and immunotherapeutic responses, especially. In spite of this, there are still a few inadequacies in this study. First, although we integrated all publicly available ccRCC patient data, more clinical data from different countries and regions are needed. Second, since there are fewer immunotherapy cohorts available reported that Only IMvigor210 and GSE78220 cohorts were able to assess our signature’s predictive value for ICI therapy. Finally, additional experiments are needed to validate our findings.

## Conclusion

To summarize, after a comprehensive integration of several available ccRCC patient datasets, ccRCC patients were divided into two cuproptosis clusters with distinct prognosis, clinicopathological features, commonly regulated hallmarks, genomic characteristics, and immunotherapeutic responses. In addition, a prognostic signature was then successfully developed. It may make it easier for ccRCC patients to predict their prognosis and find better immunotherapy options based on our findings.

## Data availability statement

The original contributions presented in the study are included in the article/**Supplementary Material**. Further inquiries can be directed to the corresponding authors.

## Author contributions

YL: Investigation, Writing – original draft. QL: Software, Writing – original draft. LY: Investigation, Writing – original draft. SH: Software, Writing – original draft. CQ: Investigation, Writing – review & editing. ZL: Software, Writing – original draft. HC: Conceptualization, Writing – review & editing.
