# Supplementary material for: Cuproptosis in ccRCC: key player in therapeutic and prognostic targets
Source: Front Oncol. 2023 Oct 27;13:1271864. doi: 10.3389/fonc.2023.1271864 (PMC10642186; doi:10.3389/fonc.2023.1271864)
Supplement: Supplementary file 1 [file DataSheet_1.zip › Step1/ConsensusCluster/consensus.pdf]

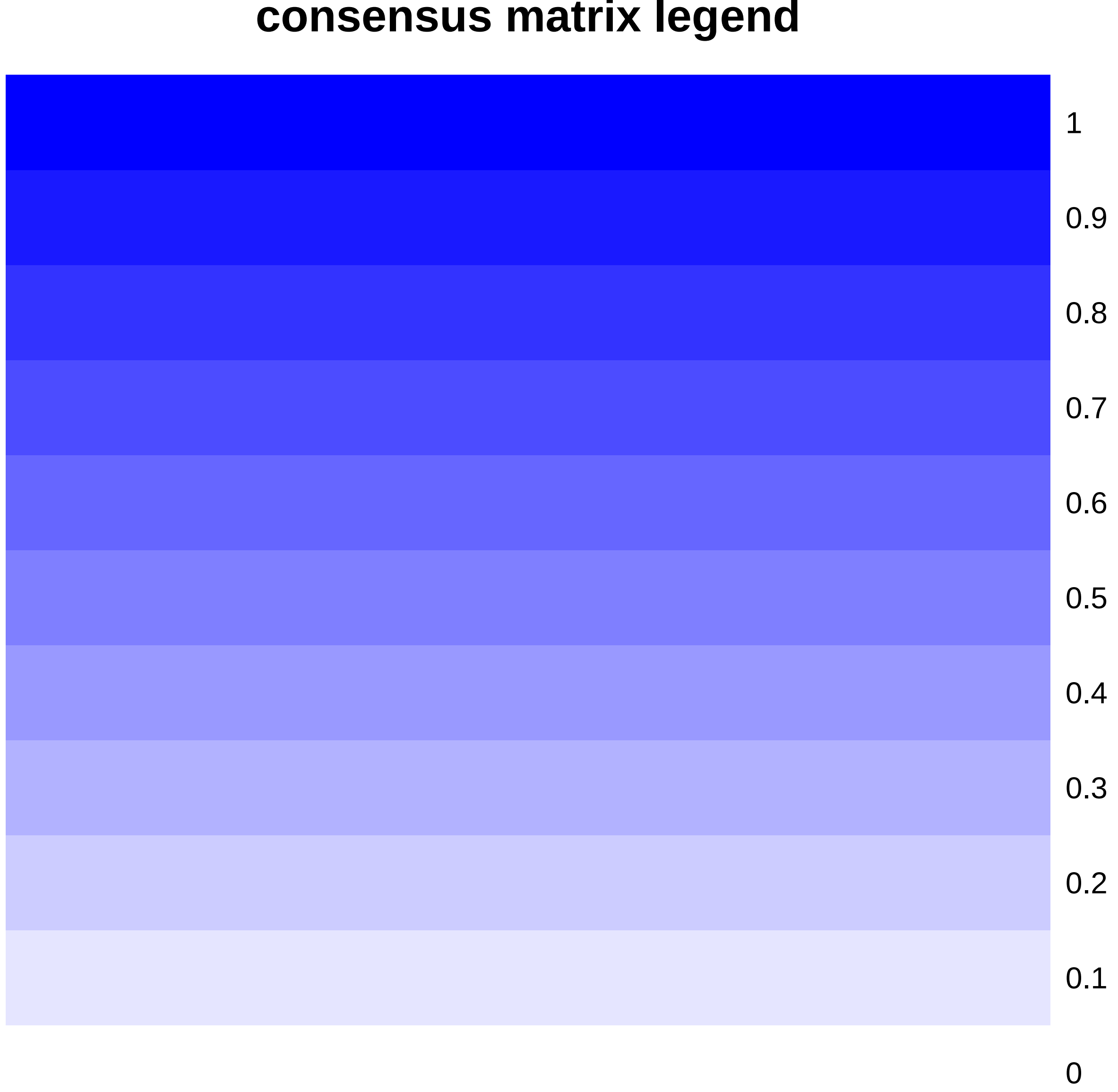
consensus matrix legend
1
0.9
0.8
0.7
0.6
0.5
0.4
0.3
0.2
0.1
0

consensus matrix k=2

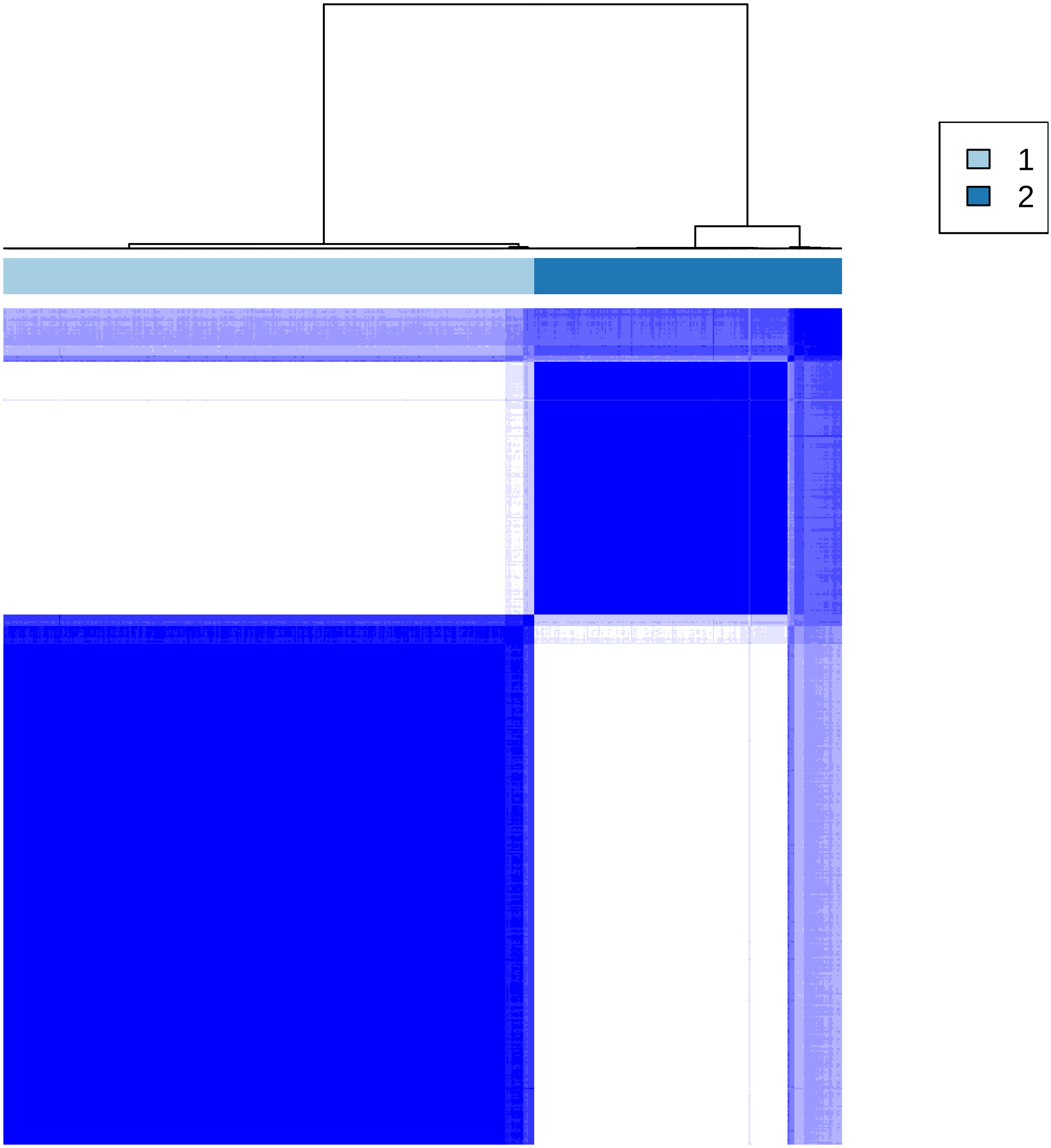

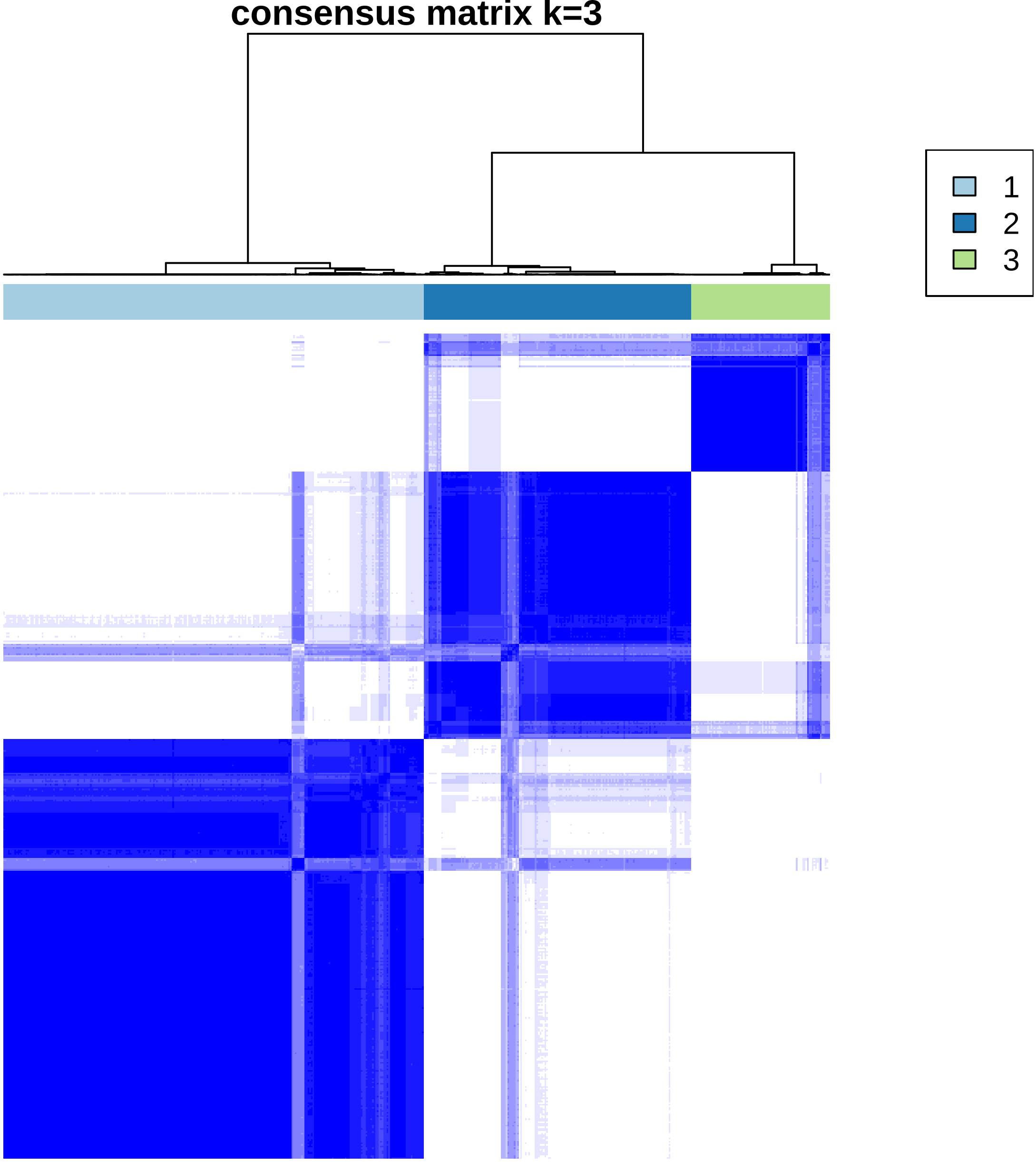
consensus matrix k=3
1
2
3

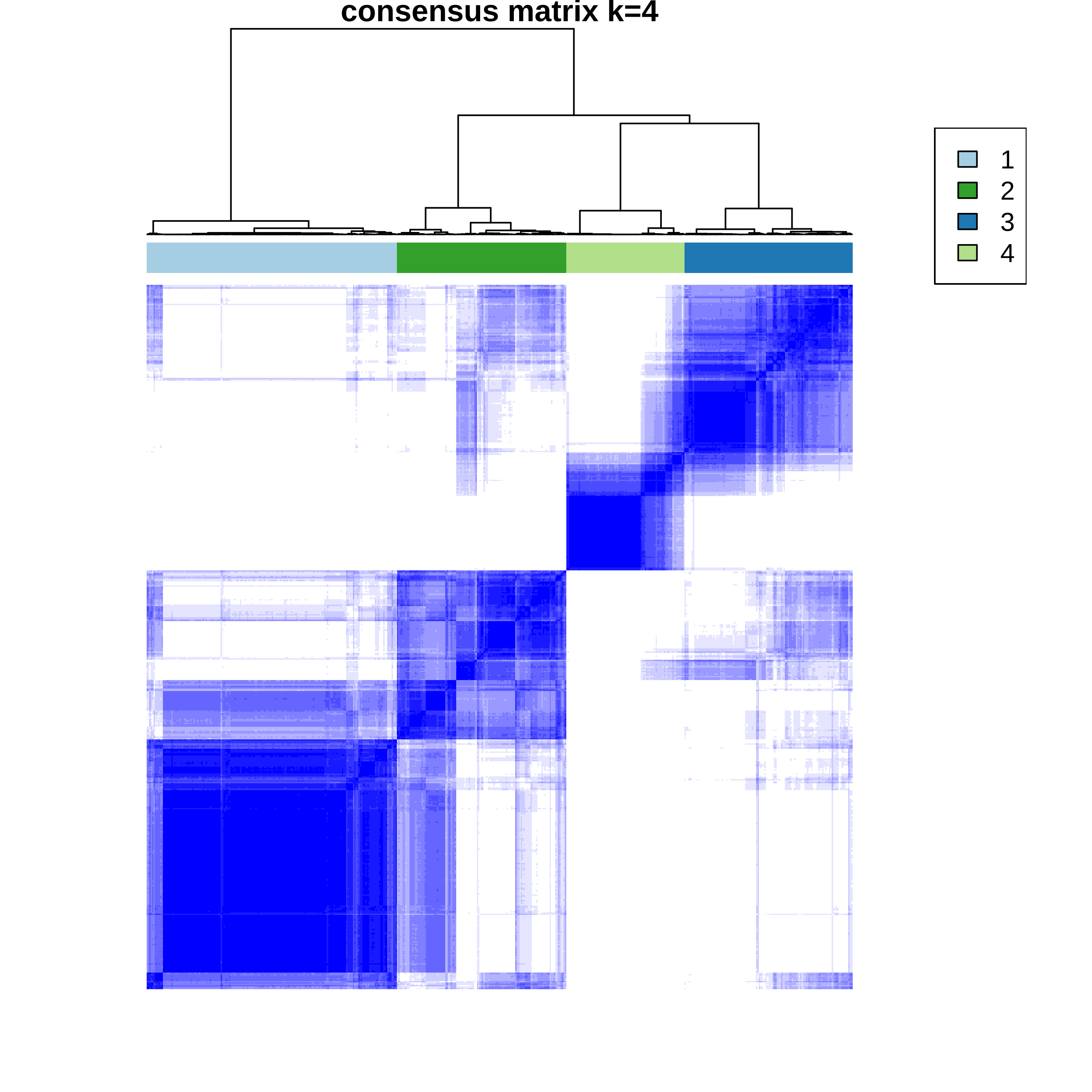
consensus matrix k=4
1
2
3
4

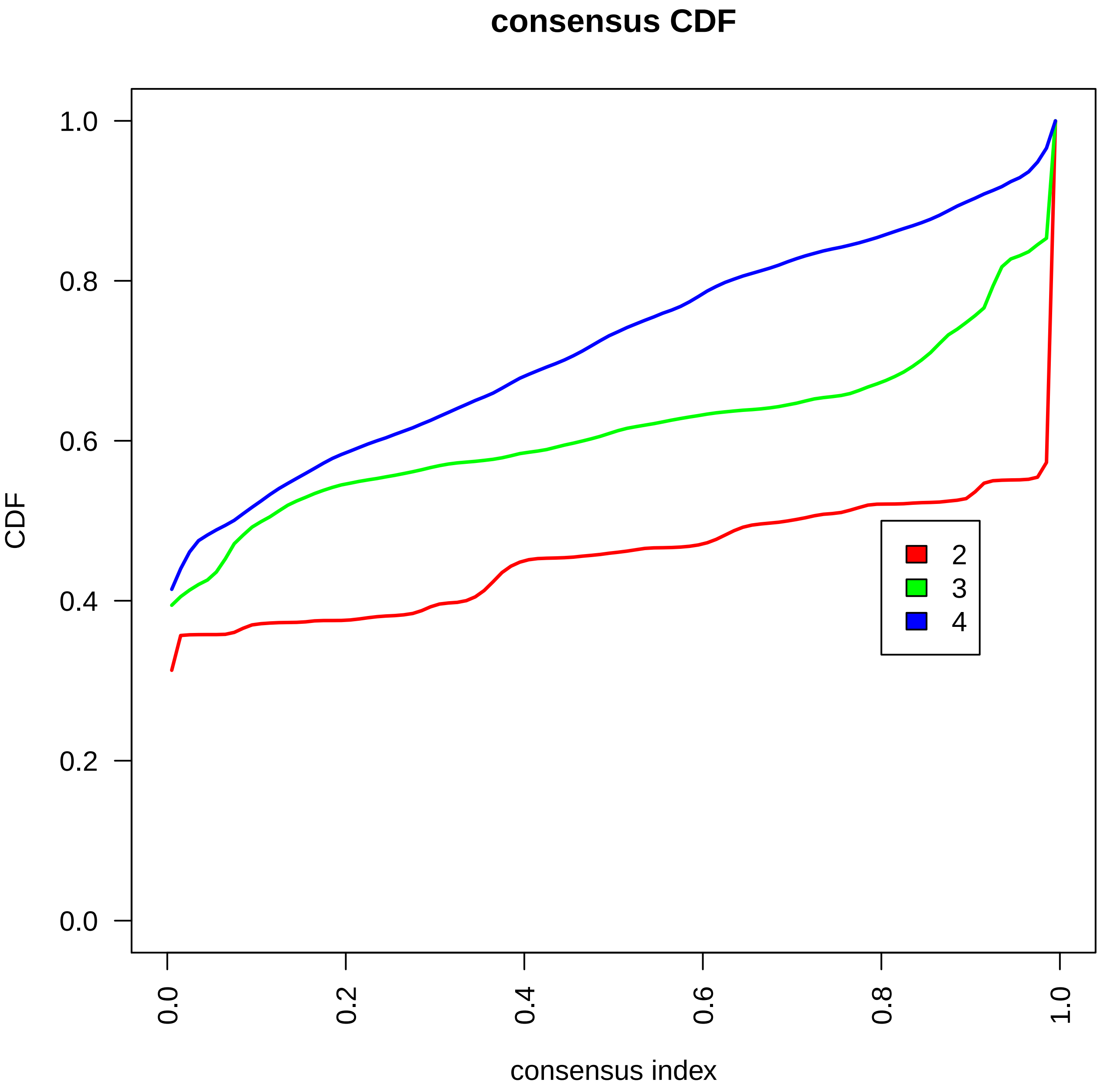

consensus CDF
CDF
consensus index
1.0
0.8
0.6
0.4
0.2
0.0
0.0
0.2
0.4
0.6
0.8
1.0
2
3
4

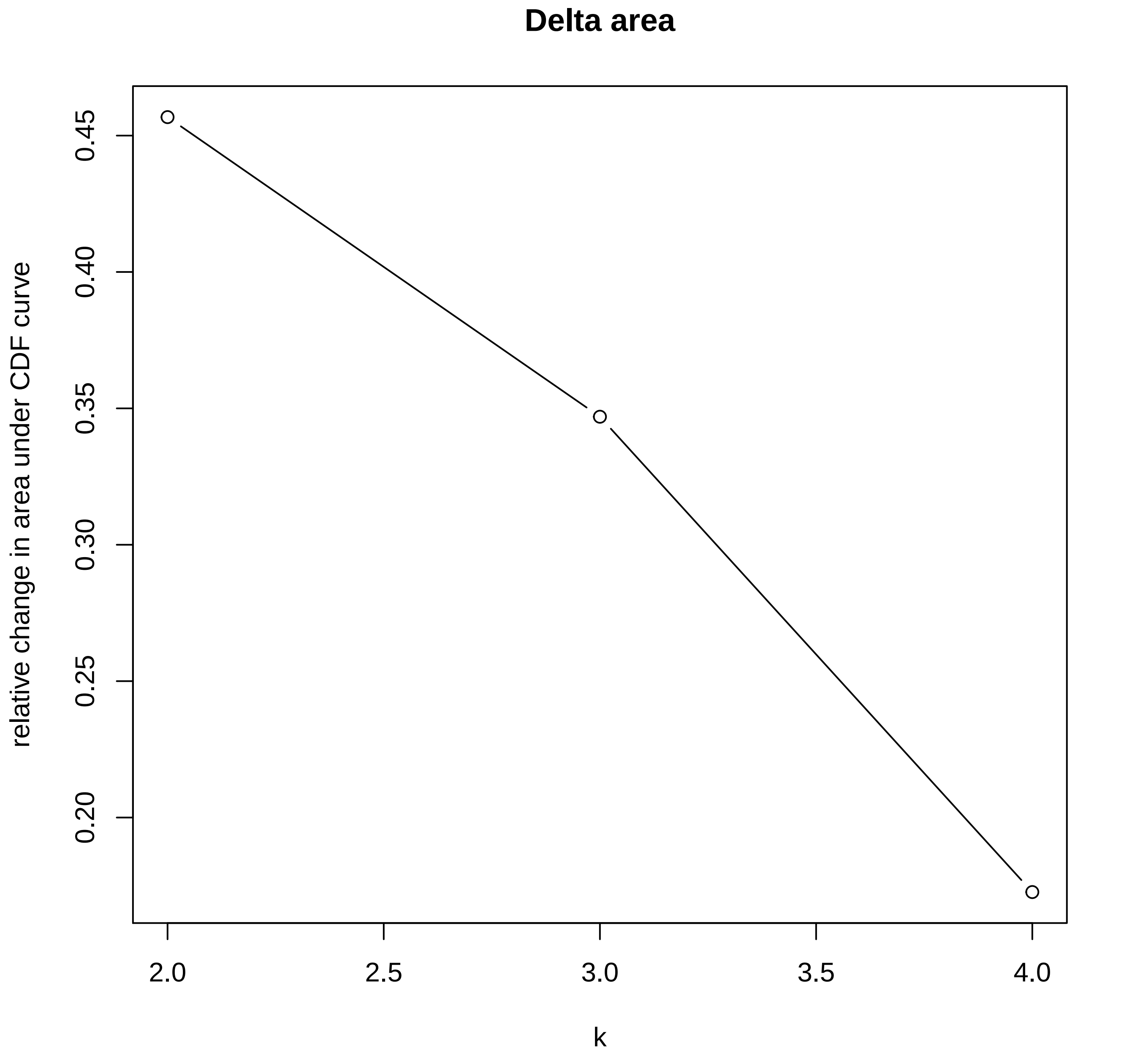

Delta area
relative change in area under CDF curve
0.20
0.25
0.30
0.35
0.40
0.45
2.0
2.5
3.0
3.5
4.0
k

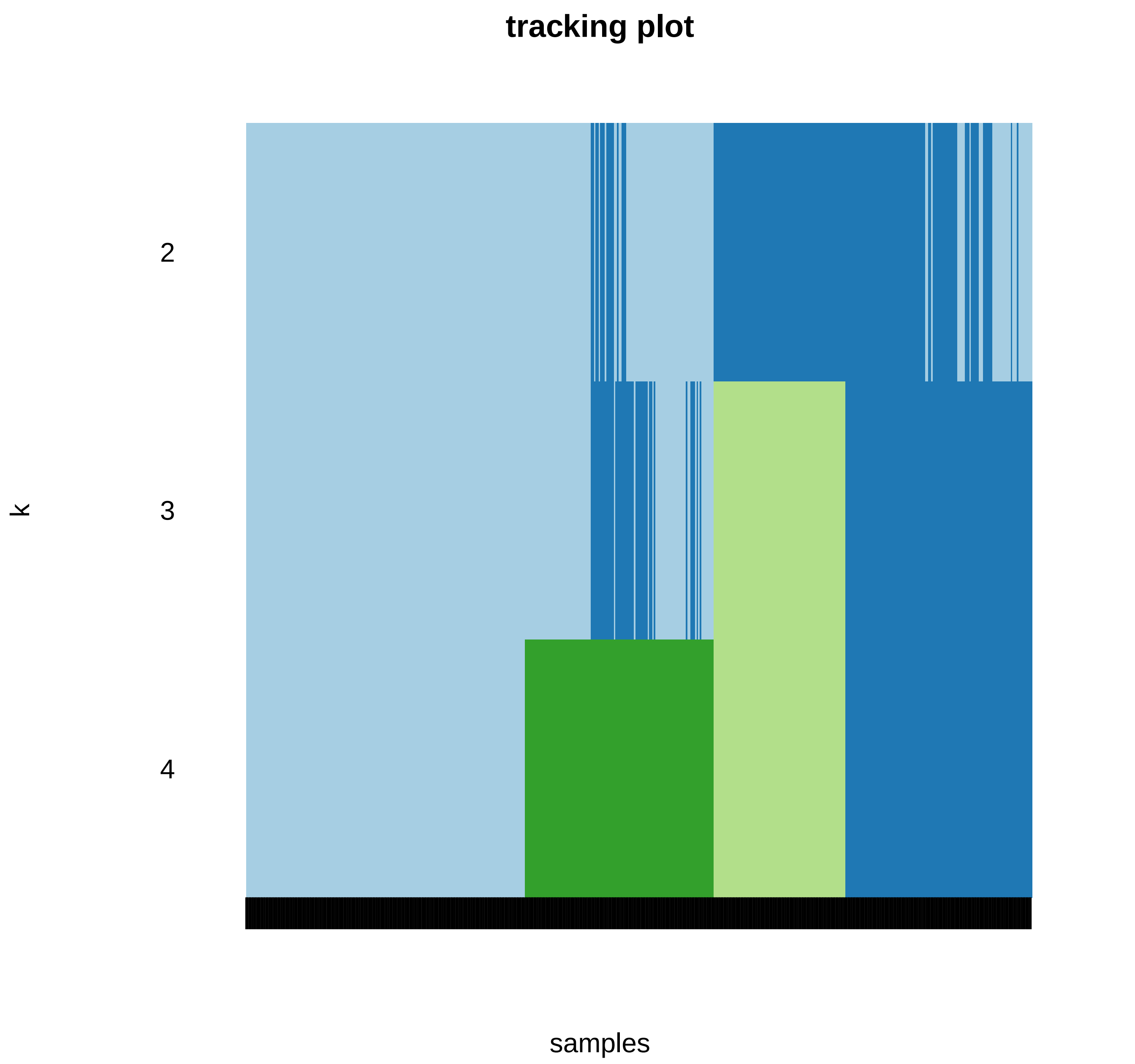
tracking plot
k
2
3
4
samples
