# Supplementary material for: Cuproptosis in ccRCC: key player in therapeutic and prognostic targets
Source: Front Oncol. 2023 Oct 27;13:1271864. doi: 10.3389/fonc.2023.1271864 (PMC10642186; doi:10.3389/fonc.2023.1271864)
Supplement: Supplementary file 1 [file DataSheet_1.zip › Step1/PCA/PCA/FigureYa38PCA.html]

FigureYa38 PCA


# FigureYa38 PCA

#### *小丫画图出品*

#### *9/16/2018*

微信ID: epigenomics E-mail: epigenomics@rainbow-genome.com

本代码作者：徐洲更https://www.jianshu.com/u/9ea40b5f607a

小丫编辑校验

### 需求描述

用TCGA数据画主成分分析(pricinple components analysis，PCA)的图。

例如：画个小圈圈里的这个图：

出自http://www.haematologica.org/content/102/7/e245

出自http://clincancerres.aacrjournals.org/content/23/8/2105.figures-only

### 应用场景

通常我们拿到的数据集，例如转录组的表达量矩阵都有成千上万个基因，这类高维度数据很难直接进行可视化展示。

为了能够在二维平面上展示，就需要对其进行降维，而PCA分析是目前最常用的降维工具。

然后你就能在图上一眼看出哪些样品之间更像，哪些样品属于/不属于同一组。不仅适用于测序数据。

场景一：多个分组，每组三次生物学重复。做PCA，看到某个样品远离了组内其他样品，它有什么问题？

场景二：多个分组，分三批送去测序。做PCA，看不出分组的规律，却看到同一批次的样品间距离更近，批次效应？

### 输入数据

两个输入文件:

- 表达量矩阵：easy\_input\_expr.csv，第一列是样品名，后续几列是各个基因的表达量，或者其他属性
- 样品分组：easy\_input\_meta.csv，第一列是样品名，第二列是分组

此处输入数据为3种癌症BRCA、OV、LUSC里，5个基因“GATA3”, “PTEN”, “XBP1”,“ESR1”, “MUC1”的表达量。

数据来源：用R包`RTCGA.mRNA`提取TCGA数据。也可参考FigureYa18、21（临床信息）、FigureYa22、23、34（表达矩阵）里的数据下载方法。

```
expr_df <- read.csv(file='easy_input_expr.csv',row.names = 1, 
                    header = TRUE, sep=",", stringsAsFactors = FALSE)
meta_df <- read.csv(file='easy_input_meta.csv', row.names = 1,
                    header = TRUE, sep=",",stringsAsFactors = FALSE)
#查看前3个基因在前4个sample中的表达矩阵
expr_df[1:3,1:4]
```

```
##                                GATA3      PTEN     XBP1    ESR1
## TCGA-A1-A0SD-01A-11R-A115-07 2.87050 1.3613571 2.983333 3.08425
## TCGA-A1-A0SE-01A-11R-A084-07 2.16625 0.4283571 2.550833 2.38600
## TCGA-A1-A0SH-01A-11R-A084-07 1.32350 1.3056429 3.020417 0.79125
```

```
#查看样本信息前3行
head(meta_df, n=3)
```

```
##                                  group
## TCGA-A1-A0SD-01A-11R-A115-07 BRCA.mRNA
## TCGA-A1-A0SE-01A-11R-A084-07 BRCA.mRNA
## TCGA-A1-A0SH-01A-11R-A084-07 BRCA.mRNA
```

### 做PCA

```
#用`prcomp`进行PCA分析
pca.results <- prcomp(expr_df, center = TRUE, scale. = FALSE)

#定义足够多的颜色，用于展示分组
mycol <- c("#223D6C","#D20A13","#088247","#FFD121","#11AA4D","#58CDD9","#7A142C","#5D90BA","#431A3D","#91612D","#6E568C","#E0367A","#D8D155","#64495D","#7CC767")
```

### 开始画图

这里提供两种方法：

- 方法一：自动画图。用现成的R包全自动画图，必要时只需调整参数。
- 方法二：提取PCA分析结果，手动画图。

#### 方法一：自动画图

用到做PCA的R包`ggord`，优点是能用背景色展示置信区间，缺点是没有画圈功能。

Y叔对ggord进行了加强，放在R包`yyplot`里，详情看这篇：https://mp.weixin.qq.com/s/iA0IuyRx\_Ojk639sxenmvg。yyplot包里提供的`geom_ord_ellipse`能画出多个圆圈，用来展示不同的置信区间，并且可以反复叠加，想画几个圈就画几个圈。

##### 经典版

```
#install.packages("devtools")
#library(devtools)
#devtools::install_github("GuangchuangYu/yyplot")
#devtools::install_github('fawda123/ggord')
library(ggplot2)
library(plyr)
library(ggord)
#library(yyplot)

#有可能你的网络在安装yyplot时遇到困难，我把geom_ord_ellipse函数单独下载，通过本地进行加载。
#调用yyplot包里的geom_ord_ellipse函数
source('./geom_ord_ellipse.R') #该文件位于当前文件夹

#用ggord画基本PCA图
ggord(pca.results, grp_in = meta_df$group, repel=TRUE,
      ellipse = FALSE, #不显示置信区间背景色
      size = 2, #样本的点大小
      alpha=0.5, #设置点为半透明，出现叠加的效果
      #如果用自定义的颜色，就运行下面这行
      cols = mycol[1:length(unique(meta_df$group))],
      arrow = NULL,txt = NULL) + #不画箭头和箭头上的文字
  theme(panel.grid =element_blank()) + #去除网格线
  
  #用yyplot添加置信区间圆圈
  geom_ord_ellipse(ellipse_pro = .95, #设置置信区间
                   size=1.5, #线的粗细
                   lty=1 ) #实线
```

```
#保存到pdf文件
ggsave("PCA_classic.pdf", width = 6, height = 6)
```

##### 箭头版

```
#用ggord画基本PCA图和置信区间背景色
ggord(pca.results, grp_in = meta_df$group, repel=TRUE,
      alpha = 0.6,#点和置信区间背景设为半透明，以凸显箭头和文字
      #或者单独修改置信区间背景的透明度
      #alpha_el = 0.3,
      ellipse_pro = 0.95,#置信区间
      size = 2,
      #如果想用默认的颜色，就在下面这行前面加个#
      #cols = mycol[1:length(unique(meta_df$group))],
      arrow=0.2, #箭头的头的大小
      vec_ext = 5,#箭头尾巴长短
      veccol="brown",#箭头颜色
      txt=3) + #箭头指向的基因名的字体大小
  theme(panel.grid =element_blank()) + 
  
  #用yyplot继续添加虚线的置信区间
  geom_ord_ellipse(ellipse_pro = .95, #先画个.95的圆圈
                   color='darkgrey', #圈圈的颜色
                   size=0.5, 
                   lty=2 ) + #画成虚线，可以用1-6的数字设置为其他线型
  geom_ord_ellipse(ellipse_pro = .98, #再画个.98的圆圈
                   #color='grey', #把这行注释掉，就是跟点一样的颜色
                   size=0.5, lty=2 )
```

```
#保存到pdf文件
ggsave("PCA_arrow.pdf", width = 6, height = 6)
```

#### 方法二：提取PCA分析结果，手动画图

##### 从PCA结果到画图所需的输入数据整理

用`prcomp`进行PCA分析后，获取降维后每个样本对应的主成分值和每个主成分的解释的变异。

```
#install.packages("ggplot2")
#install.packages("dplyr")
#install.packages("plyr")
library(ggplot2)
library(dplyr)
```

```
## Warning: package 'dplyr' was built under R version 3.5.1
```

```
library(plyr)

pca.rotation <- pca.results$rotation
pca.rotation
```

```
##               PC1         PC2          PC3         PC4         PC5
## GATA3 -0.73161568  0.50418097 -0.007208812  0.45556797 -0.05427619
## PTEN  -0.04655647 -0.04996858 -0.039088666 -0.13771625 -0.98734085
## XBP1  -0.40639073  0.18995246  0.267550892 -0.84471515  0.11677959
## ESR1  -0.53389836 -0.75320303 -0.375073077 -0.02012422  0.08095021
## MUC1  -0.11122097 -0.37404272  0.886654846  0.24401513 -0.04496377
```

```
pca.pv <- summary(pca.results)$importance[2,]
pca.pv
```

```
##     PC1     PC2     PC3     PC4     PC5 
## 0.66986 0.20273 0.06785 0.03502 0.02454
```

调整数据结构，用于作图

提取前两个主成分构建数据框，并增加分组信息列

```
low_dim_df <- as.data.frame(pca.results$x[,c(1,2)])
low_dim_df$group <- meta_df$group
#查看前3行
low_dim_df[1:3,]
```

```
##                                    PC1      PC2     group
## TCGA-A1-A0SD-01A-11R-A115-07 -5.394822 1.551709 BRCA.mRNA
## TCGA-A1-A0SE-01A-11R-A084-07 -4.446423 1.152850 BRCA.mRNA
## TCGA-A1-A0SH-01A-11R-A084-07 -3.199532 2.010018 BRCA.mRNA
```

下面以PCA分析得到的“low\_dim\_df”作为输入，画图

##### 画置信区间圆圈的函数

先运行函数`add_ellipase`，用来增加置信区间椭圆线。

通过调整参数，以达到你想要的效果，参数含义为：

- p: ggplot2返回的对象
- x,y: 主成分的列名
- group: 分组列
- ellipse\_pro: 置信区间，默认0.95
- linetype可选类型: blank, solid, dashed, dotted, dotdash, longdash, twodash
- colour：颜色
- lwd：线的粗细

```
add_ellipase <- function(p, x="PC1", y="PC2", group="group",
                         ellipase_pro = 0.95,
                         linetype="dashed",
                         colour = "black",
                         lwd = 2,...){
  obs <- p$data[,c(x, y, group)]
  colnames(obs) <- c("x", "y", "group")
  ellipse_pro <- ellipase_pro
  theta <- c(seq(-pi, pi, length = 50), seq(pi, -pi, length = 50))
  circle <- cbind(cos(theta), sin(theta))
  ell <- ddply(obs, 'group', function(x) {
    if(nrow(x) <= 2) {
      return(NULL)
    }
    sigma <- var(cbind(x$x, x$y))
    mu <- c(mean(x$x), mean(x$y))
    ed <- sqrt(qchisq(ellipse_pro, df = 2))
    data.frame(sweep(circle %*% chol(sigma) * ed, 2, mu, FUN = '+'))
    })
  names(ell)[2:3] <- c('x', 'y')
  
  ell <- ddply(ell, .(group) , function(x) x[chull(x$x, x$y), ])
  p <- p + geom_polygon(data = ell, aes(x=x,y=y,group = group), 
                   colour = colour,
                   alpha = 1,fill = NA,
                   linetype=linetype,
                   lwd =lwd)
  return(p)
}
```

##### 开始画图

```
#计算坐标轴标签
pc1.pv <- paste0(round(pca.pv['PC1'],digits = 3) * 100, "%")
pc2.pv <- paste0(round(pca.pv['PC2'],digits = 3) * 100, "%")

#画出各个样本在二维空间的点
p <- ggplot(low_dim_df) + 
  geom_point(aes(x=PC1, y=PC2, color=group), size=2, #点的大小
             shape=20,#点的形状
             alpha=0.5) +#设置点为半透明，出现叠加的效果
  #如果使用默认的颜色，就在下面这行前面加个#
  scale_color_manual(values = mycol[1:length(unique(meta_df$group))]) +
  #还能调整整体的颜色亮度
  #scale_colour_hue(l=45) + 
  theme_bw() + #去除背景色
  theme(panel.grid =element_blank()) + #去除网格线
  
  #添加标签，同样可以加到方法一的同一位置
  annotate("text",x=-8.5,y=-1.2,label = "BRCA",color = mycol[1]) +
  annotate("text",x=5,y=5.6,label = "LUCA",color = mycol[2]) +
  annotate("text",x=5,y=-2.5,label = "OV",color = mycol[3]) +
  
  #图例
  guides(color=guide_legend(title = NULL)) +
  theme(legend.background = element_blank(), #移除整体边框
        #图例的左上角置于绘图区域的左上角
        legend.position = c(0,1),legend.justification = c(0,1),
        legend.text = element_text(size=12)) + #字体大小

  #调整坐标轴标签
  xlab(paste0("PC1 ( ", pc1.pv," variance )")) + 
  ylab(paste0("PC2 ( ", pc2.pv," variance )")) 
p
```

```
#画圈圈
p1 <- add_ellipase(p,ellipase_pro = 0.95,colour = "dimgrey",linetype=1,lwd=1)
p1
```

```
#保存为PDF文件
ggsave('PCA_DIY1.pdf',width = 5.5,height = 4)

#也可以多次叠加，画两个圆圈
p2 <- add_ellipase(p,ellipase_pro = 0.95,colour = "dimgrey",linetype=2,lwd=0.5)
p2 <- add_ellipase(p2,ellipase_pro = 0.98,colour = "dimgrey",linetype=2,lwd=0.5)
p2
```

```
#保存为PDF文件
ggsave('PCA_DIY2.pdf',width = 5.5,height = 4)
```

### 附录

之前提到三个癌症的全称

- BRCA: Breast invasive carcinoma
- OV: Ovarian serous cystadenocarcinoma
- LUSC: Lung squamous cell carcinoma

```
sessionInfo()
```

```
## R version 3.5.0 (2018-04-23)
## Platform: x86_64-apple-darwin15.6.0 (64-bit)
## Running under: macOS High Sierra 10.13.6
## 
## Matrix products: default
## BLAS: /Library/Frameworks/R.framework/Versions/3.5/Resources/lib/libRblas.0.dylib
## LAPACK: /Library/Frameworks/R.framework/Versions/3.5/Resources/lib/libRlapack.dylib
## 
## locale:
## [1] en_US.UTF-8/en_US.UTF-8/en_US.UTF-8/C/en_US.UTF-8/en_US.UTF-8
## 
## attached base packages:
## [1] stats     graphics  grDevices utils     datasets  methods   base     
## 
## other attached packages:
## [1] dplyr_0.7.6   ggord_1.1.1   plyr_1.8.4    ggplot2_3.0.0
## 
## loaded via a namespace (and not attached):
##  [1] Rcpp_0.12.18     bindr_0.1.1      knitr_1.20       magrittr_1.5    
##  [5] tidyselect_0.2.4 munsell_0.5.0    colorspace_1.3-2 R6_2.2.2        
##  [9] rlang_0.2.1      stringr_1.3.1    tools_3.5.0      grid_3.5.0      
## [13] gtable_0.2.0     withr_2.1.2      htmltools_0.3.6  yaml_2.1.19     
## [17] lazyeval_0.2.1   rprojroot_1.3-2  digest_0.6.15    assertthat_0.2.0
## [21] tibble_1.4.2     bindrcpp_0.2.2   purrr_0.2.5      ggrepel_0.8.0   
## [25] glue_1.2.0       evaluate_0.10.1  rmarkdown_1.10   labeling_0.3    
## [29] stringi_1.2.3    compiler_3.5.0   pillar_1.2.3     scales_1.0.0    
## [33] backports_1.1.2  pkgconfig_2.0.1
```
