# Supplementary figures and images for: Cuproptosis in ccRCC: key player in therapeutic and prognostic targets (part 1 of 2)
Source: Front Oncol. 2023 Oct 27;13:1271864. doi: 10.3389/fonc.2023.1271864 (PMC10642186; doi:10.3389/fonc.2023.1271864)

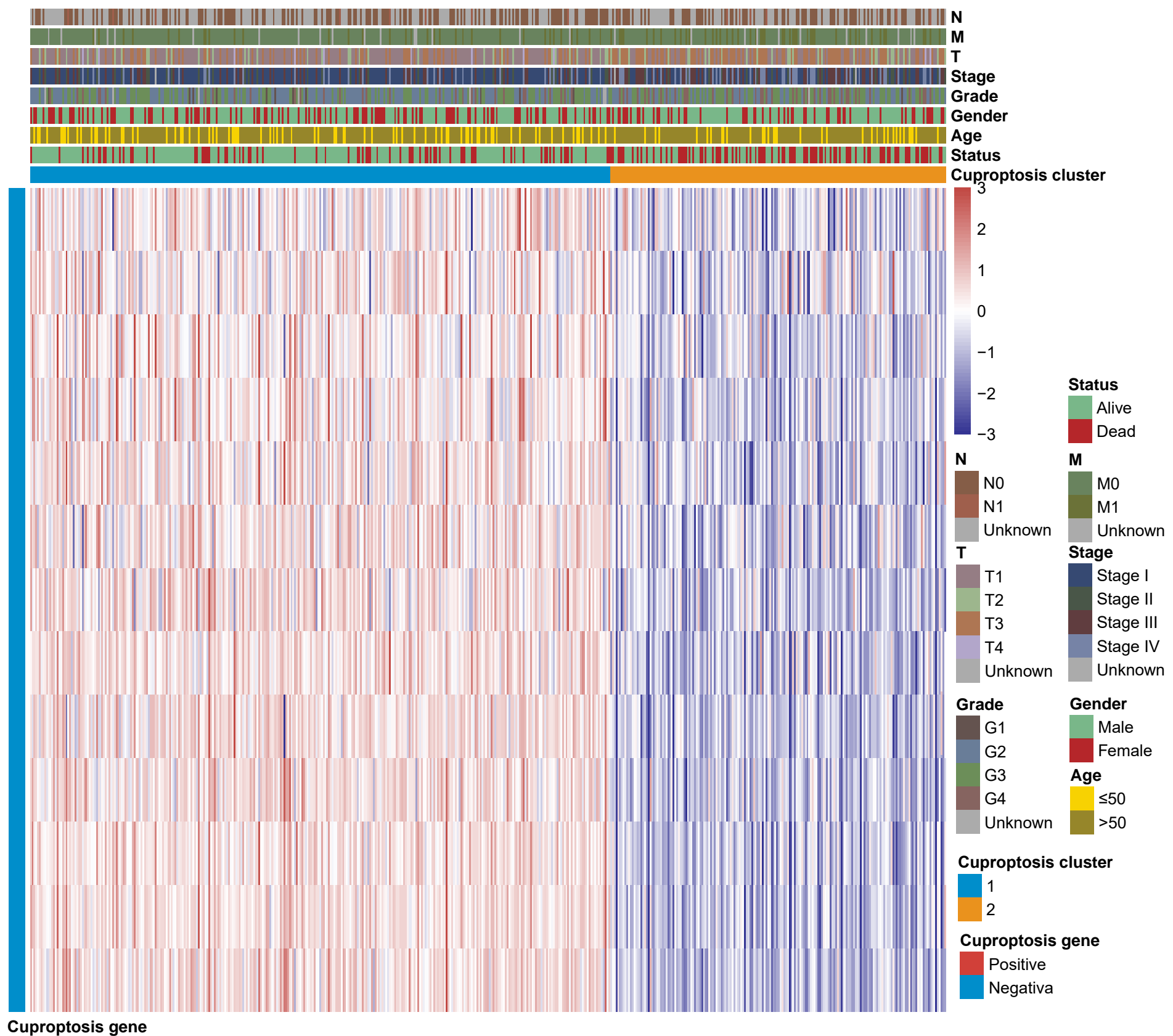

Supplement: Supplementary file 1 [file DataSheet_1.zip › Step1/1.pdf]

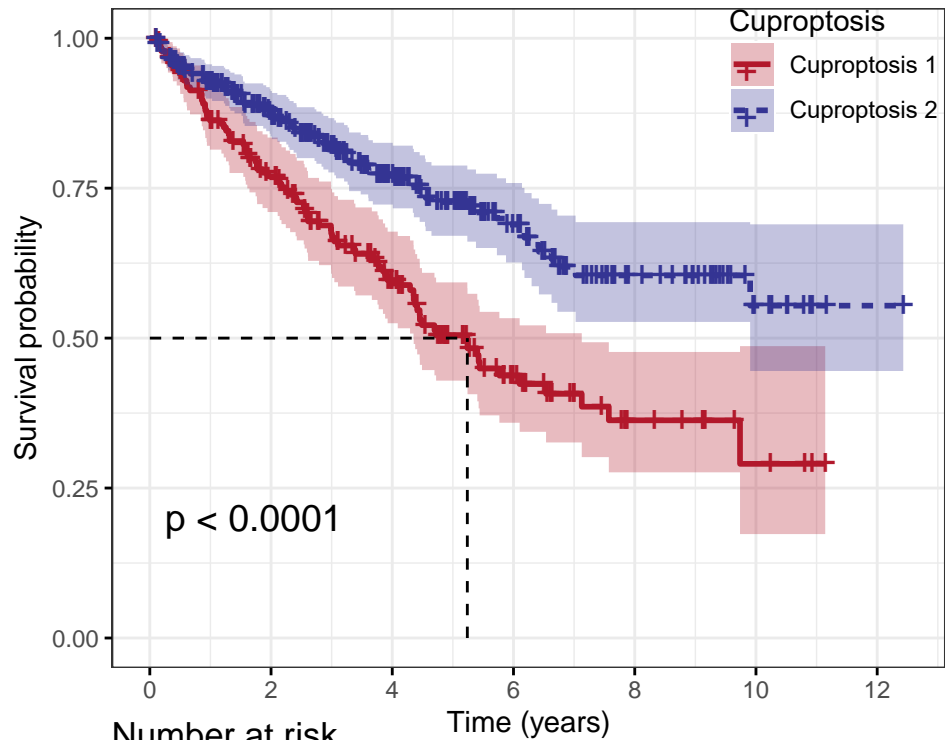

Number at risk

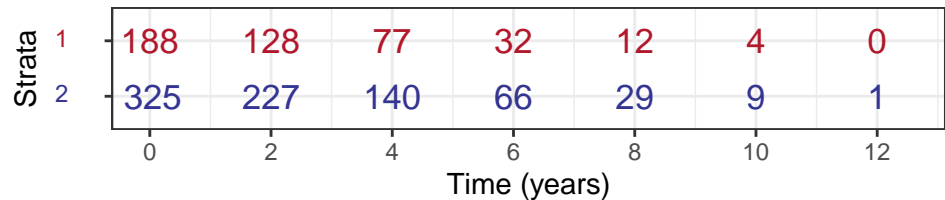

Supplement: Supplementary file 1 [file DataSheet_1.zip › Step1/1_survival2.pdf]

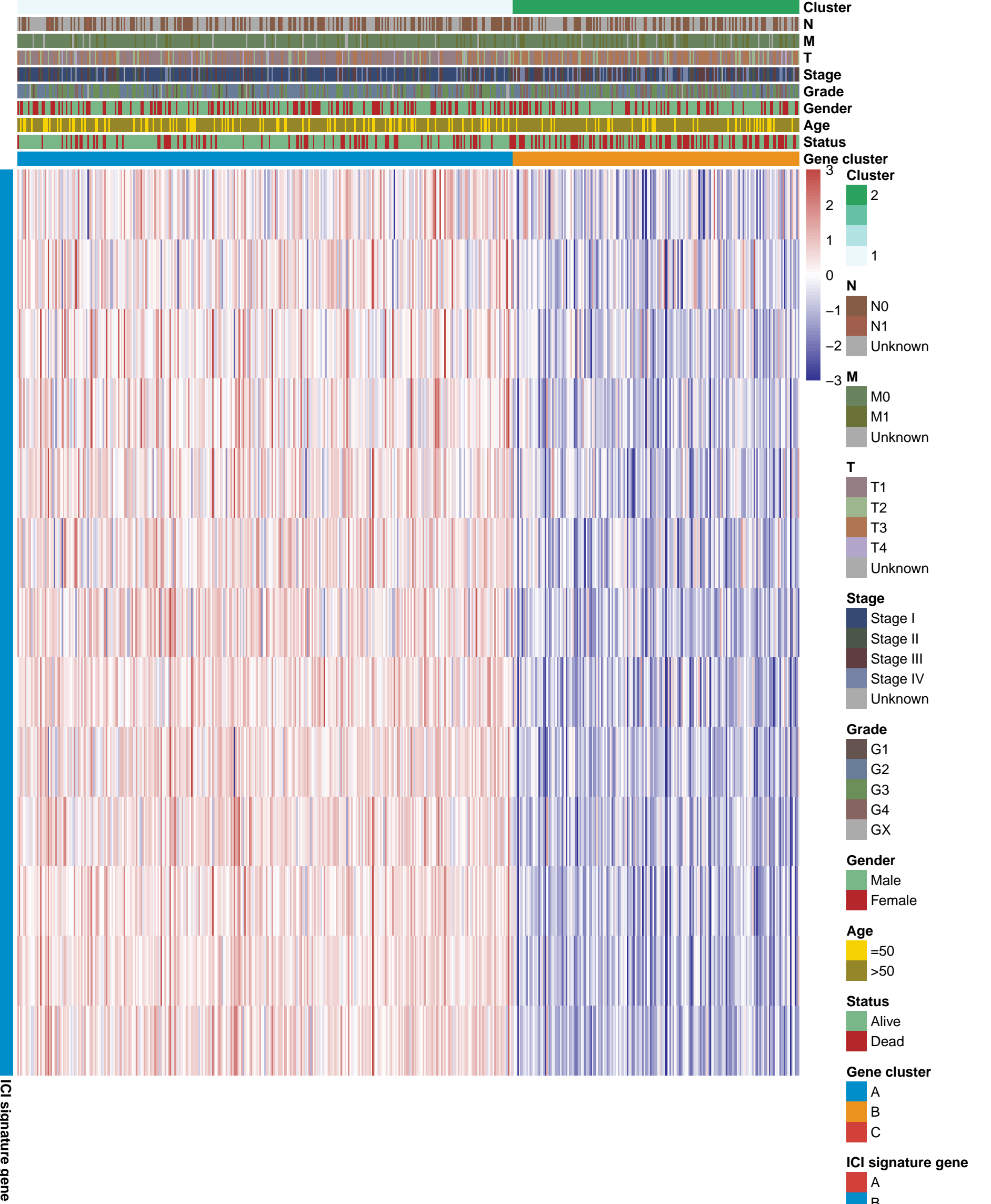

Supplement: Supplementary file 1 [file DataSheet_1.zip › Step1/ClusterCorrelation.pdf]

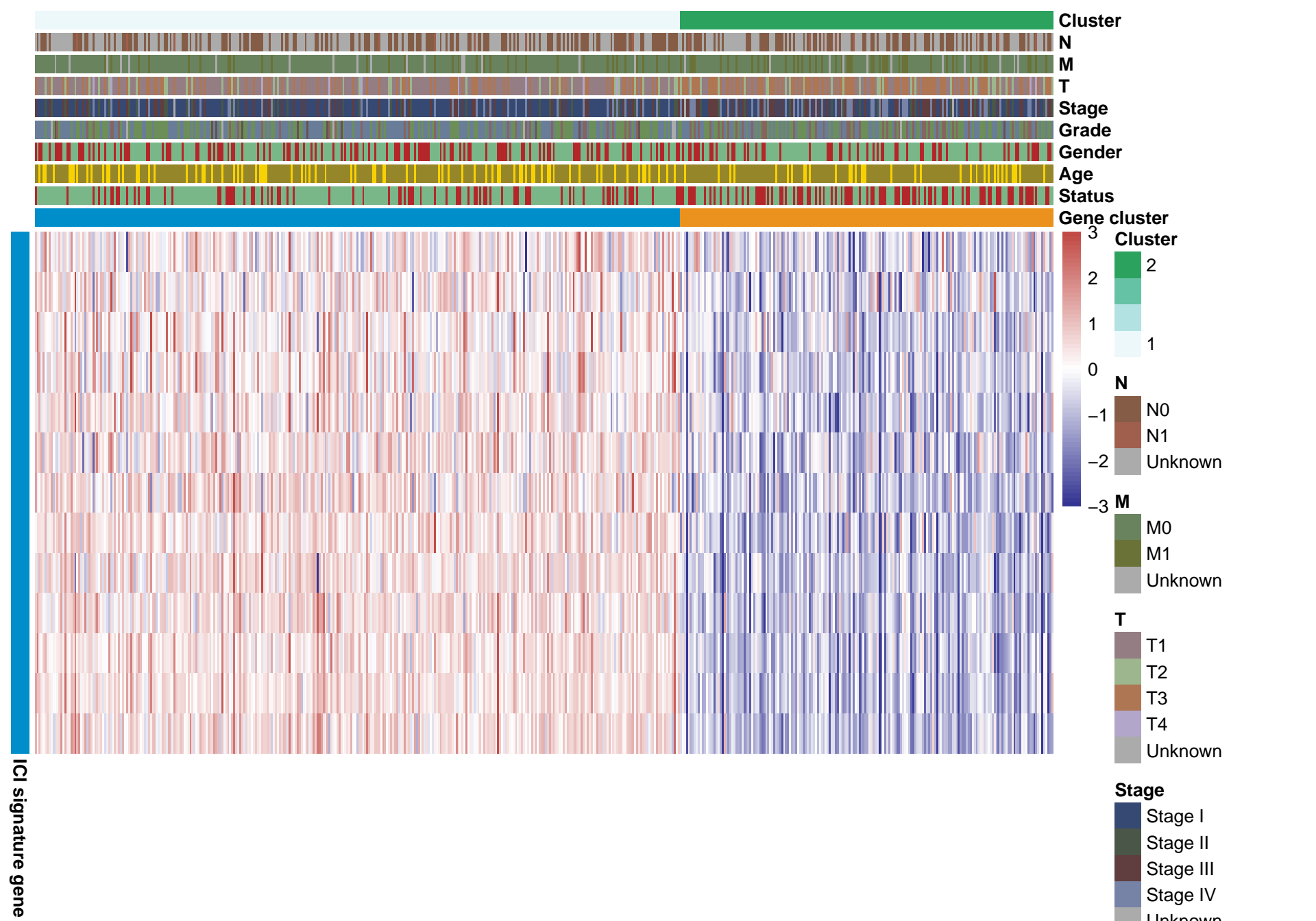

Supplement: Supplementary file 1 [file DataSheet_1.zip › Step1/PCA/ClusterCorrelation.pdf]

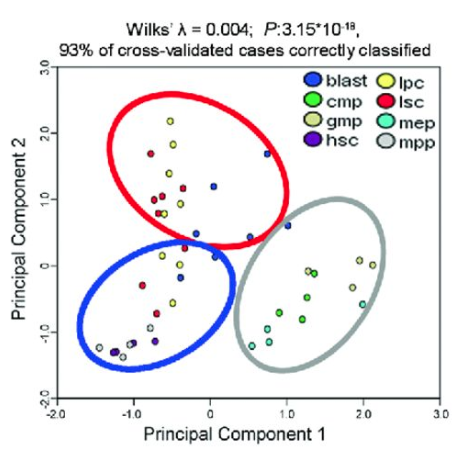

Supplement: Supplementary file 1 [file DataSheet_1.zip › Step1/PCA/PCA/demo1.png]

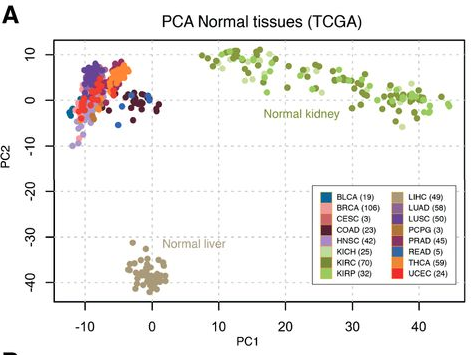

Supplement: Supplementary file 1 [file DataSheet_1.zip › Step1/PCA/PCA/demo2.png]

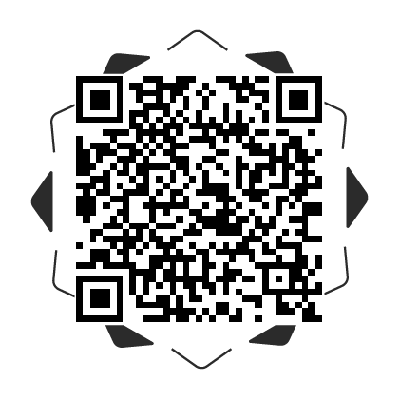

Supplement: Supplementary file 1 [file DataSheet_1.zip › Step1/PCA/PCA/hoptop.png]

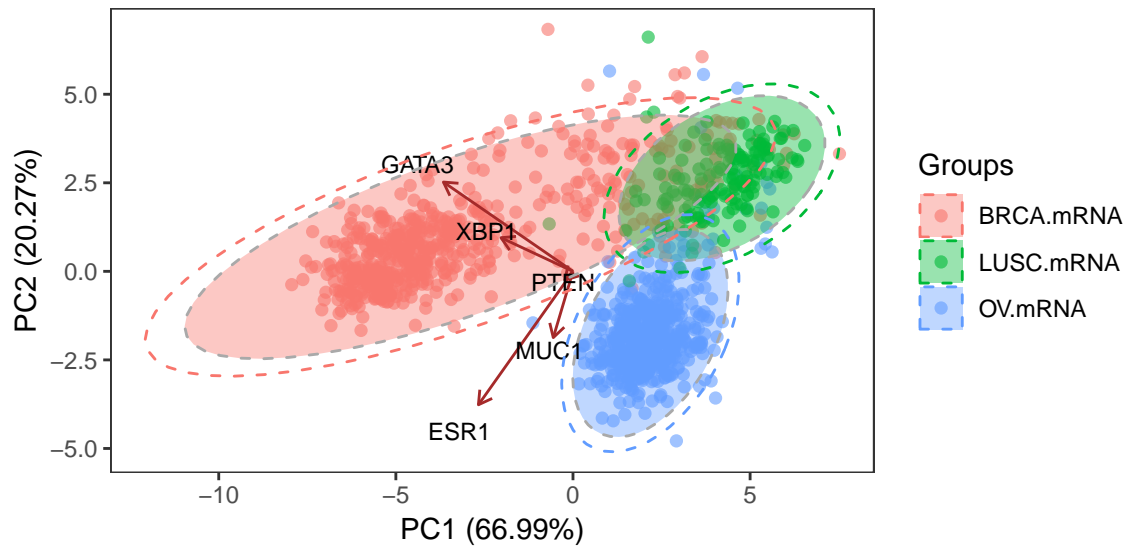

Supplement: Supplementary file 1 [file DataSheet_1.zip › Step1/PCA/PCA/PCA_arrow.pdf]

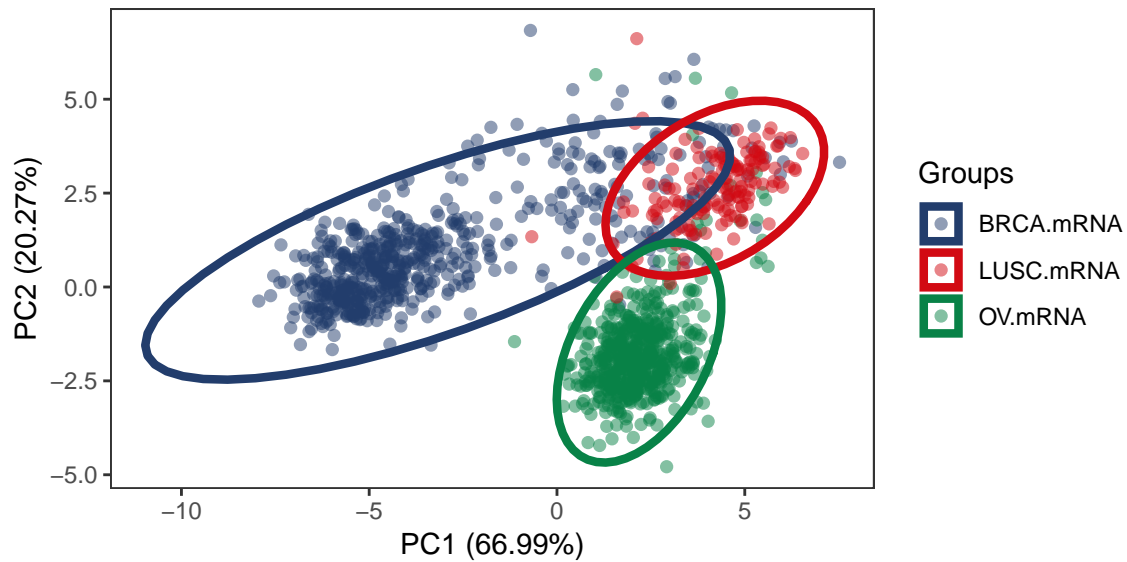

Supplement: Supplementary file 1 [file DataSheet_1.zip › Step1/PCA/PCA/PCA_classic.pdf]

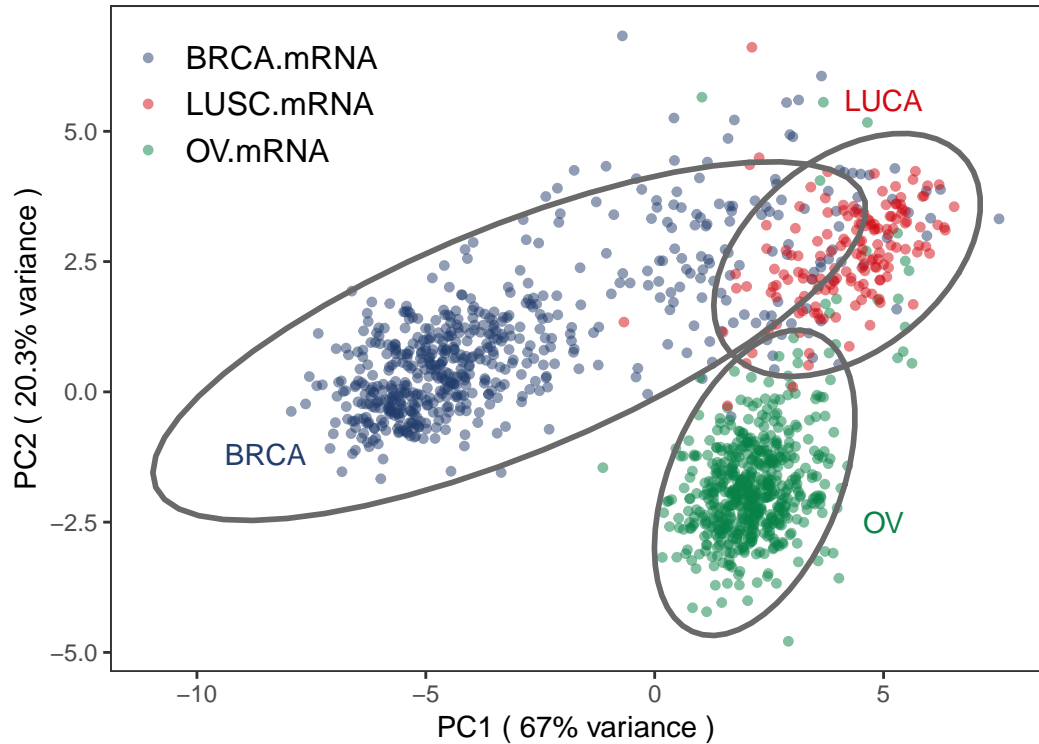

Supplement: Supplementary file 1 [file DataSheet_1.zip › Step1/PCA/PCA/PCA_DIY1.pdf]

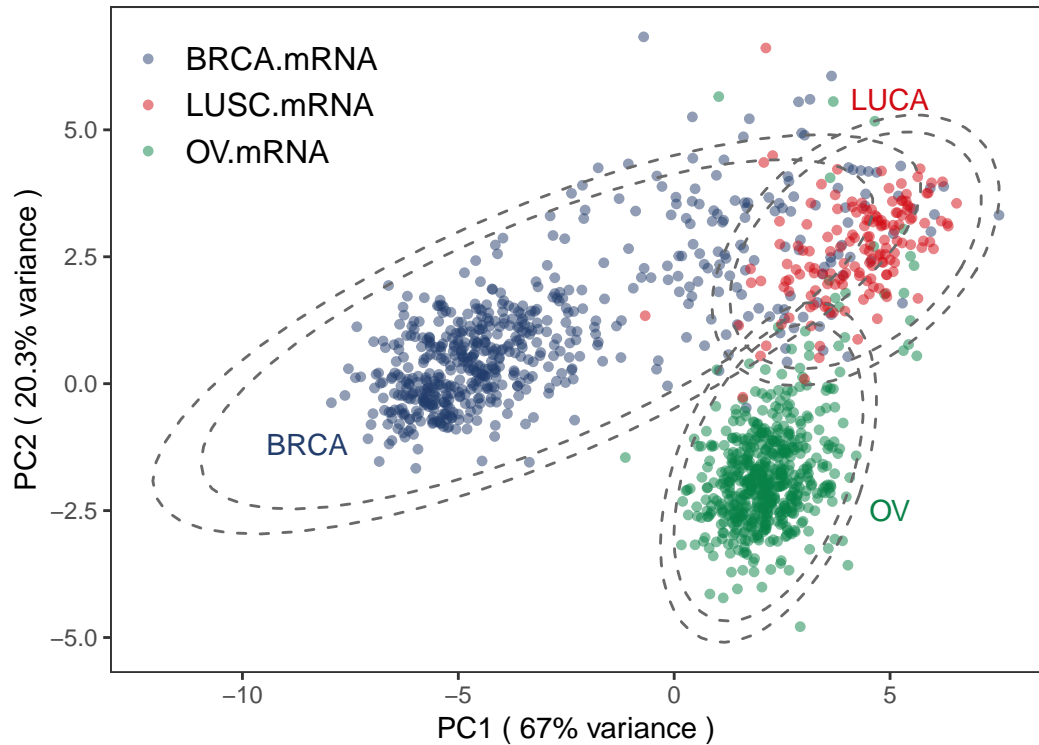

Supplement: Supplementary file 1 [file DataSheet_1.zip › Step1/PCA/PCA/PCA_DIY2.pdf]

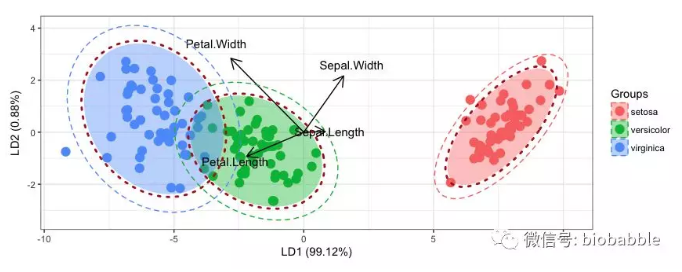

Supplement: Supplementary file 1 [file DataSheet_1.zip › Step1/PCA/PCA/╨í╚a╚a.png]

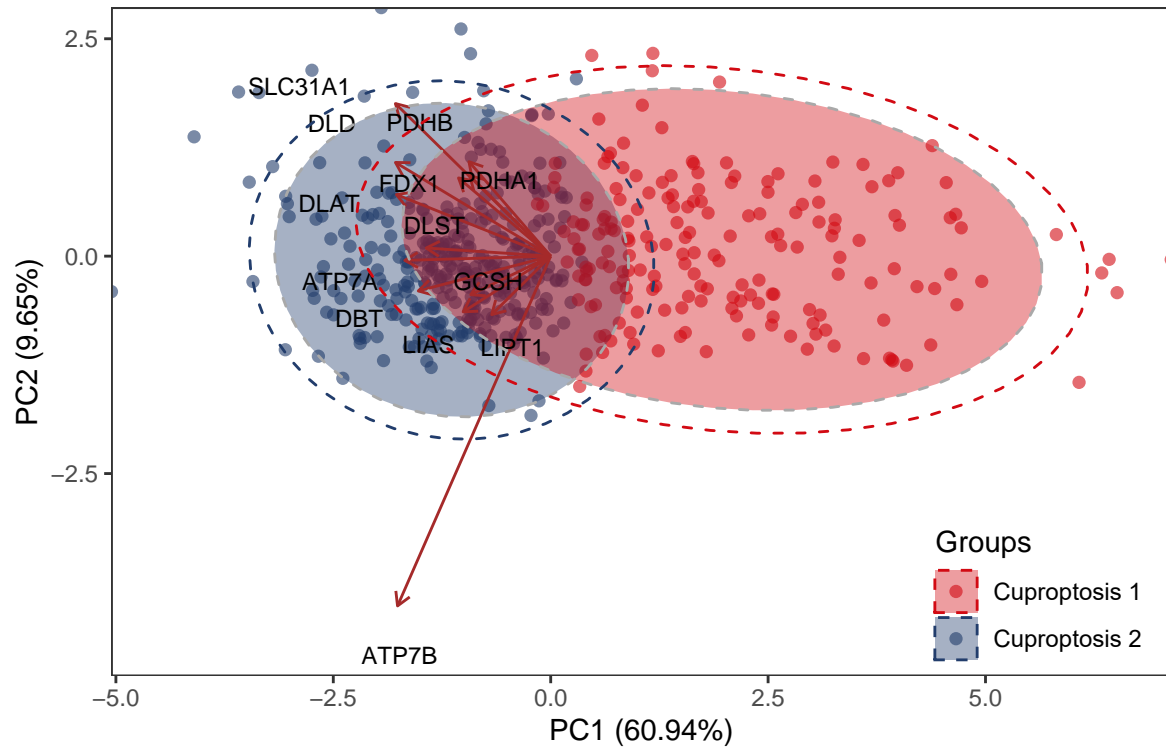

Supplement: Supplementary file 1 [file DataSheet_1.zip › Step1/PCA/PCA_arrow.pdf]

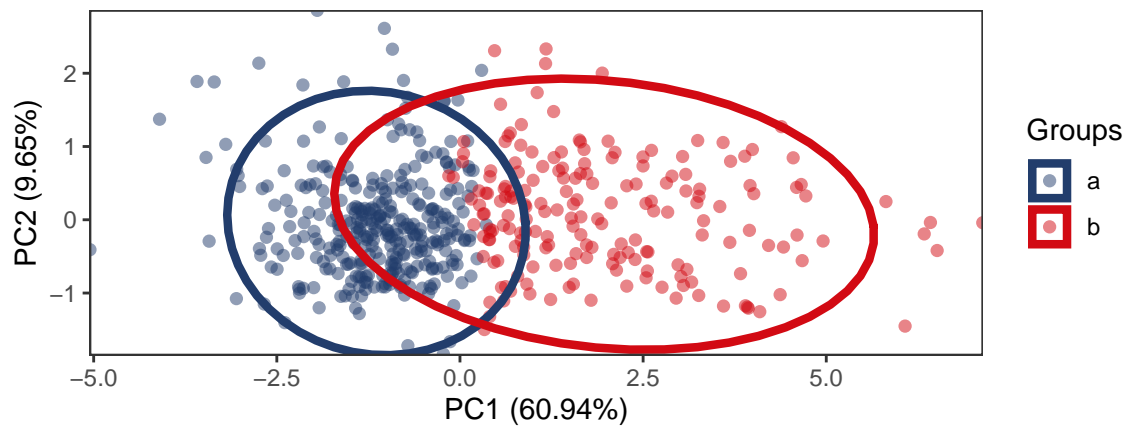

Supplement: Supplementary file 1 [file DataSheet_1.zip › Step1/PCA/PCA_classic.pdf]

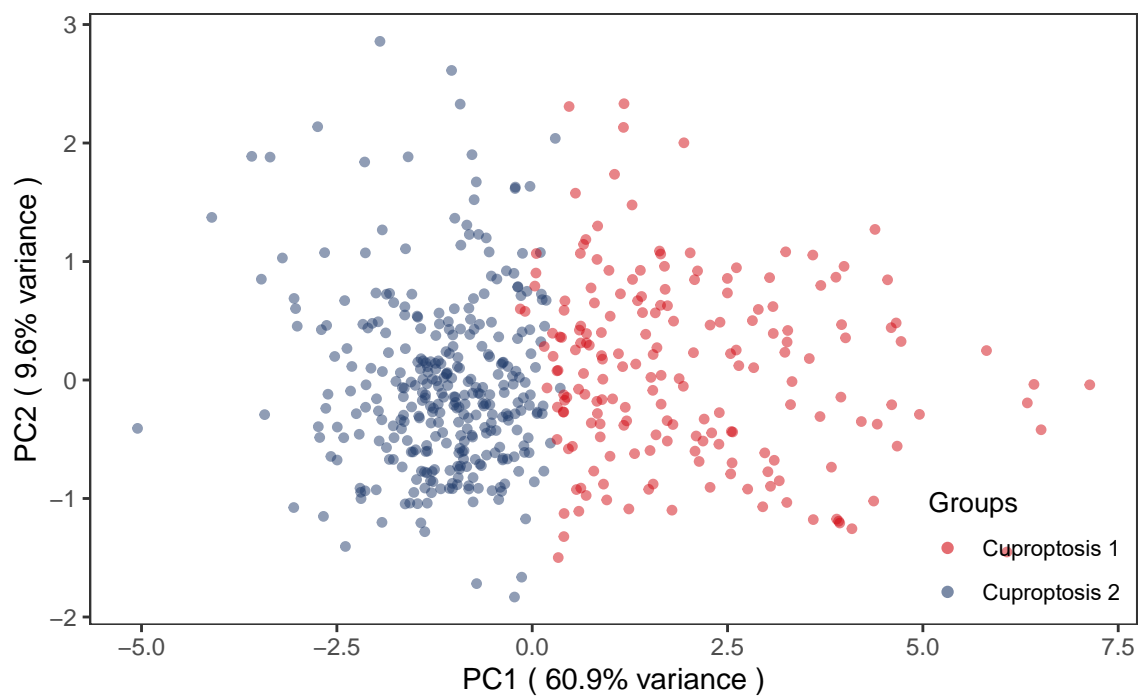

Supplement: Supplementary file 1 [file DataSheet_1.zip › Step1/PCA/PCA_DIY1.pdf]

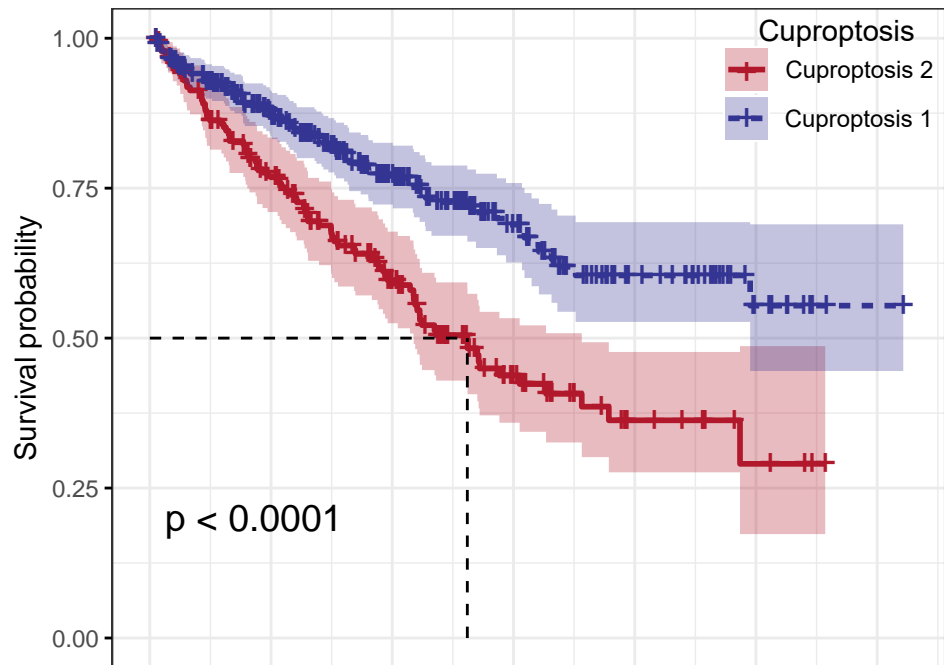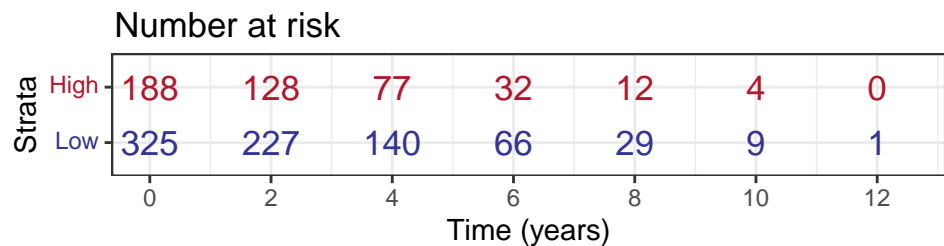

Supplement: Supplementary file 1 [file DataSheet_1.zip › Step1/su.pdf]

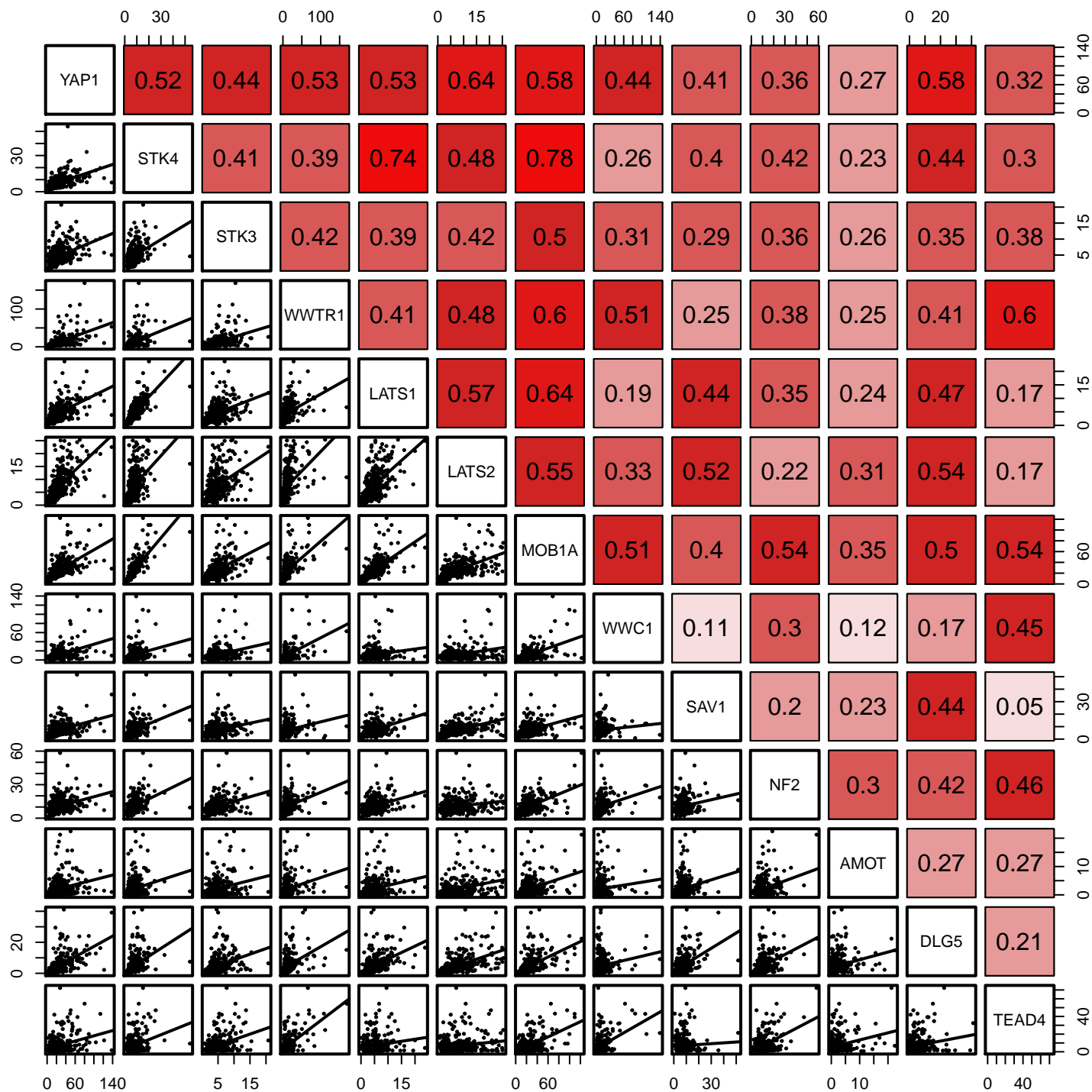

Supplement: Supplementary file 2 [file DataSheet_2.zip › Step2/corrgram/1.pdf]

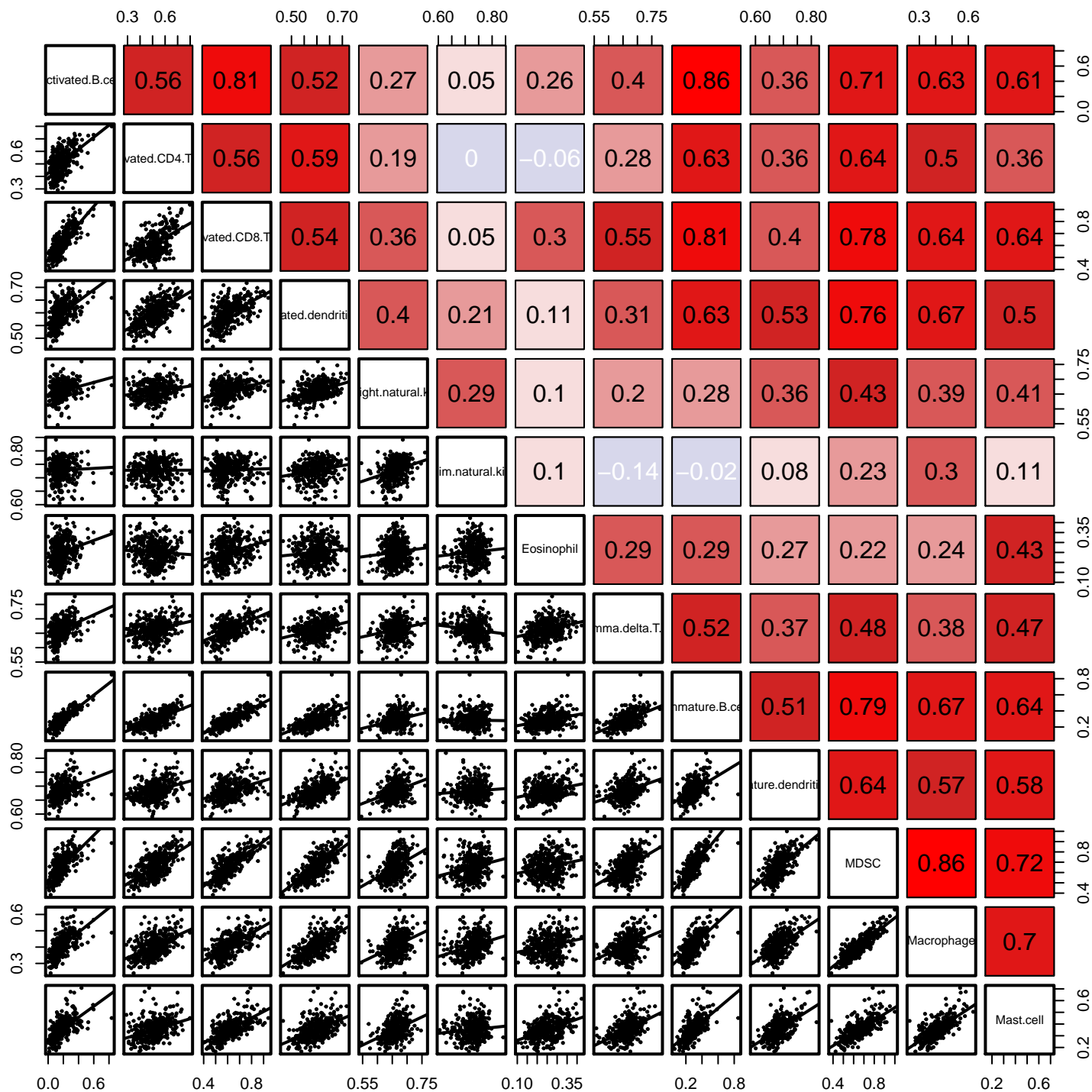

Supplement: Supplementary file 2 [file DataSheet_2.zip › Step2/corrgram/2.pdf]

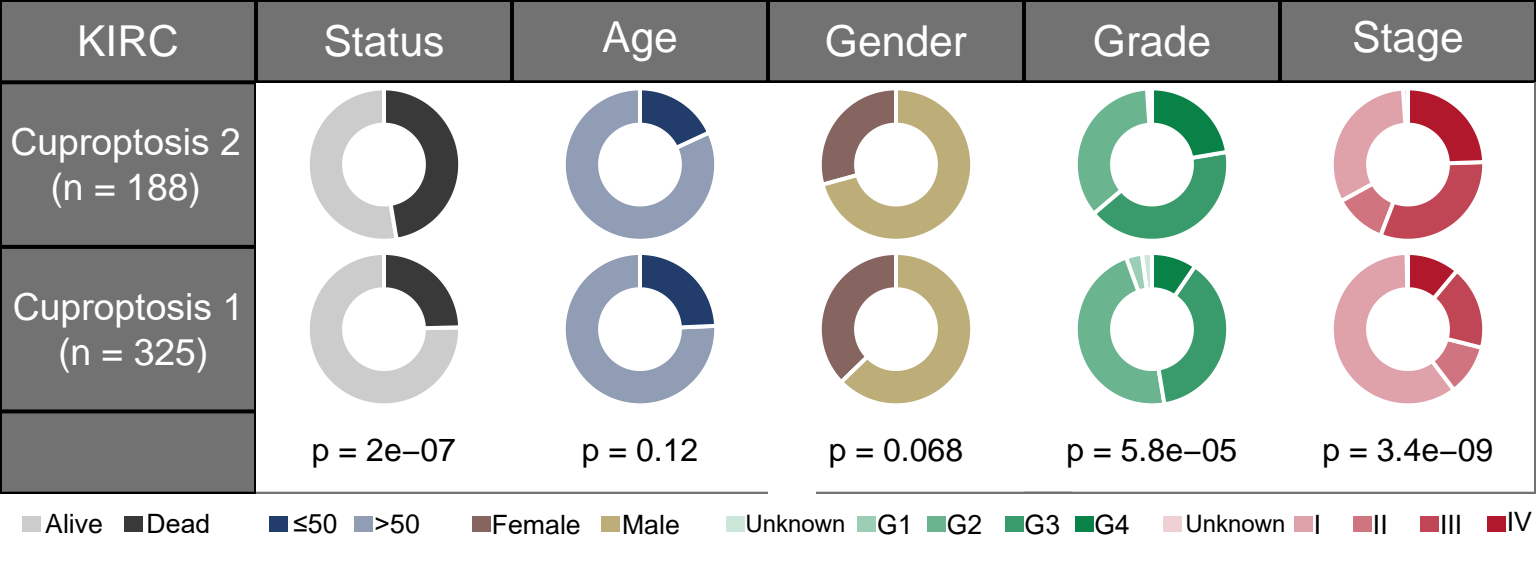

Supplement: Supplementary file 2 [file DataSheet_2.zip › Step2/Pan/pieTable.pdf]

Burden of Copy Number Gain (log2 transformed)

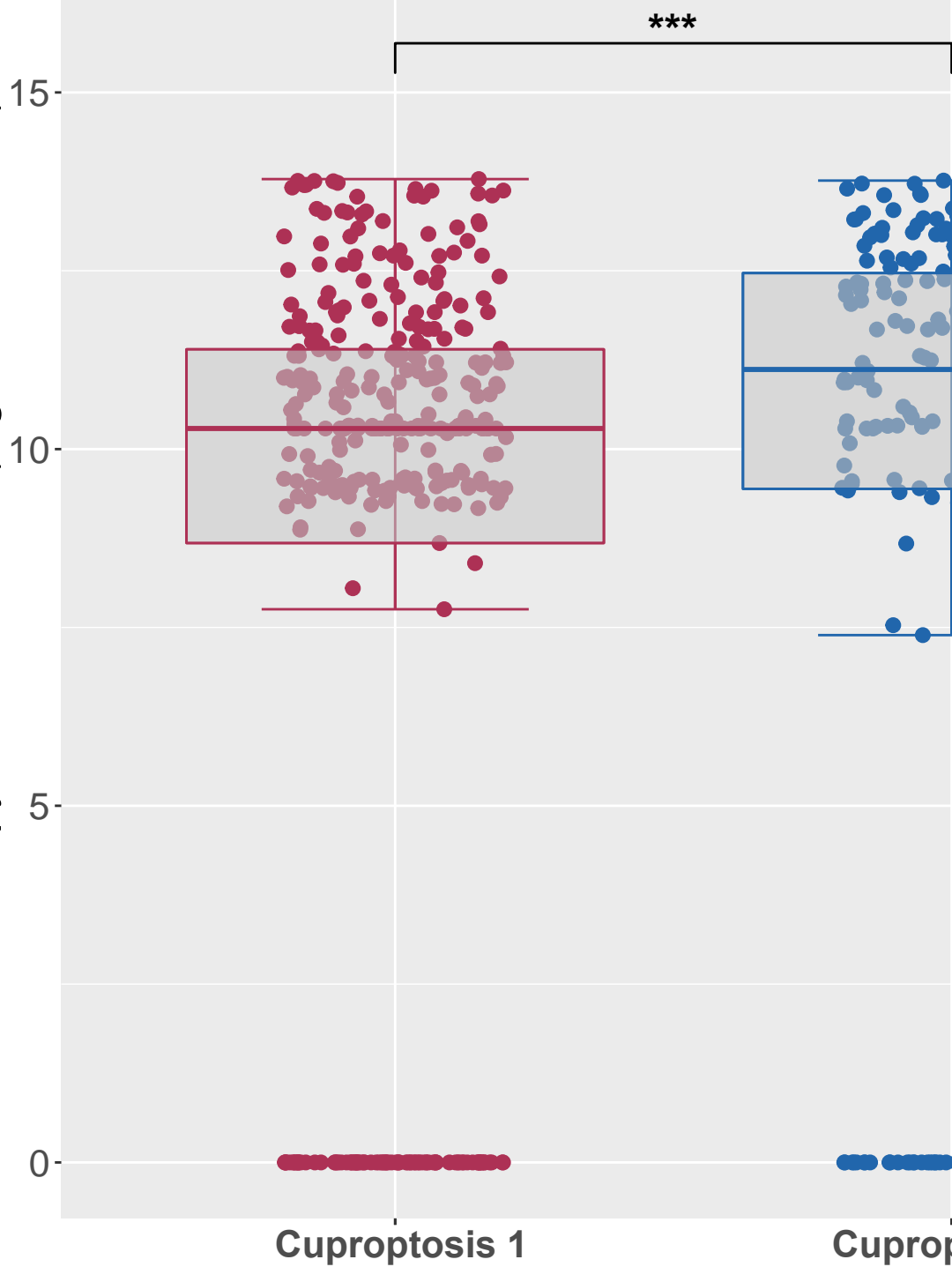

Supplement: Supplementary file 3 [file DataSheet_3.zip › Step3/cnvload/broad_gain_load.pdf]

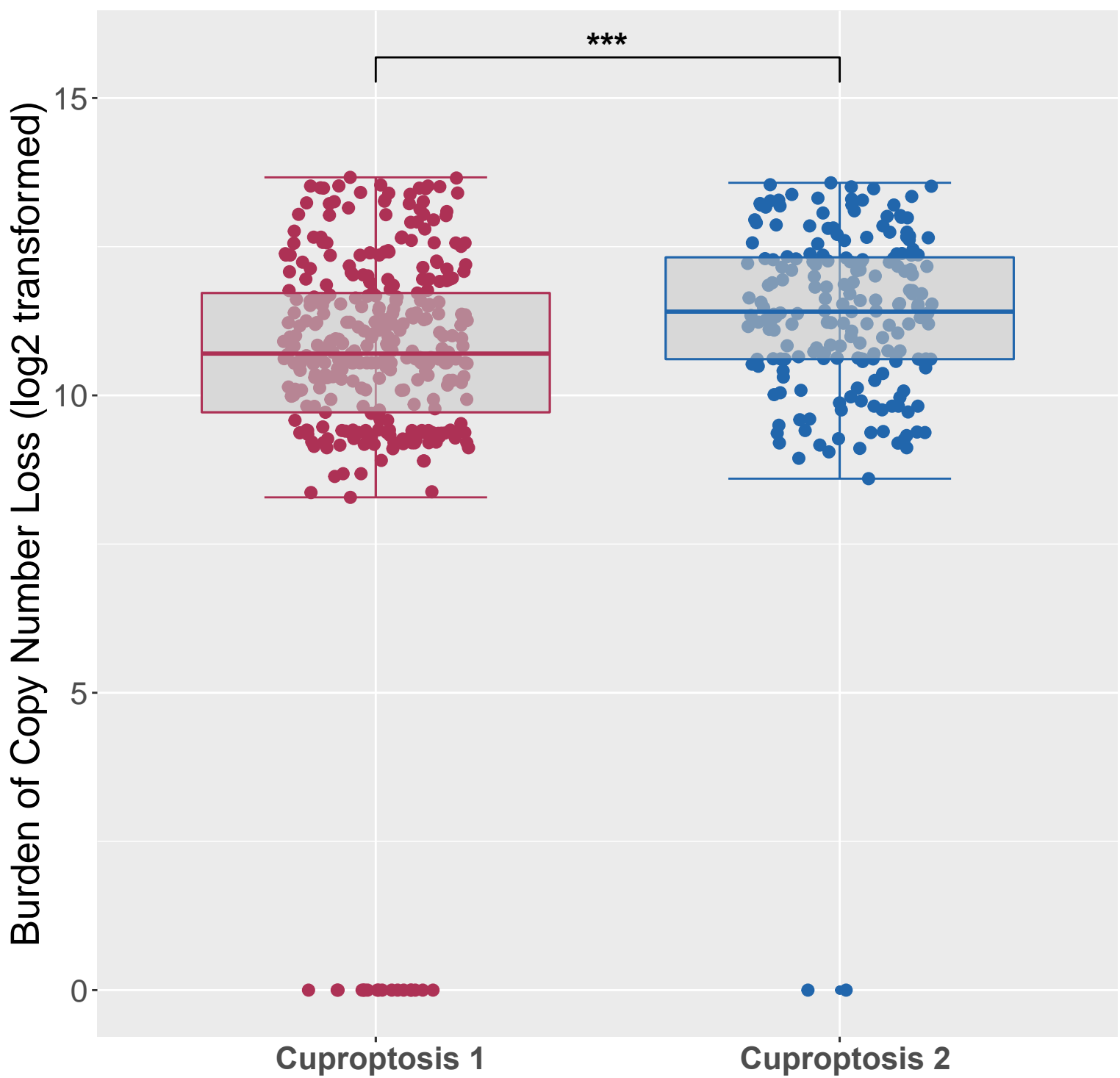

Supplement: Supplementary file 3 [file DataSheet_3.zip › Step3/cnvload/broad_loss_load.pdf]

Burden of Copy Number Gain (log2 transformed)

15  
10  
5  
0

Cuproptosis 1

Cuproptosis 2

\*\*\*

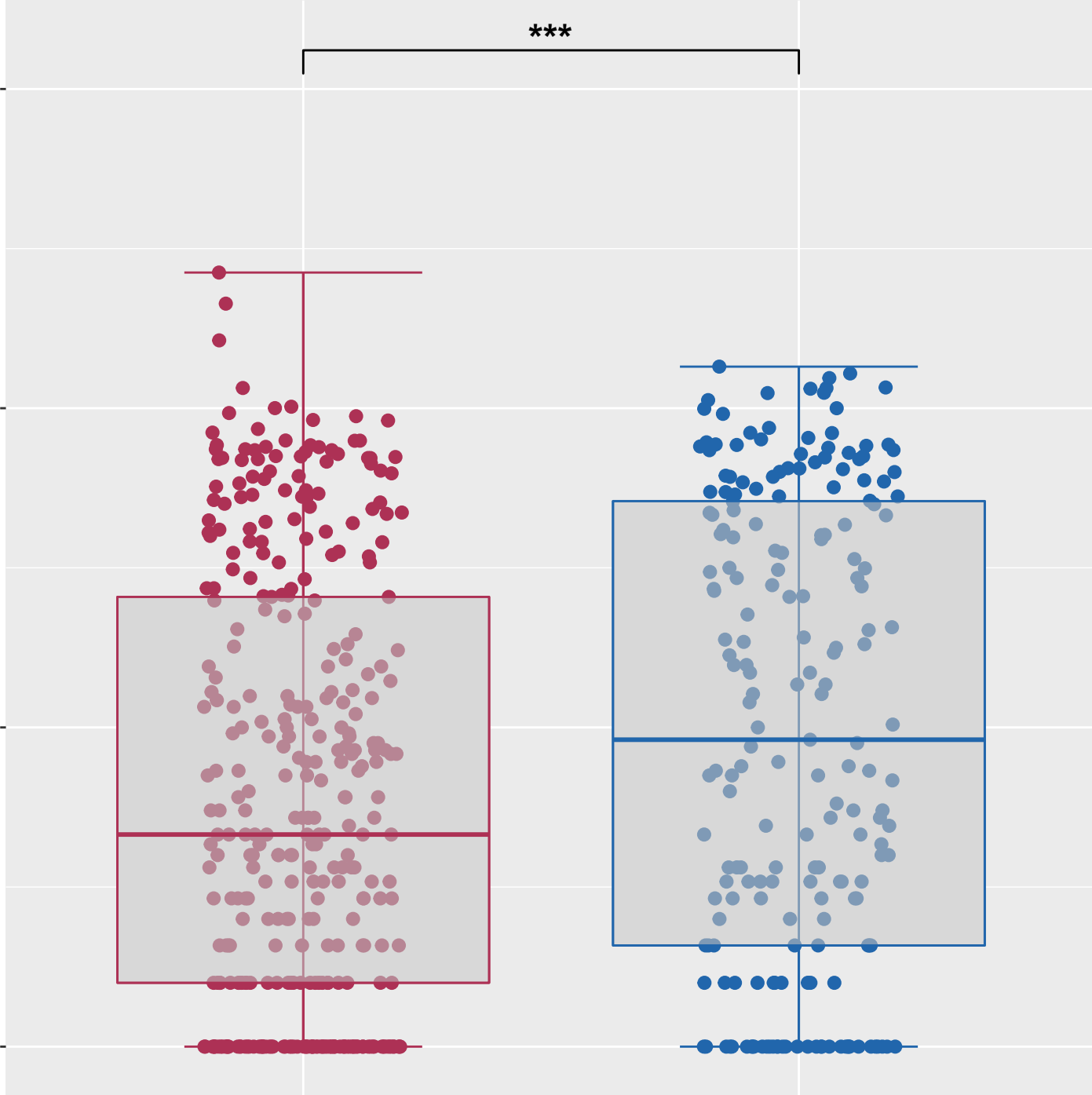

Supplement: Supplementary file 3 [file DataSheet_3.zip › Step3/cnvload/focal_gain_load.pdf]

Burden of Copy Number Loss (log2 transformed)

15  
10  
5  
0

Cuproptosis 1

Cuproptosis 2

\*\*\*

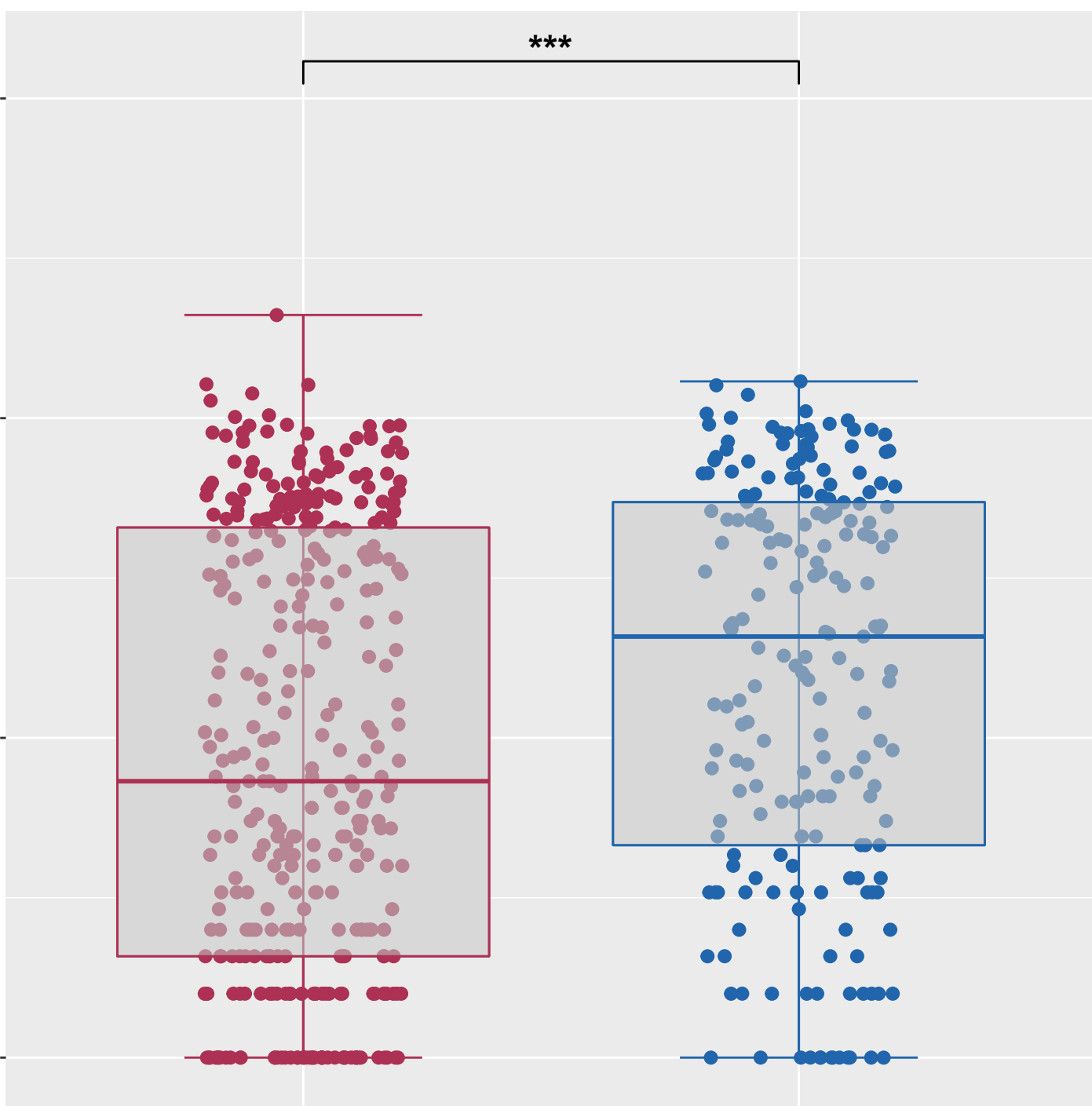

Supplement: Supplementary file 3 [file DataSheet_3.zip › Step3/cnvload/focal_loss_load.pdf]

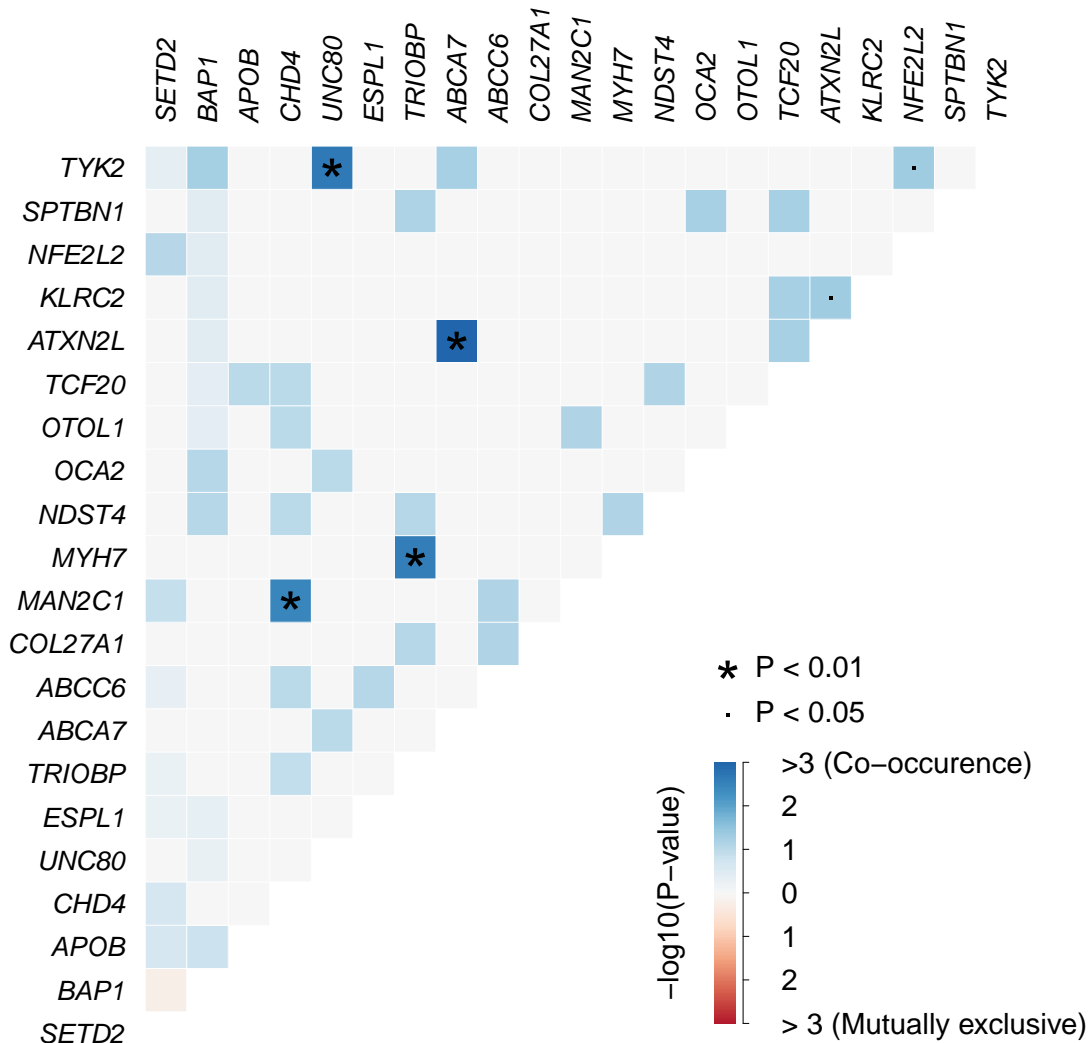

Supplement: Supplementary file 3 [file DataSheet_3.zip › Step3/mut/mutual exclusivity plot.pdf]

Non-synonymous mutation counts

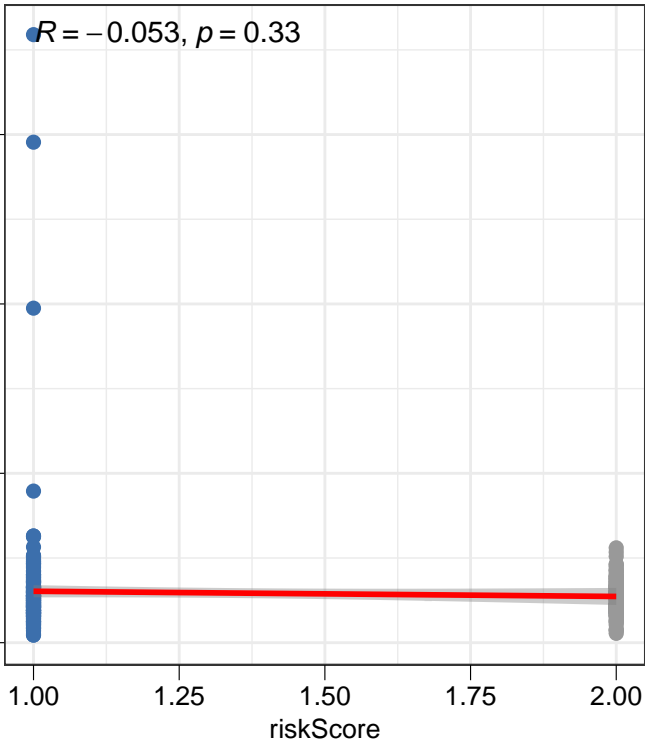

ns

•

•

•

•

•

•

Supplement: Supplementary file 3 [file DataSheet_3.zip › Step3/mut/scatter and boxplot for all mutation counts.pdf]

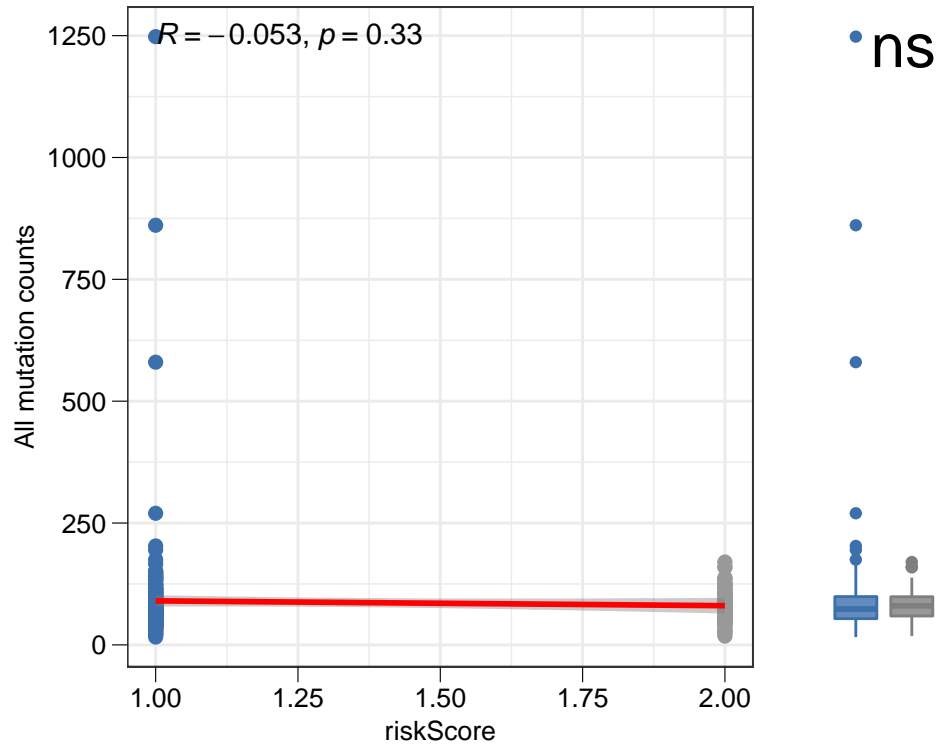

Supplement: Supplementary file 3 [file DataSheet_3.zip › Step3/mut/scatter and boxplot for synonymous mutation counts.pdf]

$\log_{10}(\text{TMB}+1)$

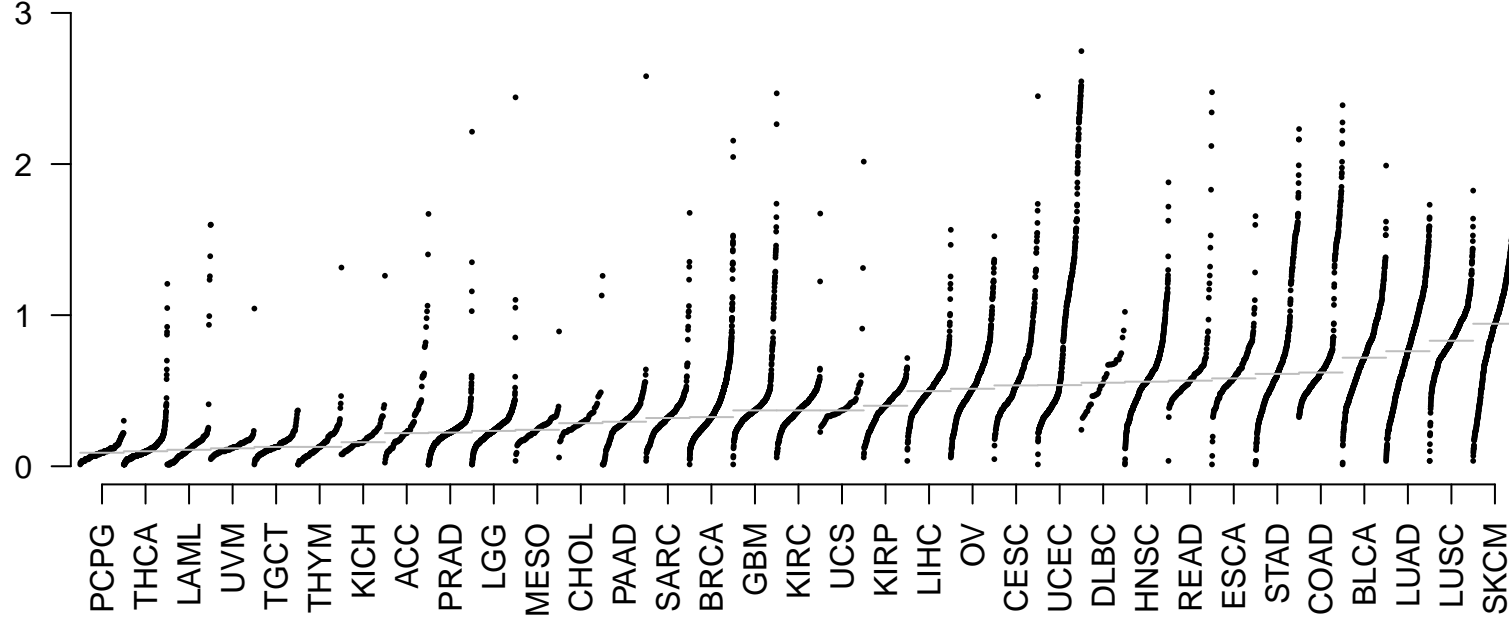

■ C>T  
■ T>C  
■ C>A  
■ C>G  
■ T>A  
■ T>G

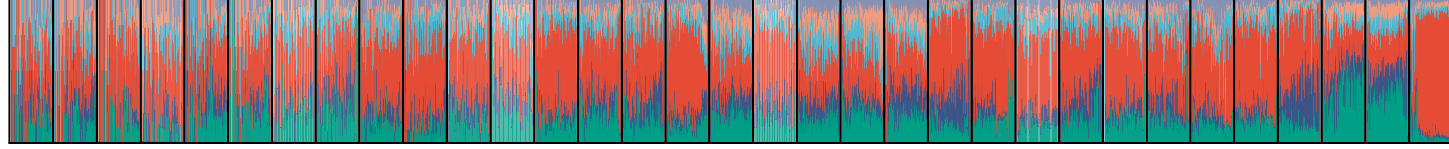

Supplement: Supplementary file 3 [file DataSheet_3.zip › Step3/mut/TMB_titv.pdf]

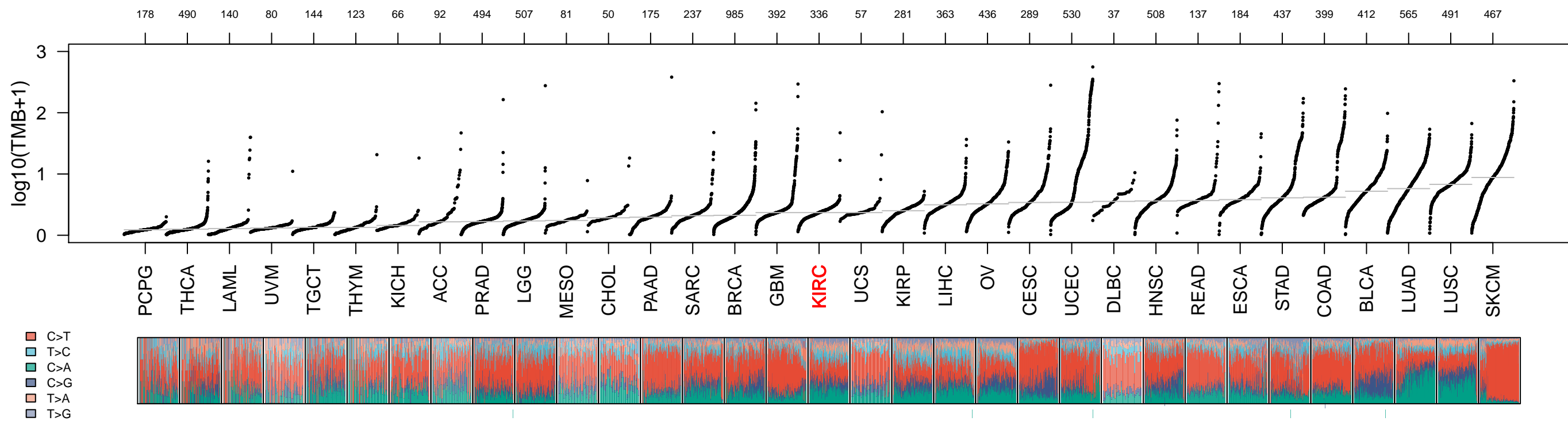

Supplement: Supplementary file 3 [file DataSheet_3.zip › Step3/mut/TMB_titv2.pdf]

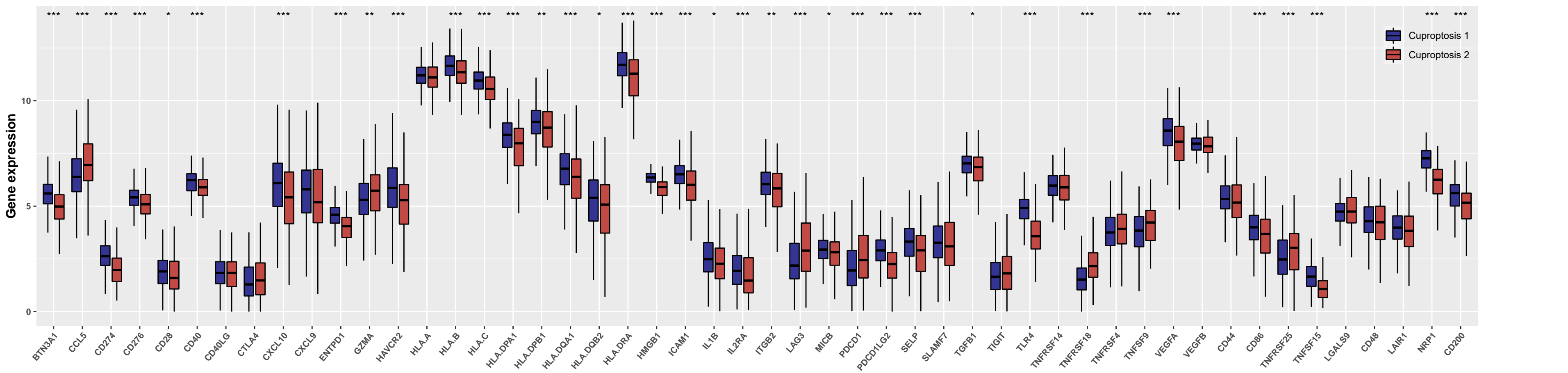

Supplement: Supplementary file 4 [file DataSheet_4.zip › Step4/check/diff.pdf]

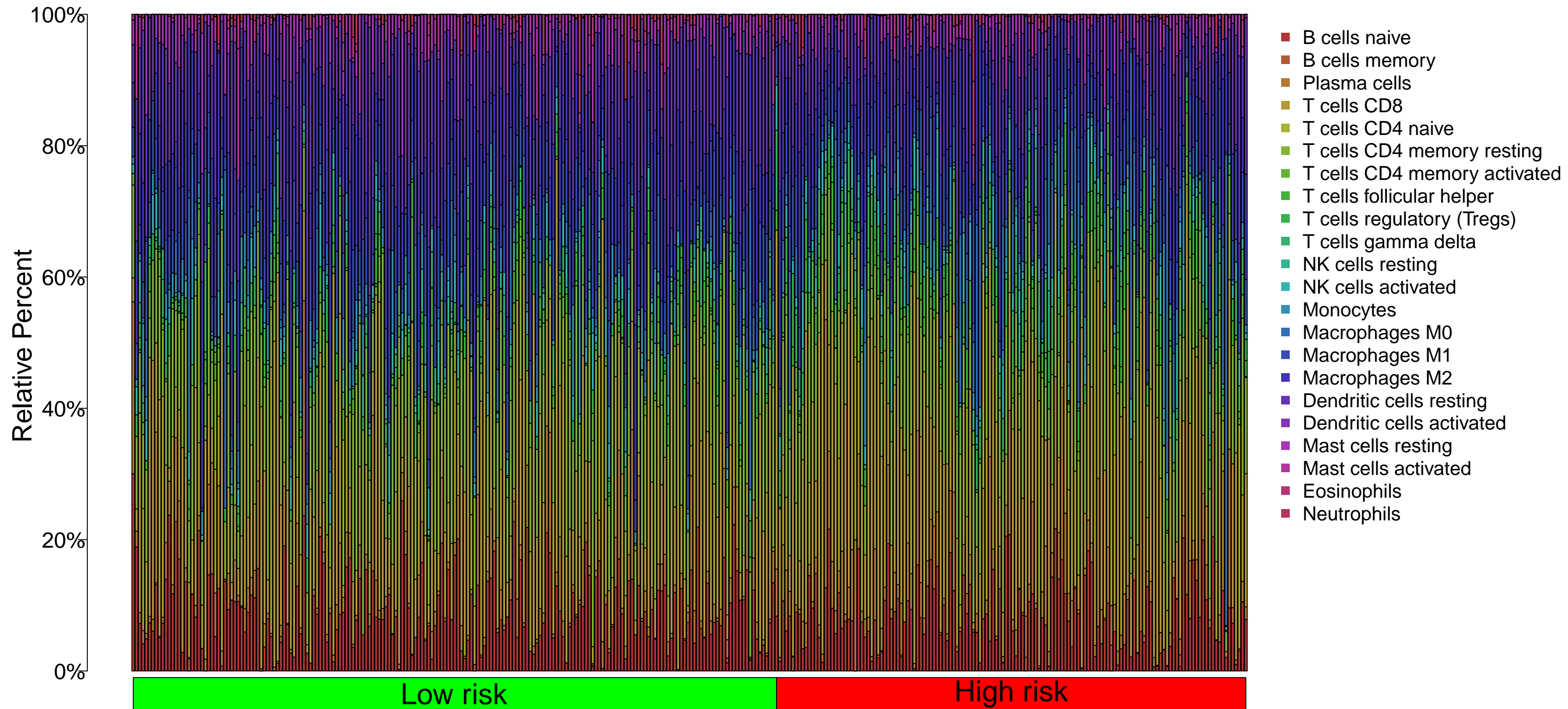

Supplement: Supplementary file 4 [file DataSheet_4.zip › Step4/immu/barplot.pdf]

CIBERSORT Score

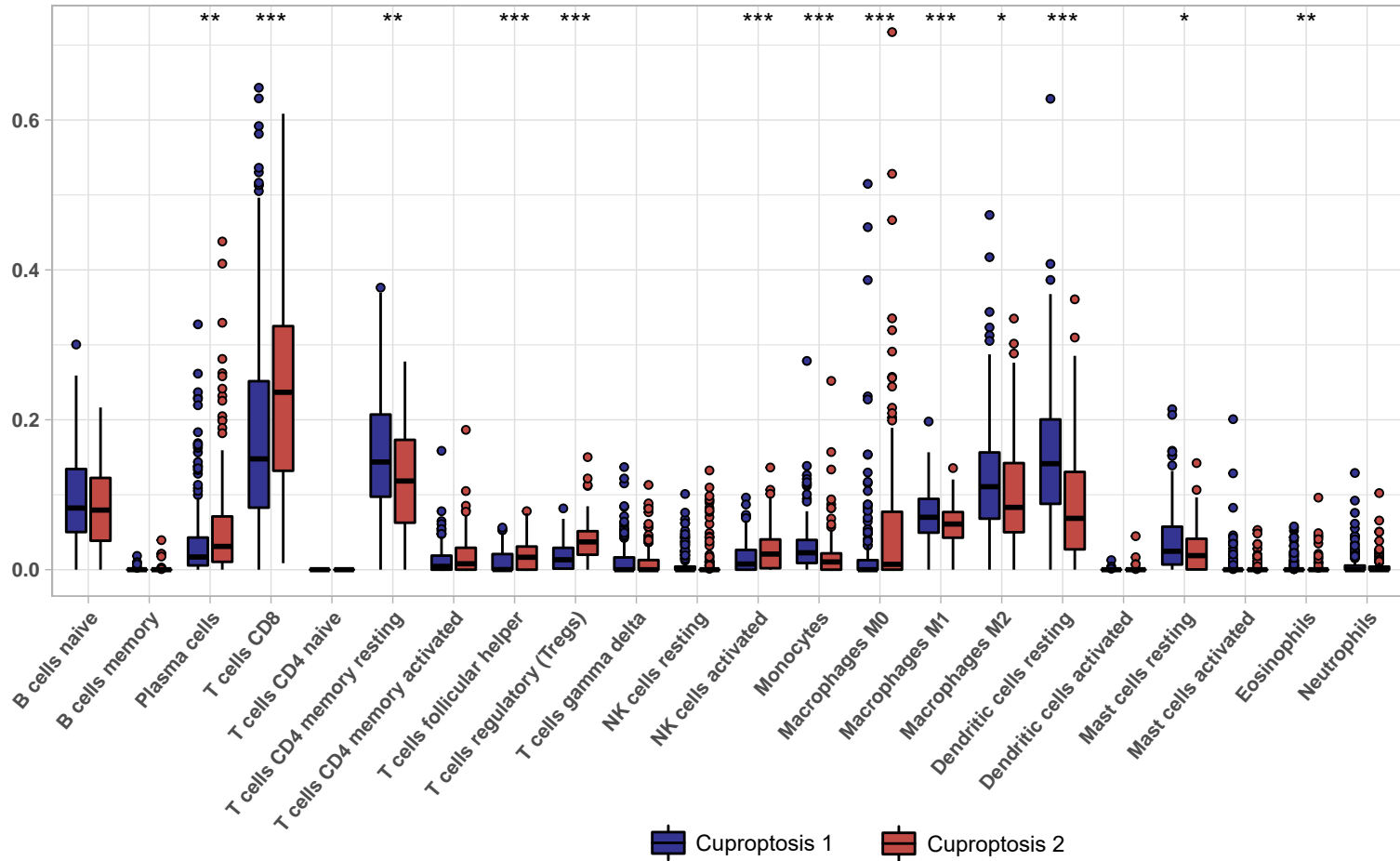

Supplement: Supplementary file 4 [file DataSheet_4.zip › Step4/immu/immune.diff.pdf]

■ High immunity  
■ Low immunity

31.38%

68.62%

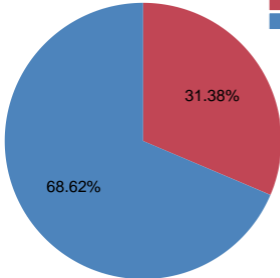

Supplement: Supplementary file 4 [file DataSheet_4.zip › Step4/immu/ssgsea/1.pdf]

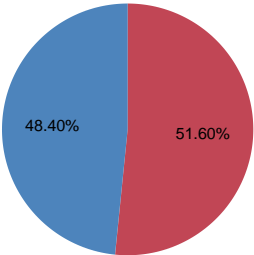

Supplement: Supplementary file 4 [file DataSheet_4.zip › Step4/immu/ssgsea/2.pdf]

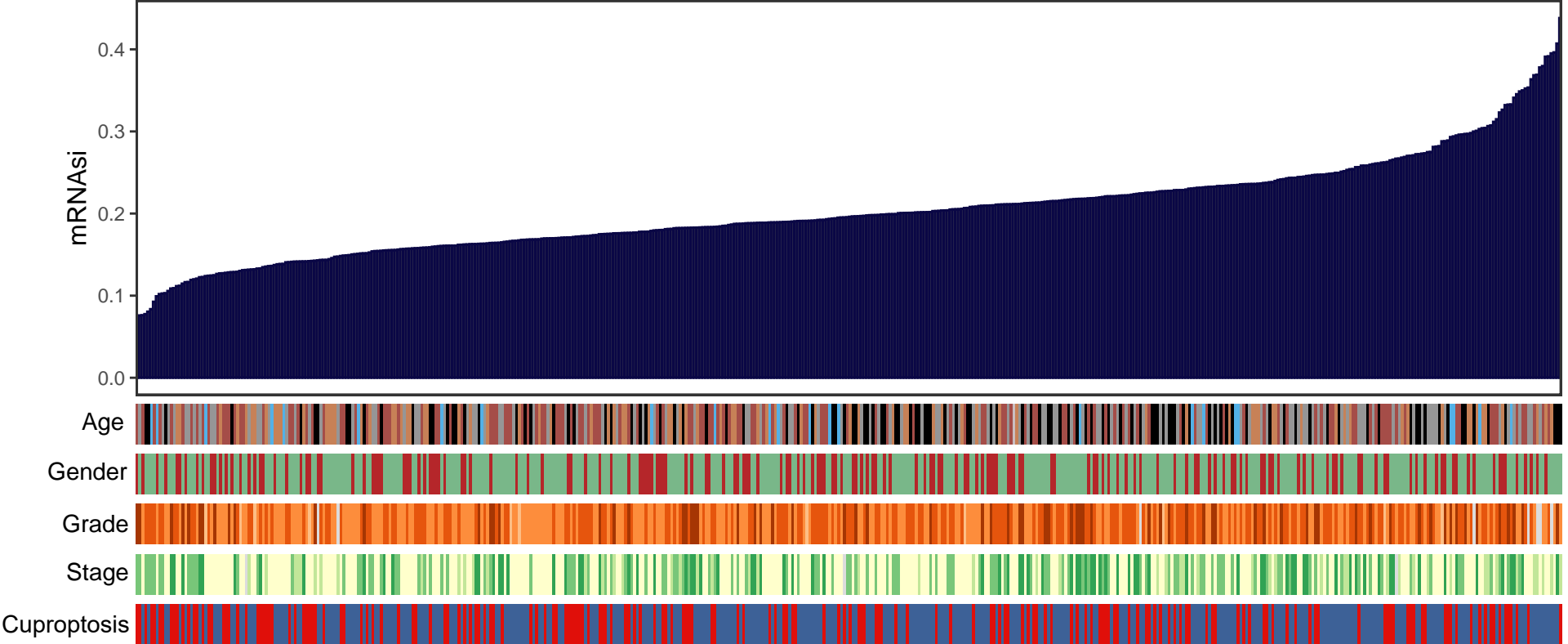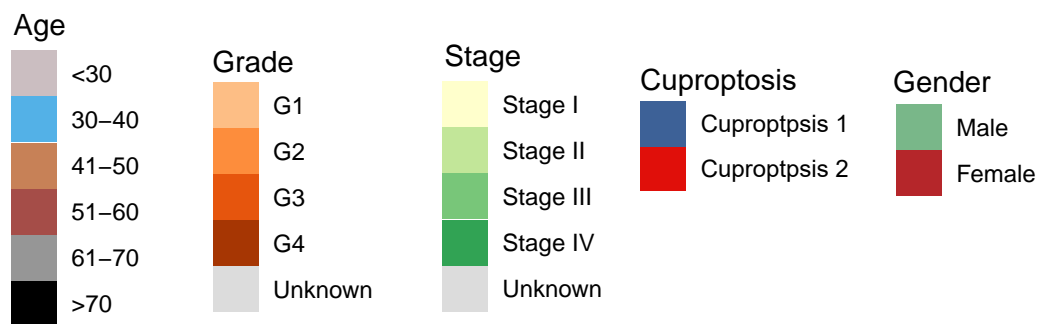

Supplement: Supplementary file 4 [file DataSheet_4.zip › Step4/mRNAsi/1.pdf]

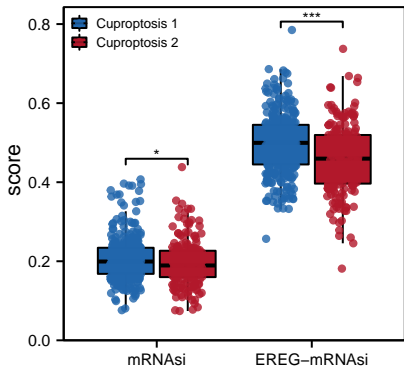

Supplement: Supplementary file 4 [file DataSheet_4.zip › Step4/mRNAsi/2.pdf]

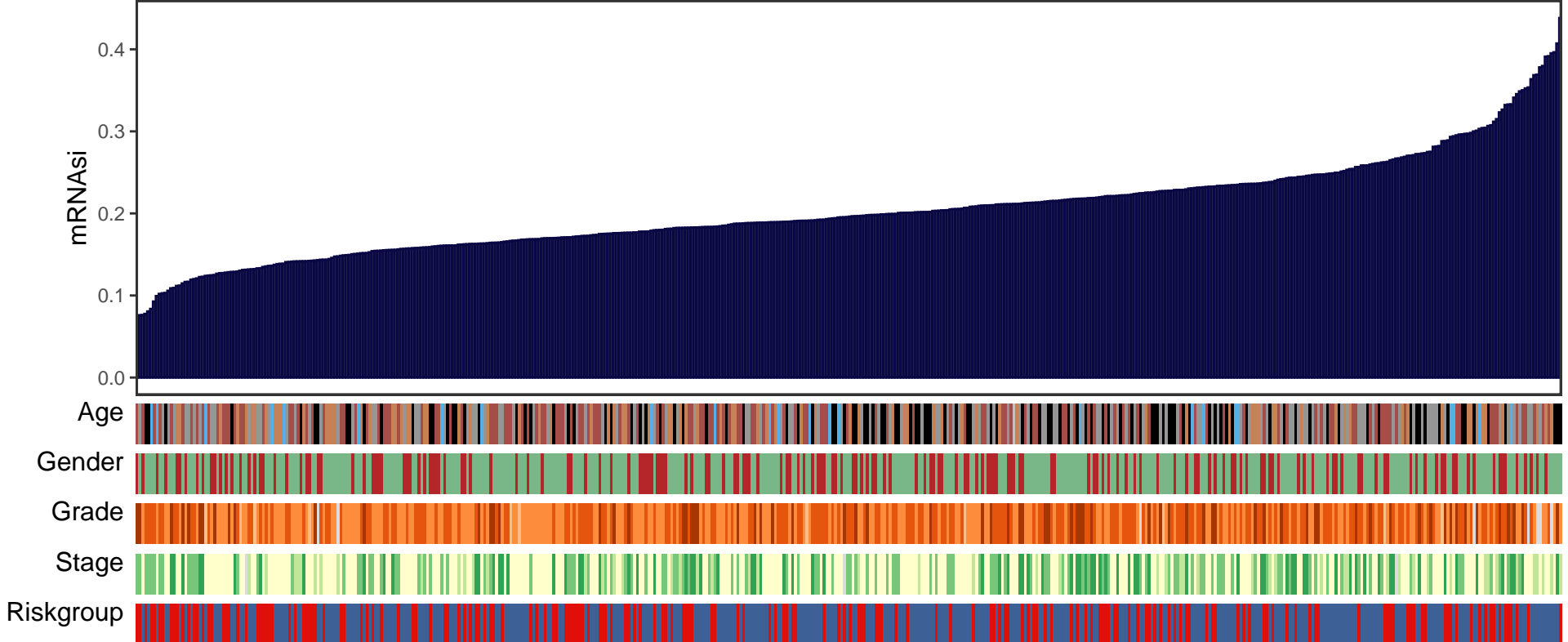

Age

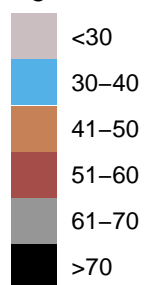

Gender

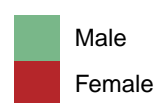

Grade

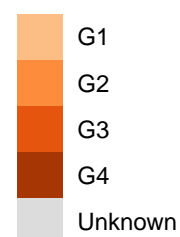

Stage

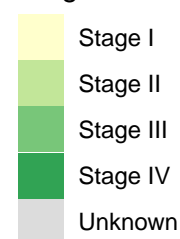

Riskgroup

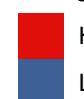

Supplement: Supplementary file 4 [file DataSheet_4.zip › Step4/mRNAsi/mRNAsi.pdf]

Stromal Score

2000  
1000  
0  
-1000

Cupropypsis 1

Cupropypsis 2

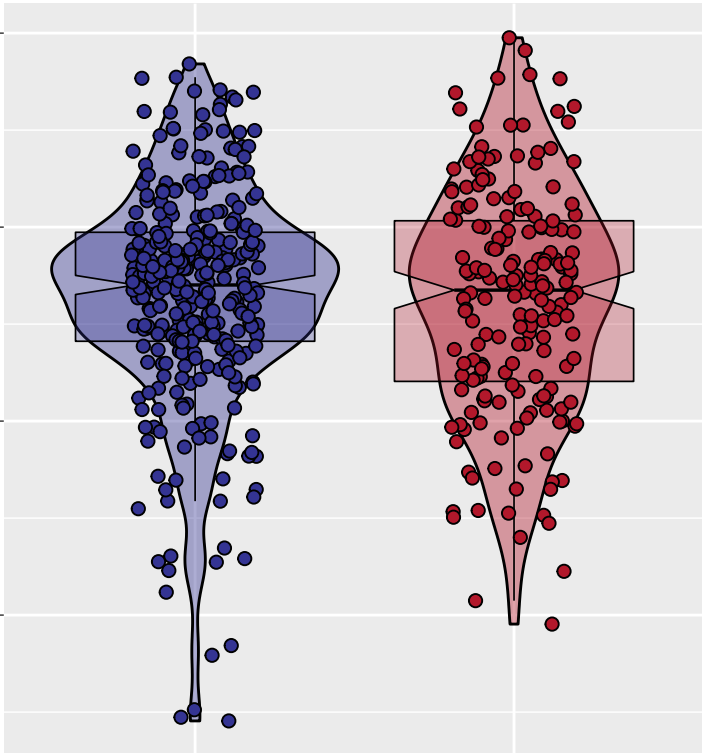

Supplement: Supplementary file 4 [file DataSheet_4.zip › Step4/score/1.pdf]

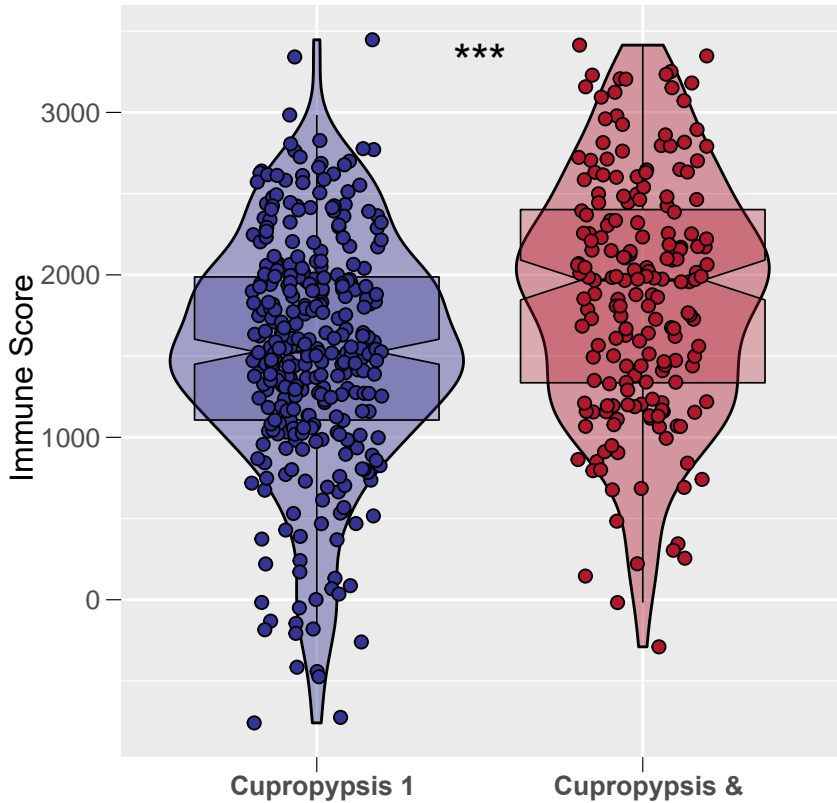

Supplement: Supplementary file 4 [file DataSheet_4.zip › Step4/score/2.pdf]

ESTIMATE Score

\*\*

4000

2000

0

-2000

Cupropypsis 1

Cupropypsis &

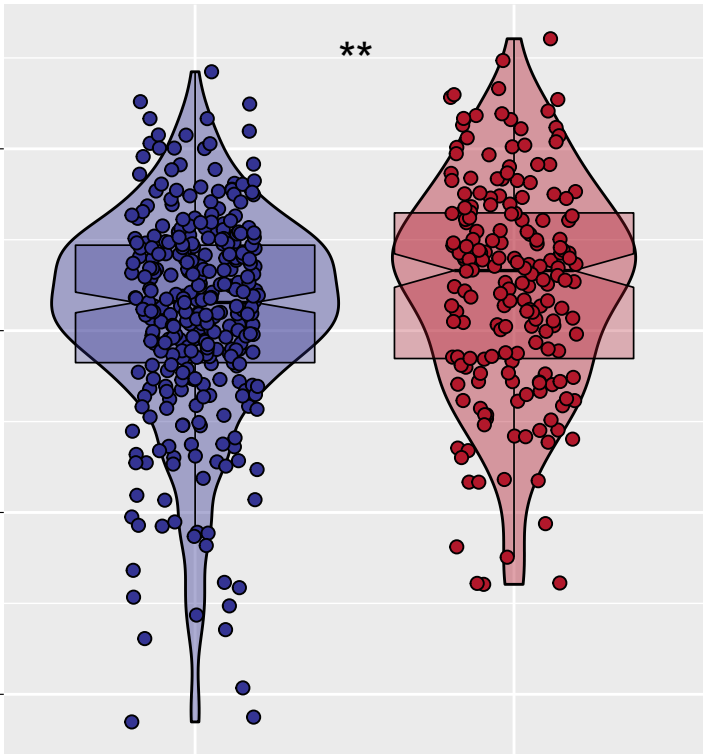

Supplement: Supplementary file 4 [file DataSheet_4.zip › Step4/score/3.pdf]

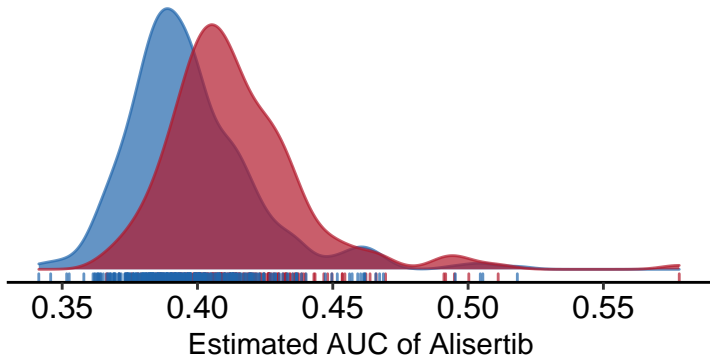

Cuproptosis 2

Cuproptosis 1

\*\*\*

Supplement: Supplementary file 5 [file DataSheet_5.zip › Step5/auc/alisertib.pdf]

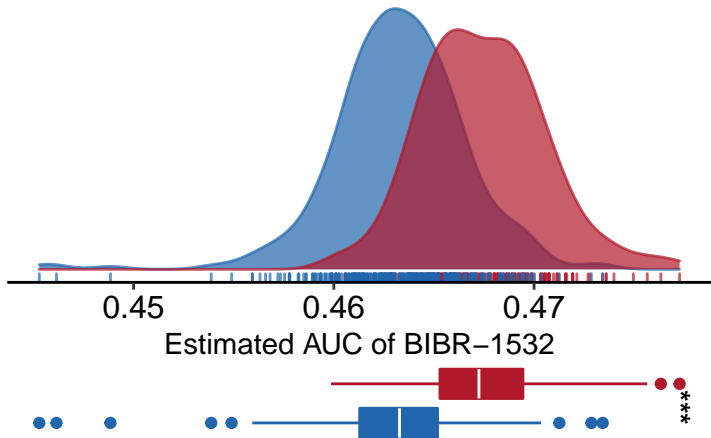

Supplement: Supplementary file 5 [file DataSheet_5.zip › Step5/auc/BIBR-1532.pdf]

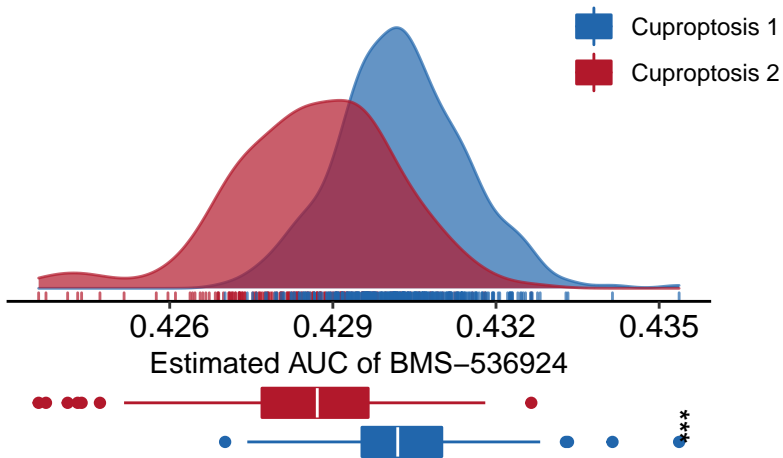

Supplement: Supplementary file 5 [file DataSheet_5.zip › Step5/auc/BMS-536924.pdf]

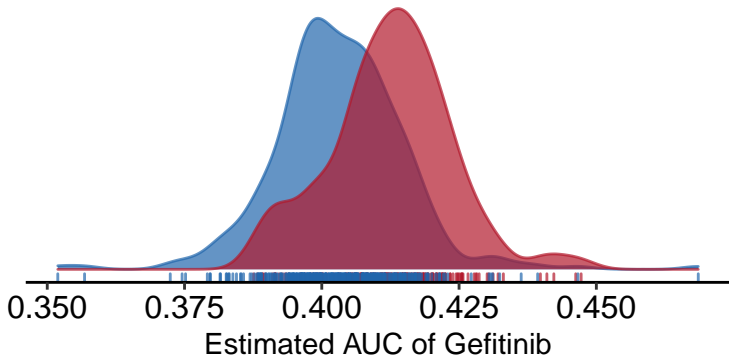

Cuproptosis 2

Cuproptosis 1

\*\*\*

Supplement: Supplementary file 5 [file DataSheet_5.zip › Step5/auc/Gefitinib.pdf]

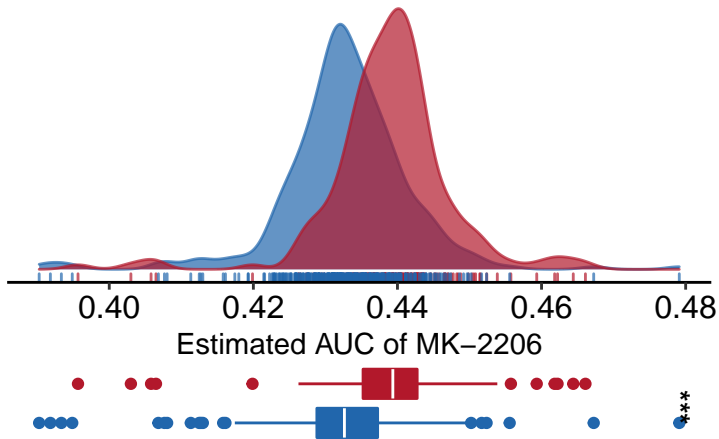

Supplement: Supplementary file 5 [file DataSheet_5.zip › Step5/auc/MK-2206.pdf]

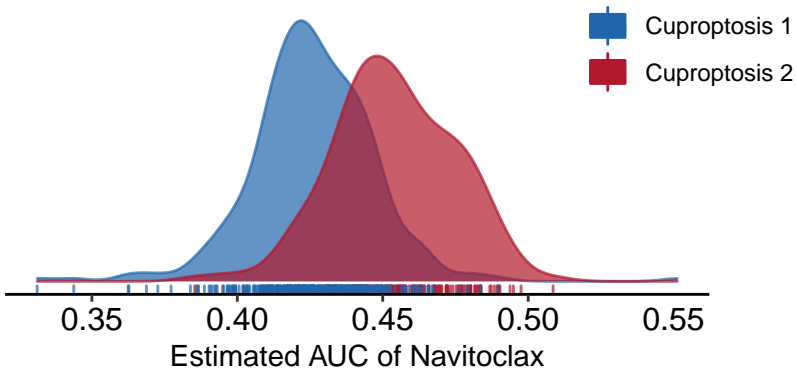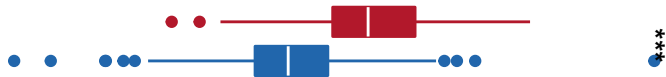

Supplement: Supplementary file 5 [file DataSheet_5.zip › Step5/auc/Navitoclax.pdf]

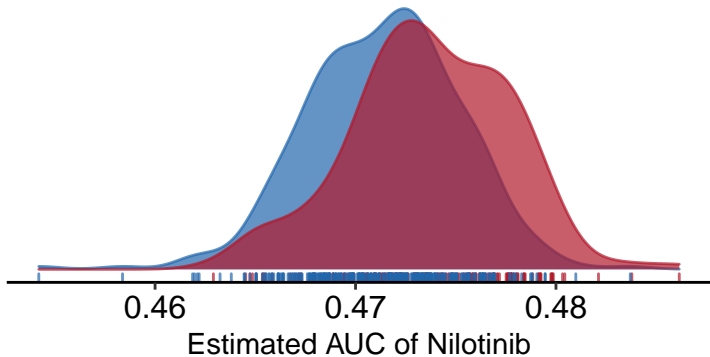

Cuproptosis 2

Cuproptosis 1

\*\*\*

Supplement: Supplementary file 5 [file DataSheet_5.zip › Step5/auc/Nilotinib.pdf]

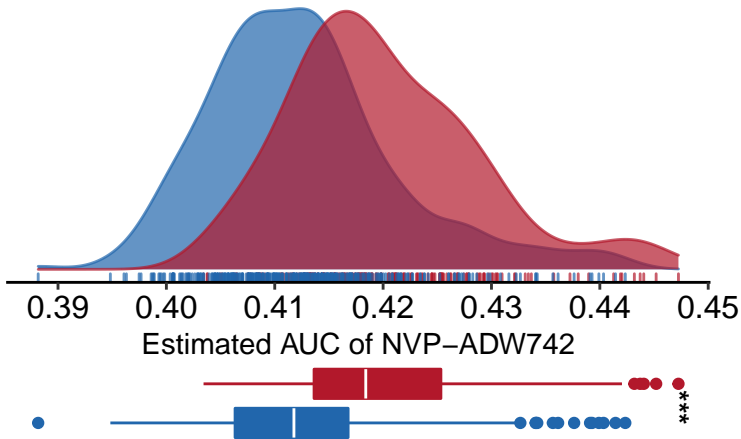

Supplement: Supplementary file 5 [file DataSheet_5.zip › Step5/auc/NVP-ADW742.pdf]

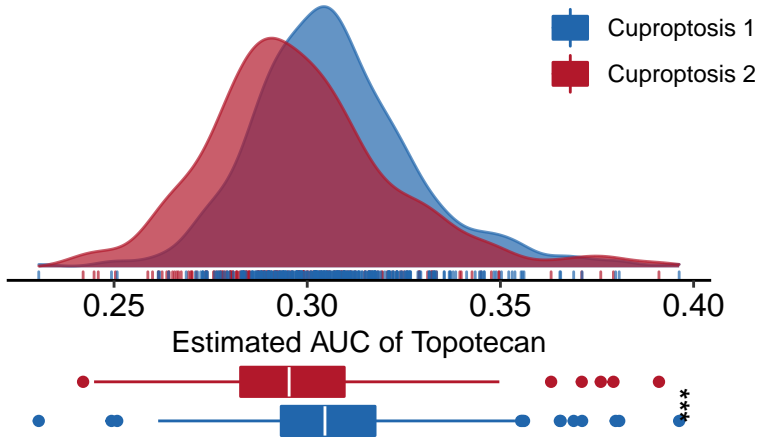

Supplement: Supplementary file 5 [file DataSheet_5.zip › Step5/auc/Topotecan.pdf]

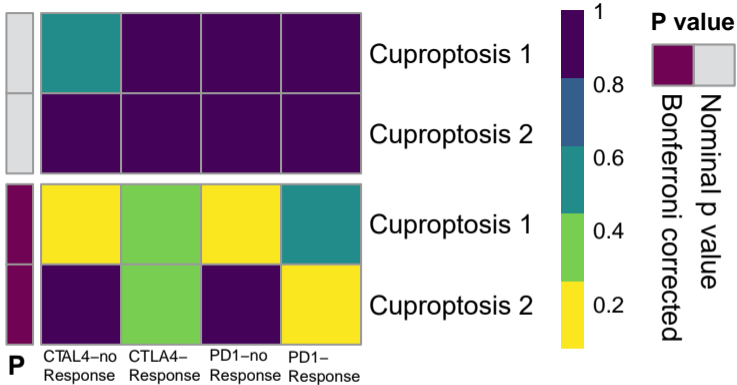

Supplement: Supplementary file 5 [file DataSheet_5.zip › Step5/map/1 - ╕▒▒╛.pdf]

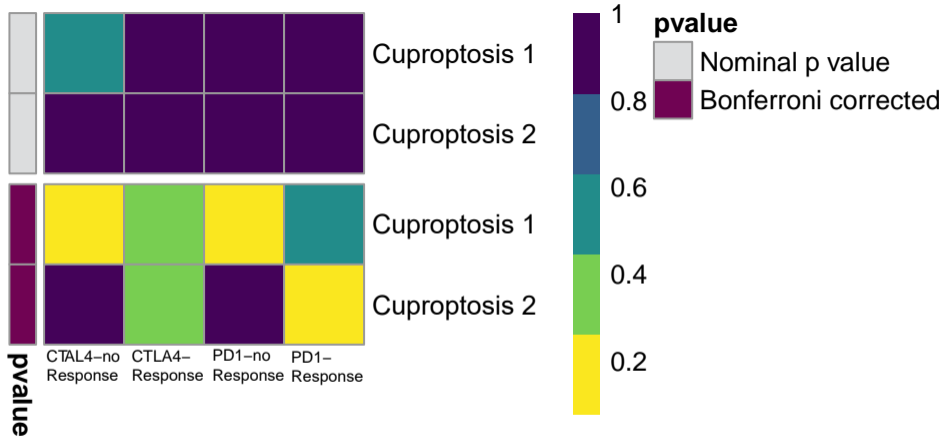

Supplement: Supplementary file 5 [file DataSheet_5.zip › Step5/map/1.pdf]

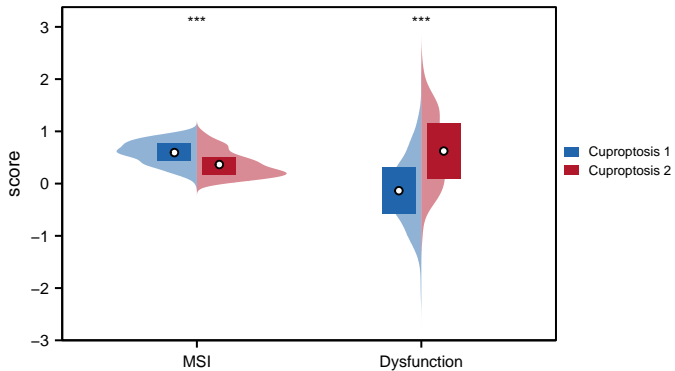

Supplement: Supplementary file 5 [file DataSheet_5.zip › Step5/map/╢╣╝╘═╝_2022-03-28_13_11_55.pdf]

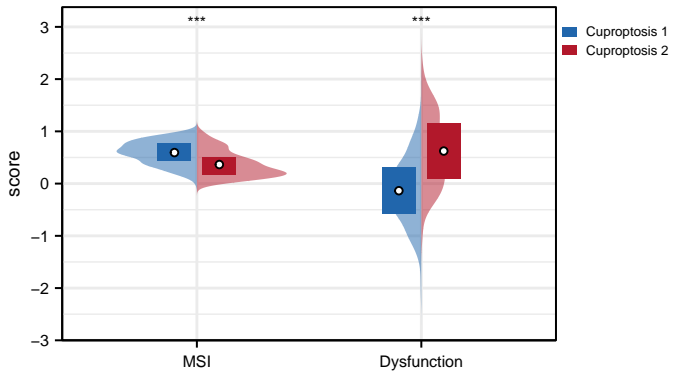

Supplement: Supplementary file 5 [file DataSheet_5.zip › Step5/map/╢╣╝╘═╝_2022-03-28_14_58_51.pdf]

TIDE score

\*\*

Cuproptosis 1

Cuproptosis 2

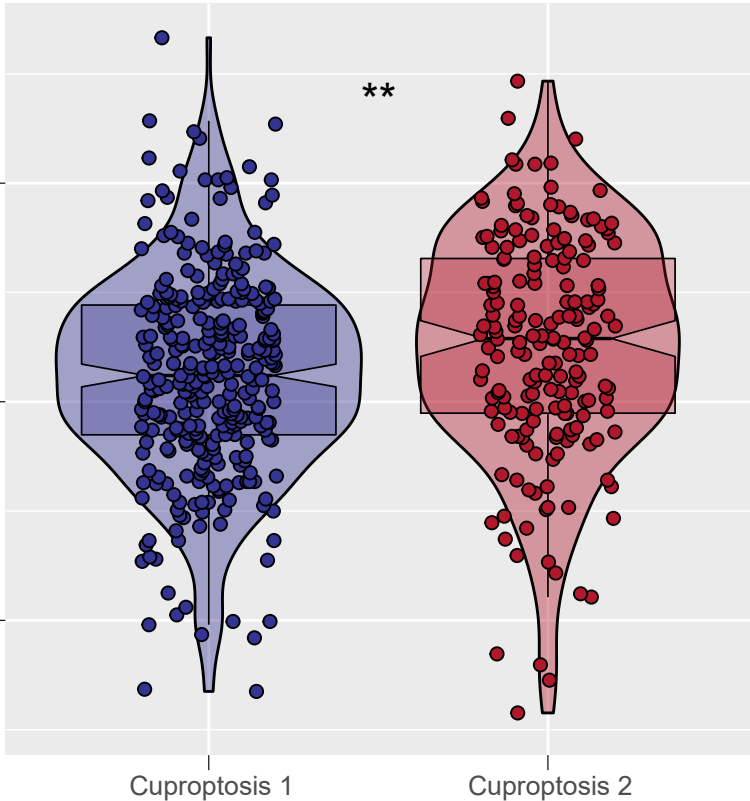

Supplement: Supplementary file 5 [file DataSheet_5.zip › Step5/tide/3.pdf]

MSI score

1.00  
0.75  
0.50  
0.25  
0.00

Cuproptosis 1

\*\*\*

Cuproptosis 2

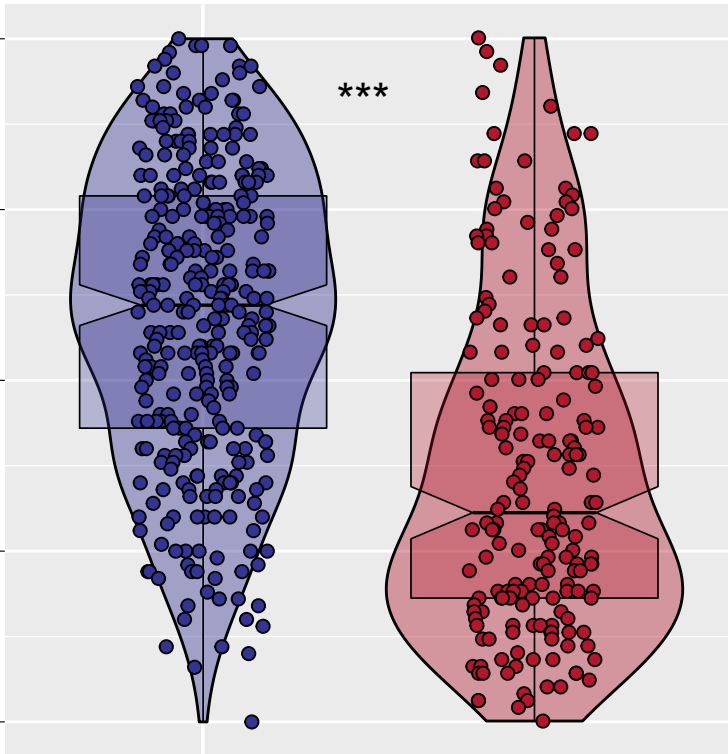

Supplement: Supplementary file 5 [file DataSheet_5.zip › Step5/tide/boxViolin.pdf]

TIDE

Responder

No\_Responder

-2.5

0.0

2.5

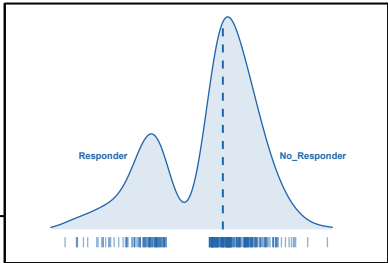

Supplement: Supplementary file 5 [file DataSheet_5.zip › Step5/tide/╔╜┬══╝_2022-03-28_12_23_18.pdf]

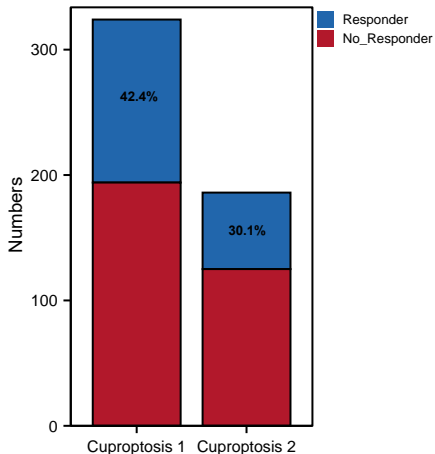

Supplement: Supplementary file 5 [file DataSheet_5.zip › Step5/tide/╓∙╫┤═╝_2022-03-28_12_24_44.pdf]

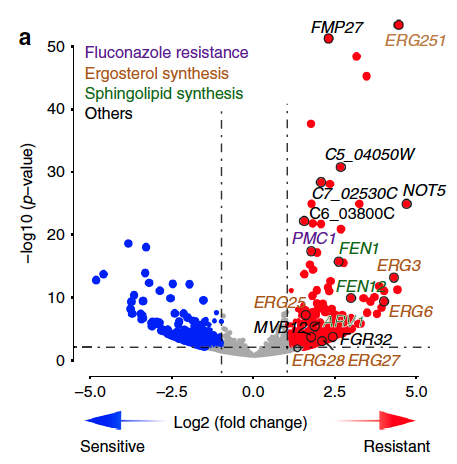

Supplement: Supplementary file 5 [file DataSheet_5.zip › Step5/vol/FigureYa59volcanoV2/example.png]

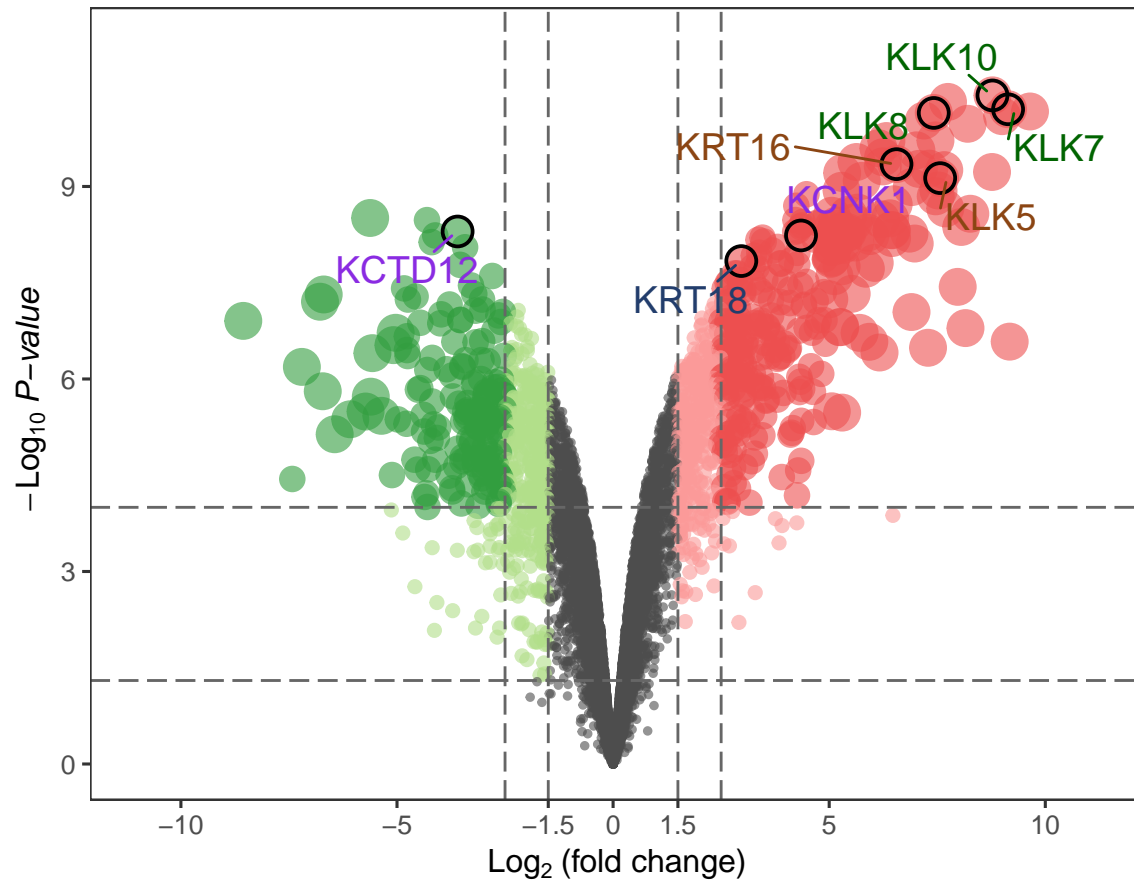

Supplement: Supplementary file 5 [file DataSheet_5.zip › Step5/vol/FigureYa59volcanoV2/Volcano_advanced.pdf]

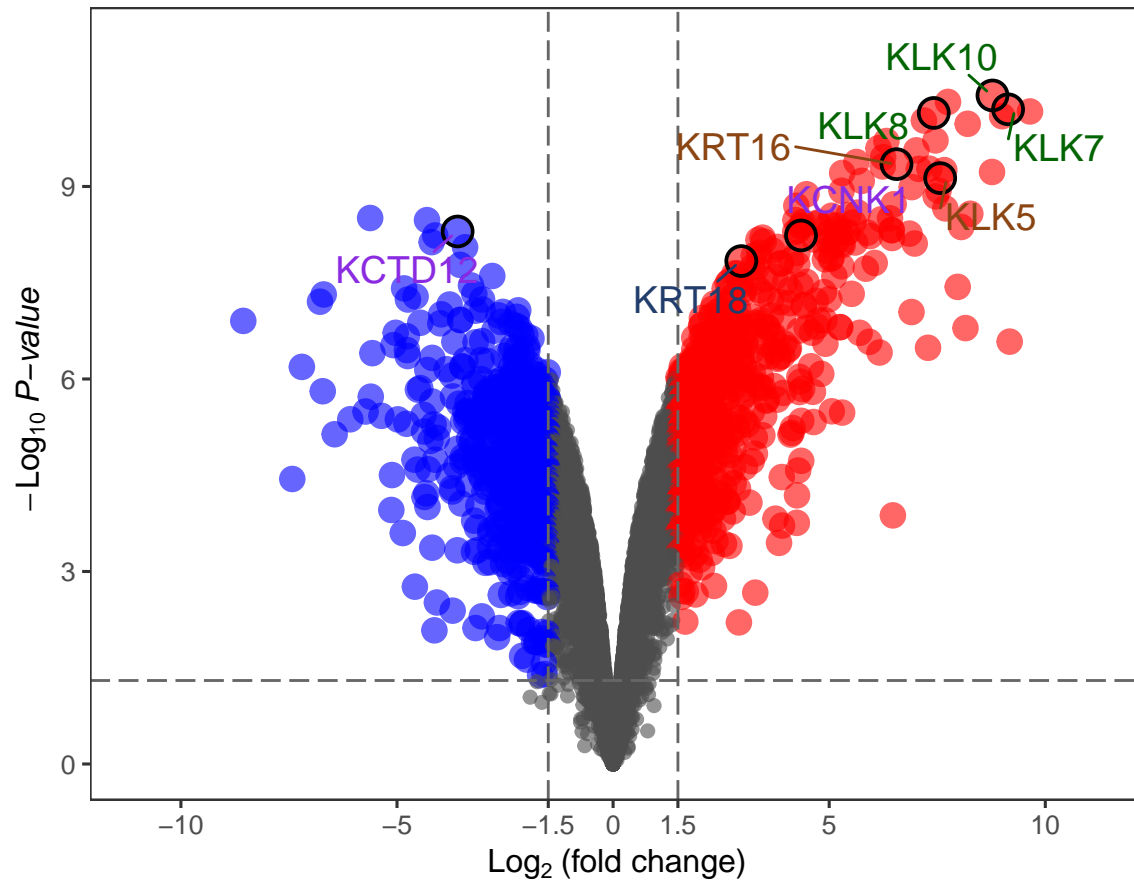

Supplement: Supplementary file 5 [file DataSheet_5.zip › Step5/vol/FigureYa59volcanoV2/volcano_classic.pdf]

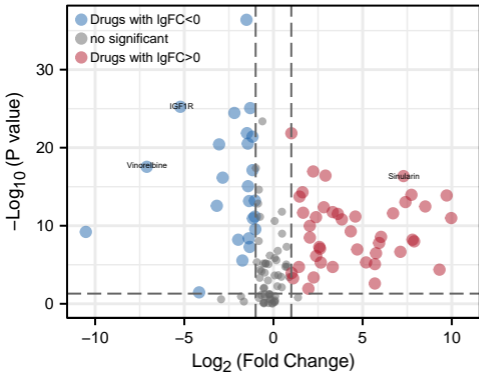

Supplement: Supplementary file 5 [file DataSheet_5.zip › Step5/vol/╗≡╔╜═╝_2022-03-29_17_10_27.pdf]

Drugs in  
GDSC dataset

Drugs in  
CTRP and PRISM datasets

110

26

328

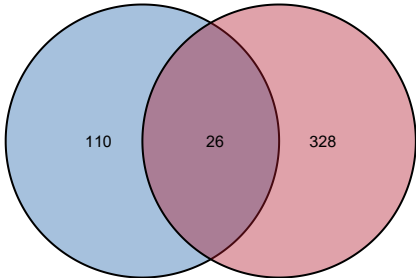

Supplement: Supplementary file 5 [file DataSheet_5.zip › Step5/vol/╬1⁄4╢≈═╝_2022-03-29_23_21_08.pdf]

Estimated IC50

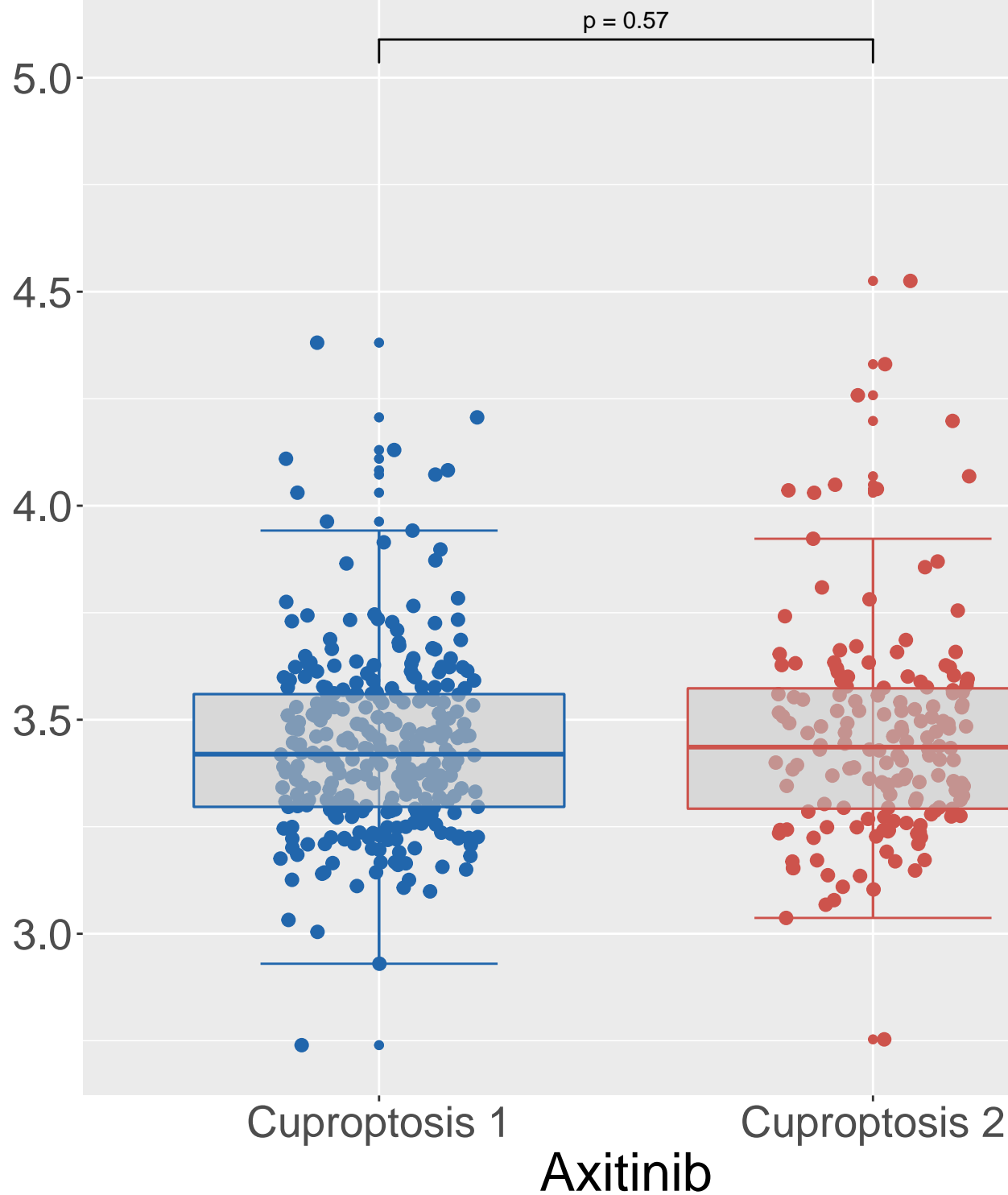

Supplement: Supplementary file 5 [file DataSheet_5.zip › Step5/yao/Axitinib.pdf]

Estimated IC50

$p = 0.58$

Cuproptosis 1

Cuproptosis 2

Bosutinib

5  
4  
3  
2

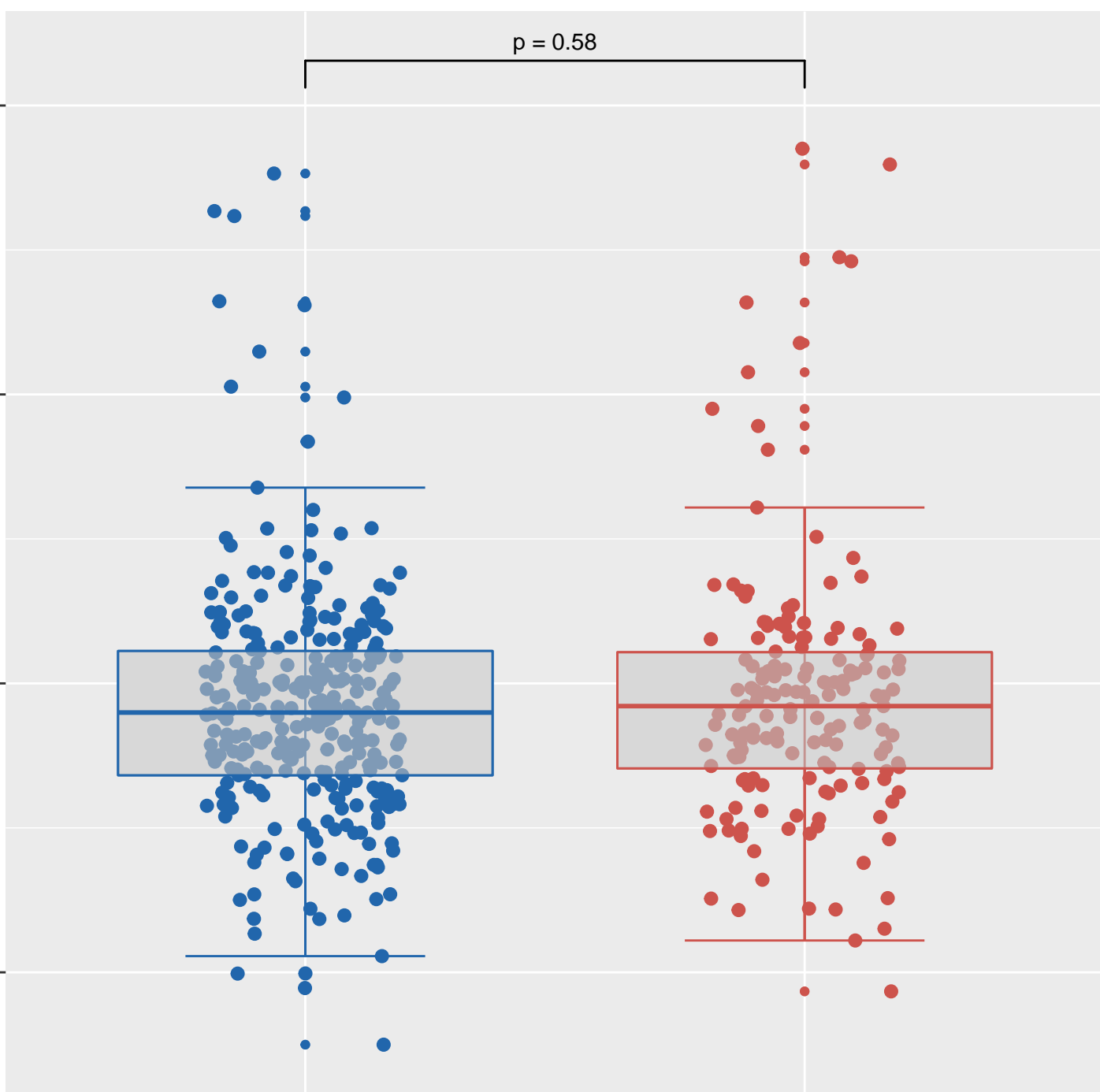

Supplement: Supplementary file 5 [file DataSheet_5.zip › Step5/yao/Bosutinib.pdf]

Estimated IC50

2000

1000

0

Cuproptosis 1

Cuproptosis 2

Linsitinib\_1510

$p = 4.1e-37$

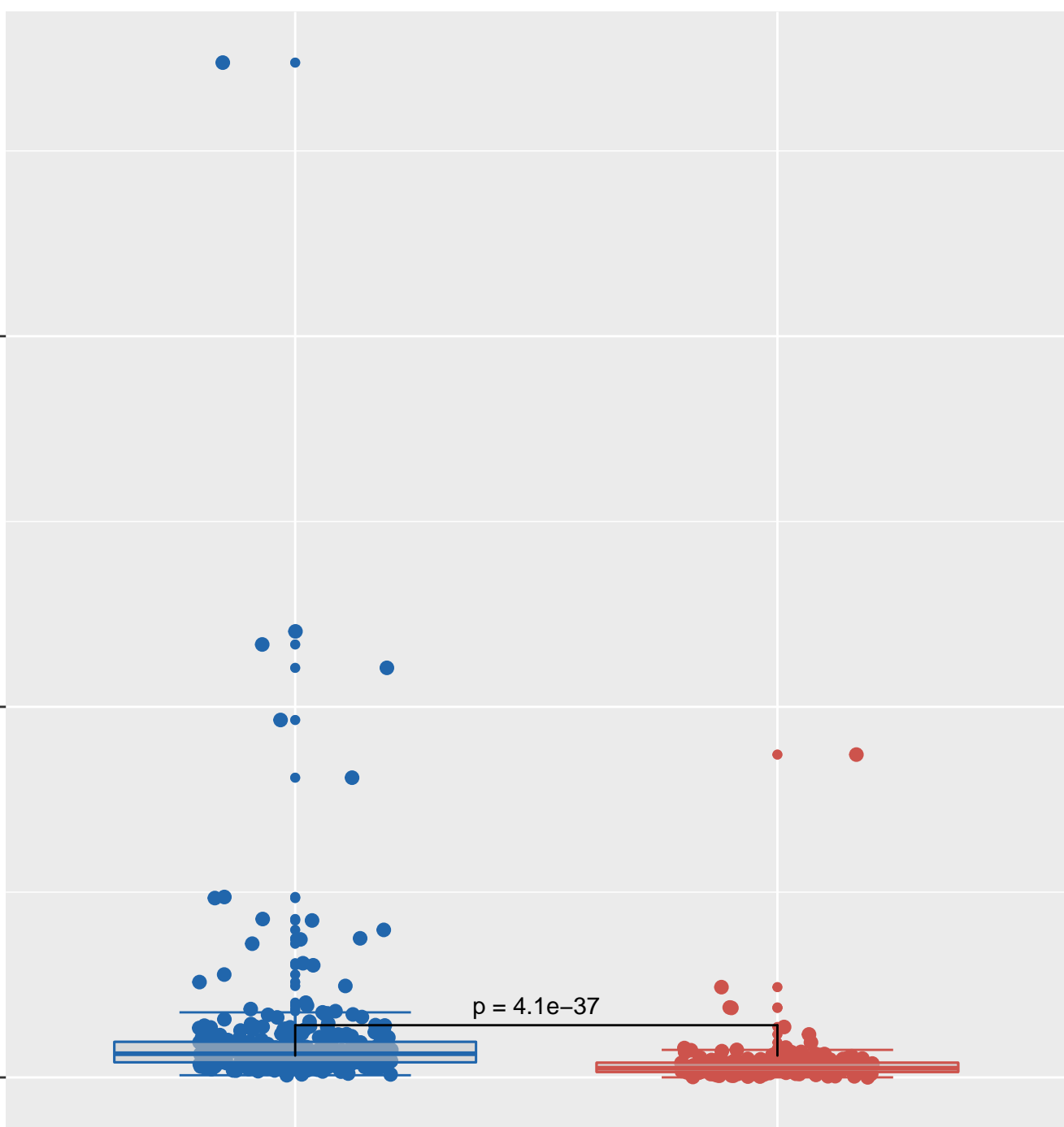

Supplement: Supplementary file 5 [file DataSheet_5.zip › Step5/yao/calcPhenotype_Output/Linsitinib_1510.pdf]

Estimated IC50

$p = 0.65$

4  
3  
2  
1

Cuproptosis 1

Cuproptosis 2

Gefitinib

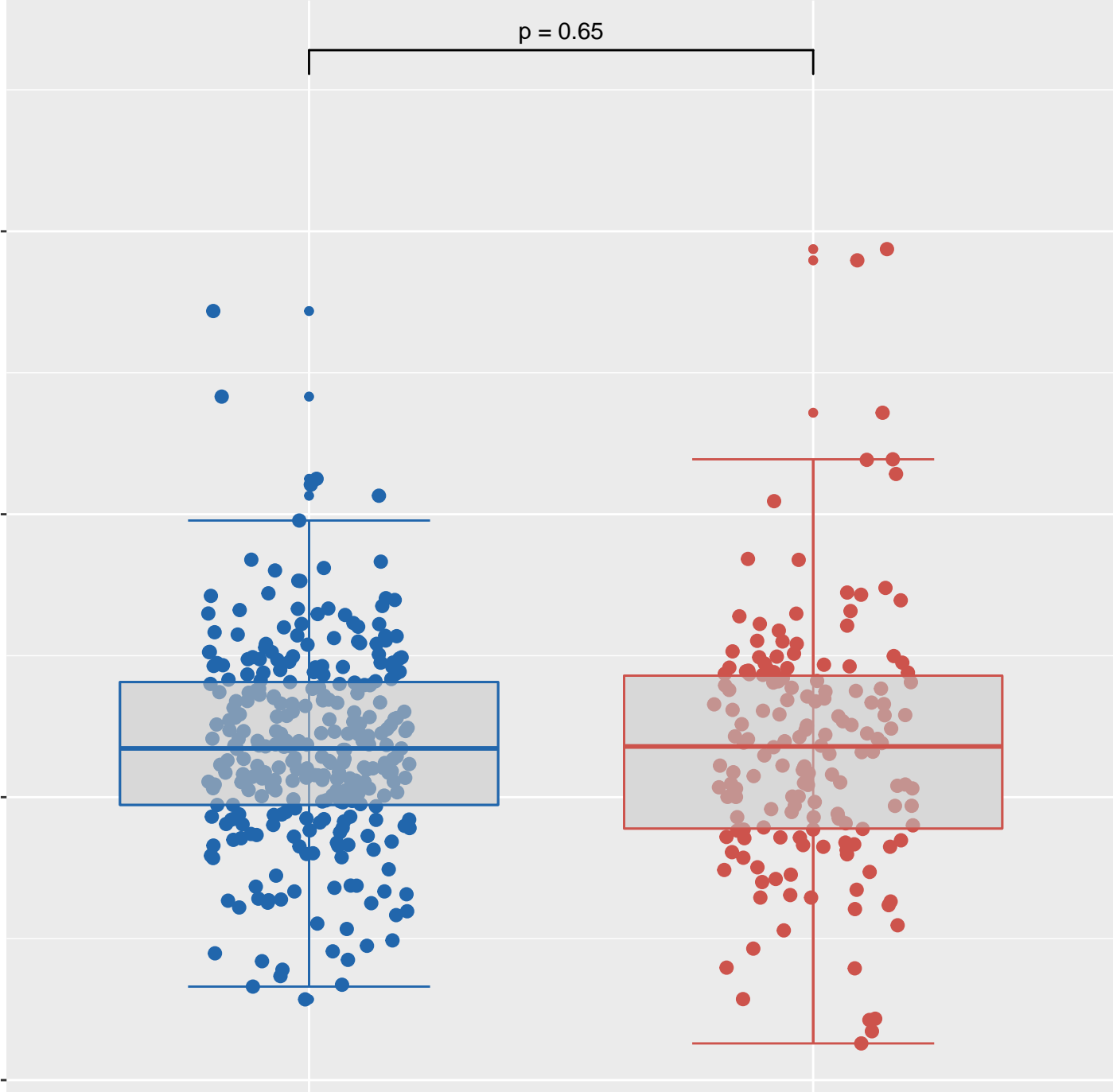

Supplement: Supplementary file 5 [file DataSheet_5.zip › Step5/yao/Gefitinib.pdf]

Estimated IC50

$p = 0.32$

5

4

3

Cuproptosis 1

Cuproptosis 2

Lapatinib

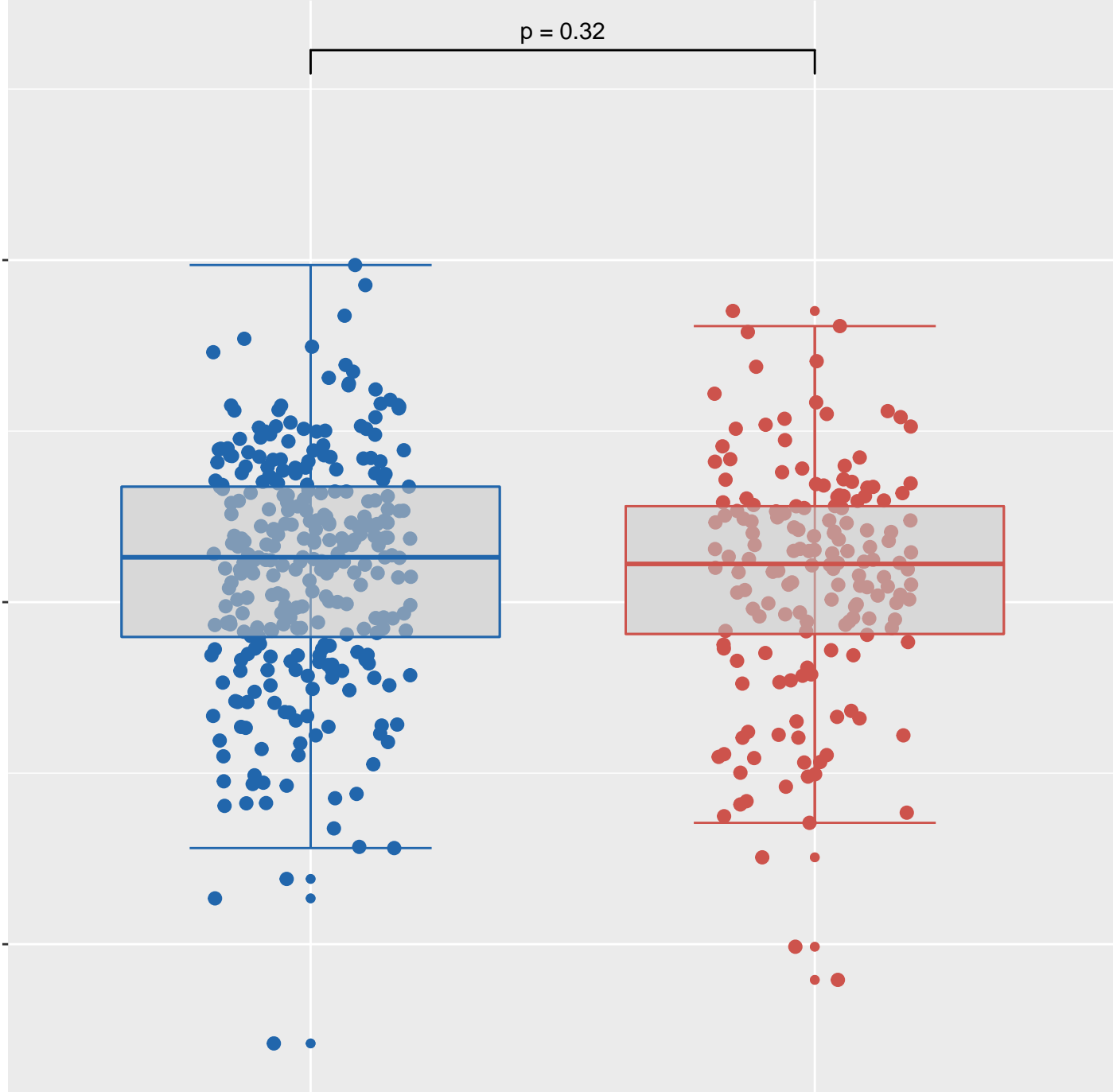

Supplement: Supplementary file 5 [file DataSheet_5.zip › Step5/yao/Lapatinib.pdf]

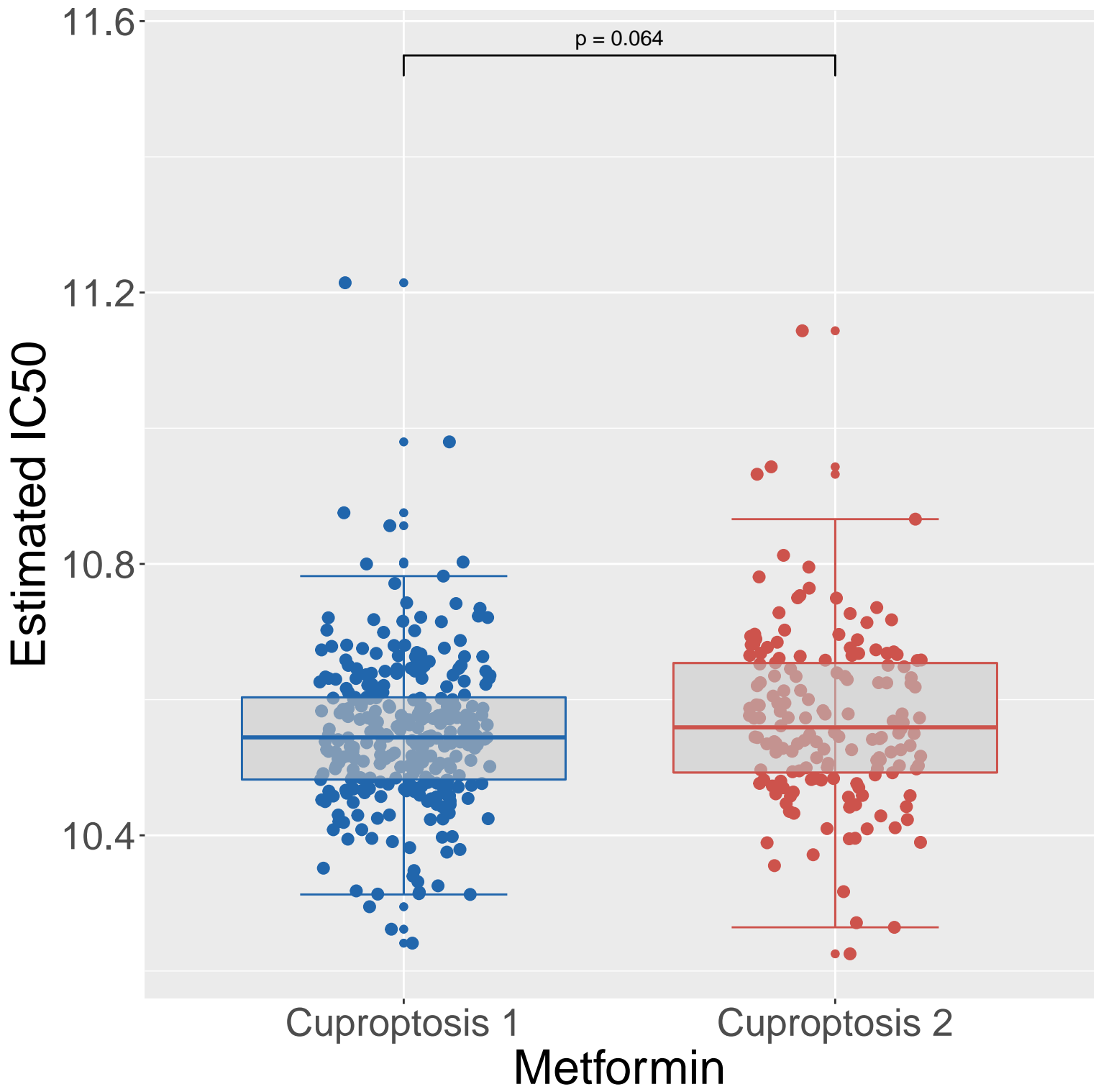

Supplement: Supplementary file 5 [file DataSheet_5.zip › Step5/yao/Metformin.pdf]

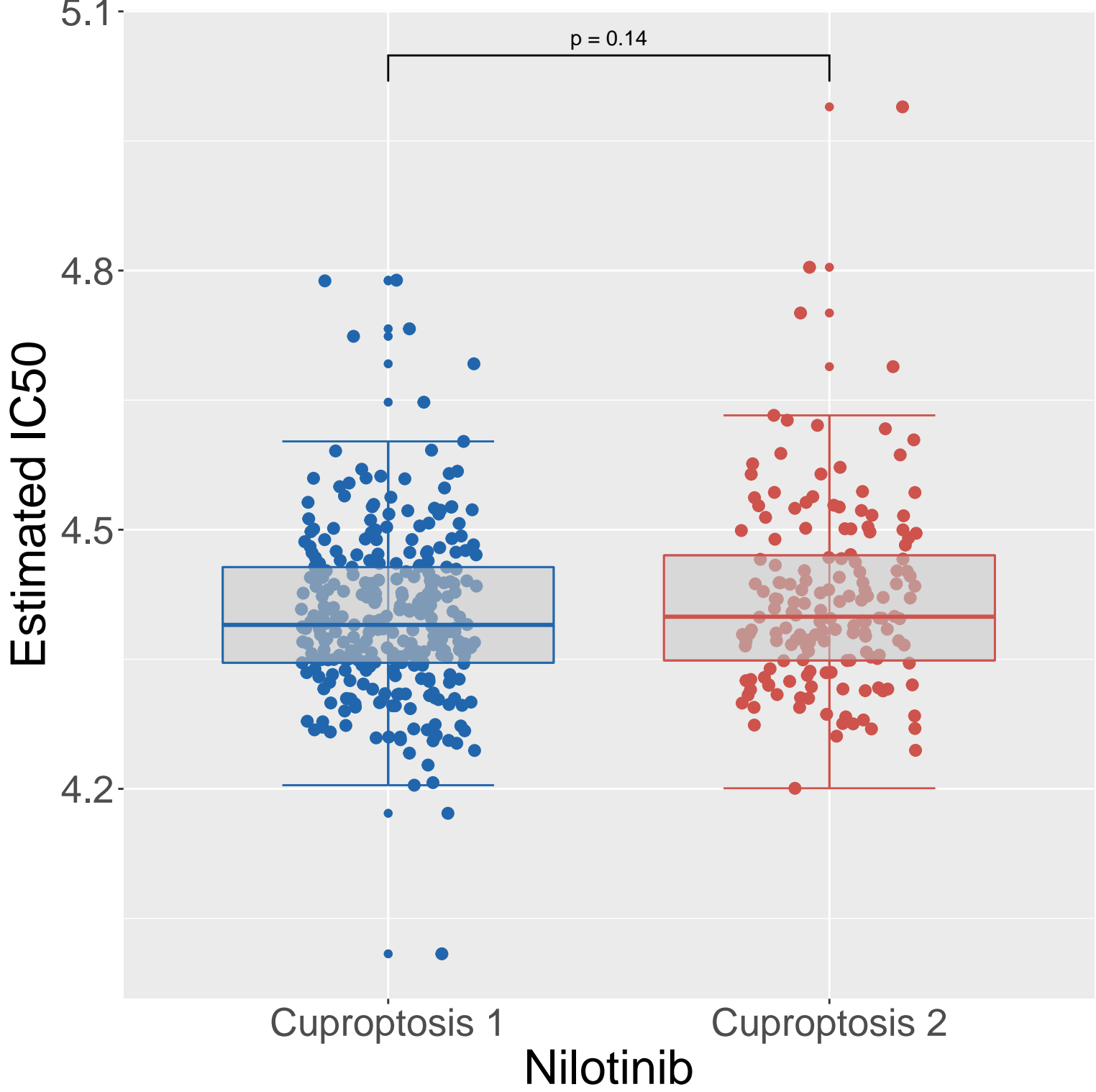

Supplement: Supplementary file 5 [file DataSheet_5.zip › Step5/yao/Nilotinib.pdf]

Estimated IC50

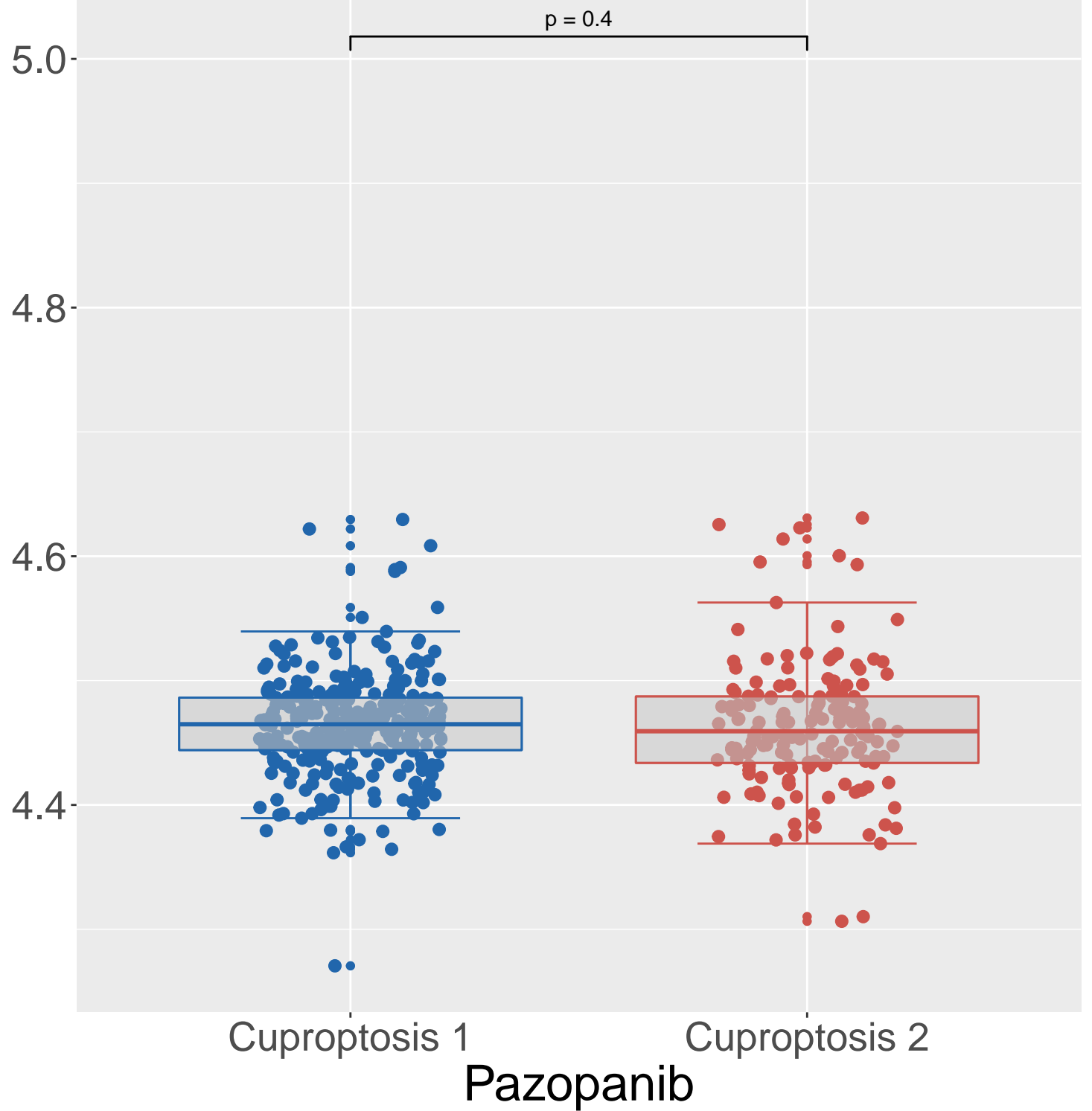

Supplement: Supplementary file 5 [file DataSheet_5.zip › Step5/yao/Pazopanib.pdf]

Estimated IC50

$p = 0.21$

4.4

4.2

4.0

3.8

3.6

Cuproptosis 1

Cuproptosis 2

Sorafenib

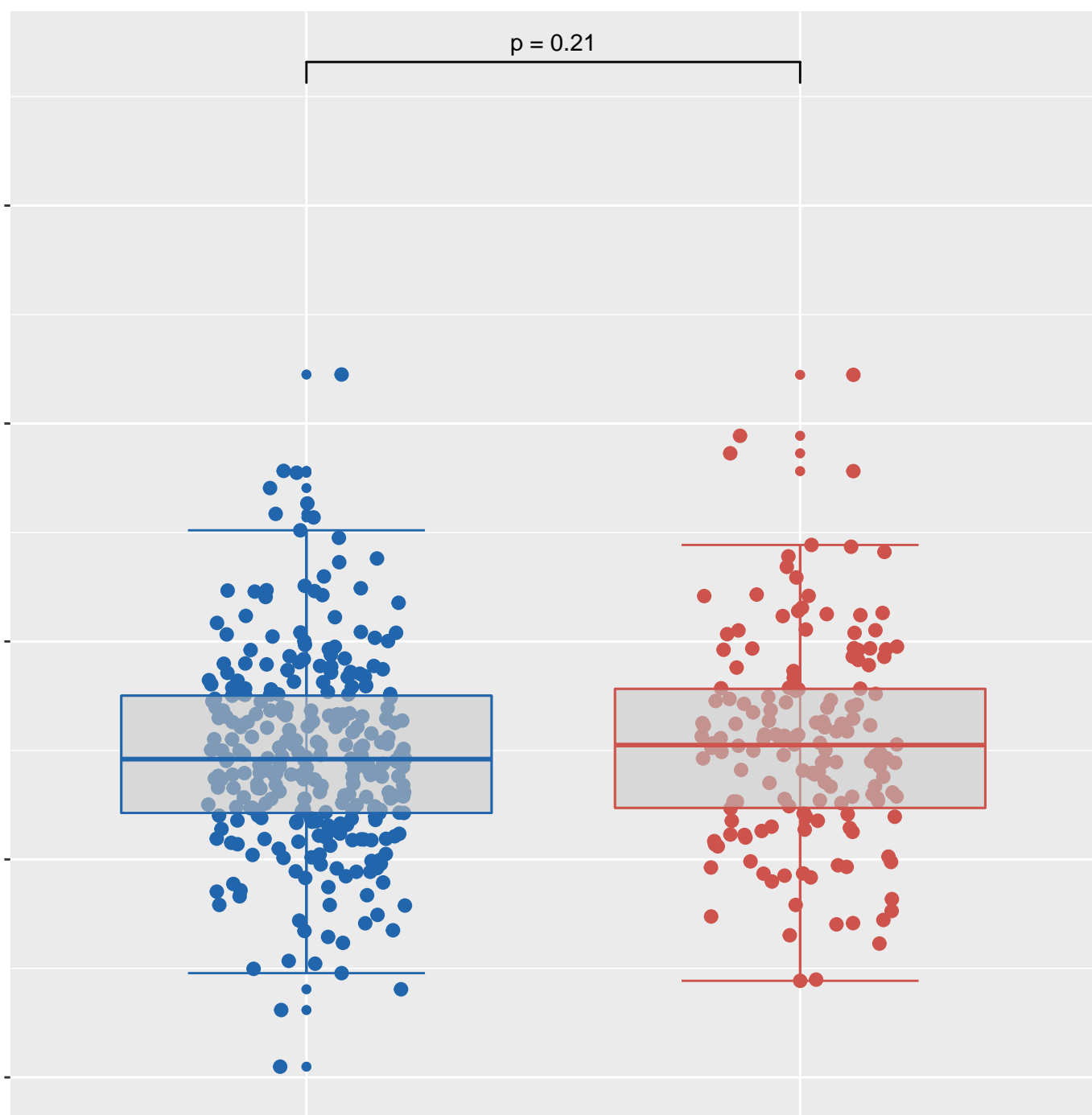

Supplement: Supplementary file 5 [file DataSheet_5.zip › Step5/yao/Sorafenib.pdf]

Estimated IC50

$p = 0.11$

4.5

4.2

3.9

3.6

Cuproptosis 1

Cuproptosis 2

Sunitinib

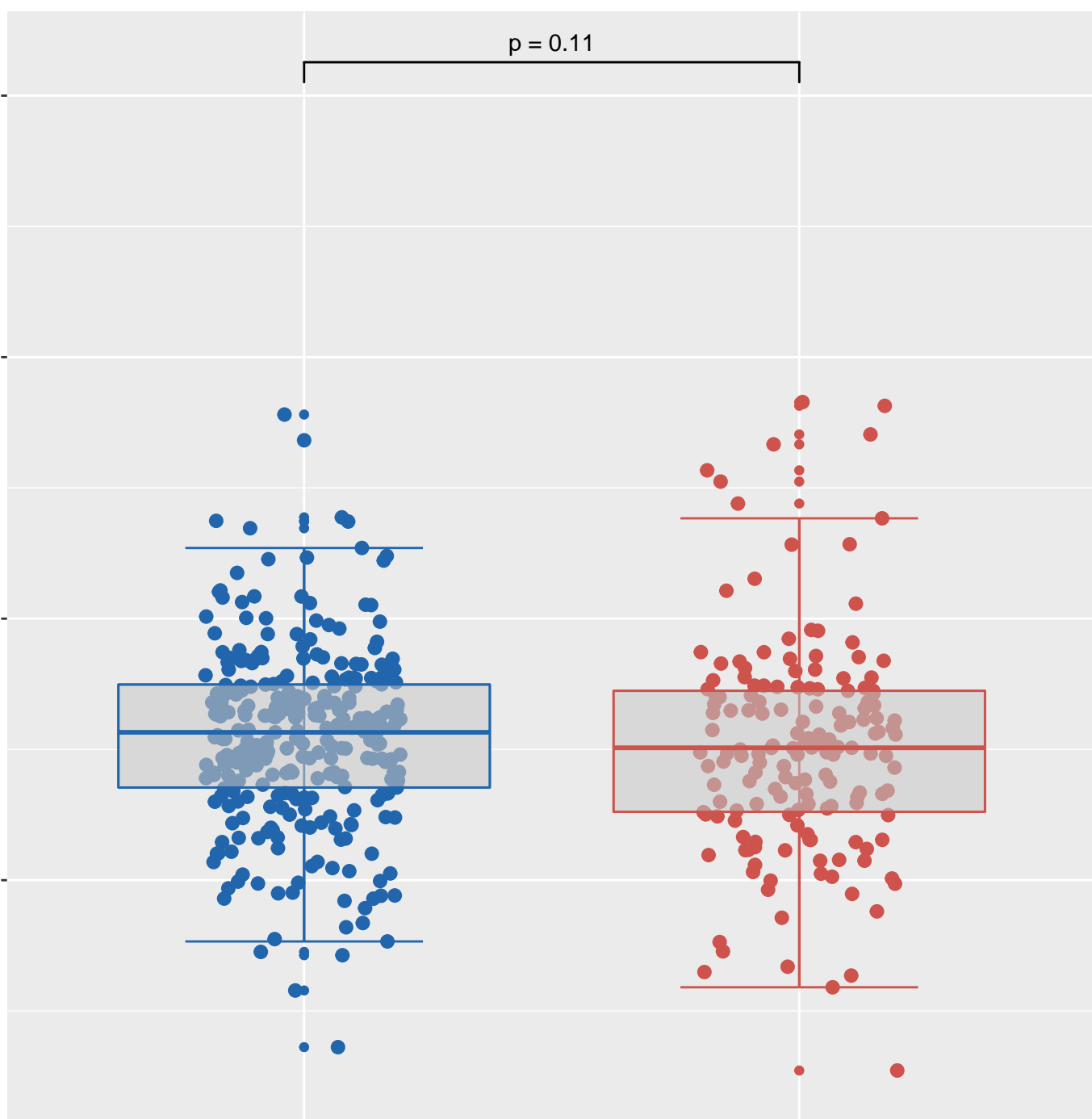

Supplement: Supplementary file 5 [file DataSheet_5.zip › Step5/yao/Sunitinib.pdf]

Estimated IC50

$p = 0.44$

Cuproptosis 1

Cuproptosis 2

Temsirolimus

2  
1  
0  
-1  
-2

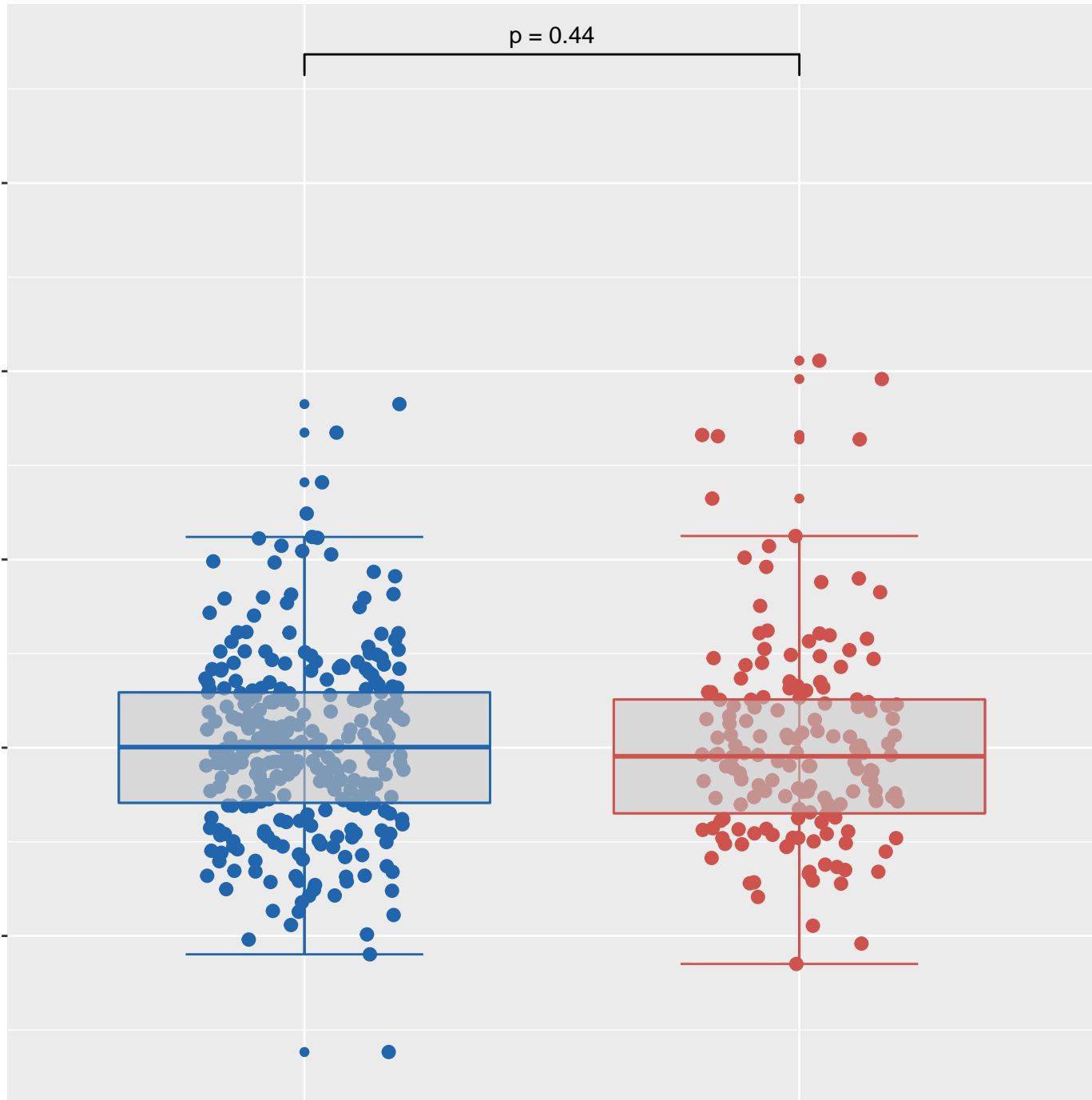

Supplement: Supplementary file 5 [file DataSheet_5.zip › Step5/yao/Temsirolimus.pdf]

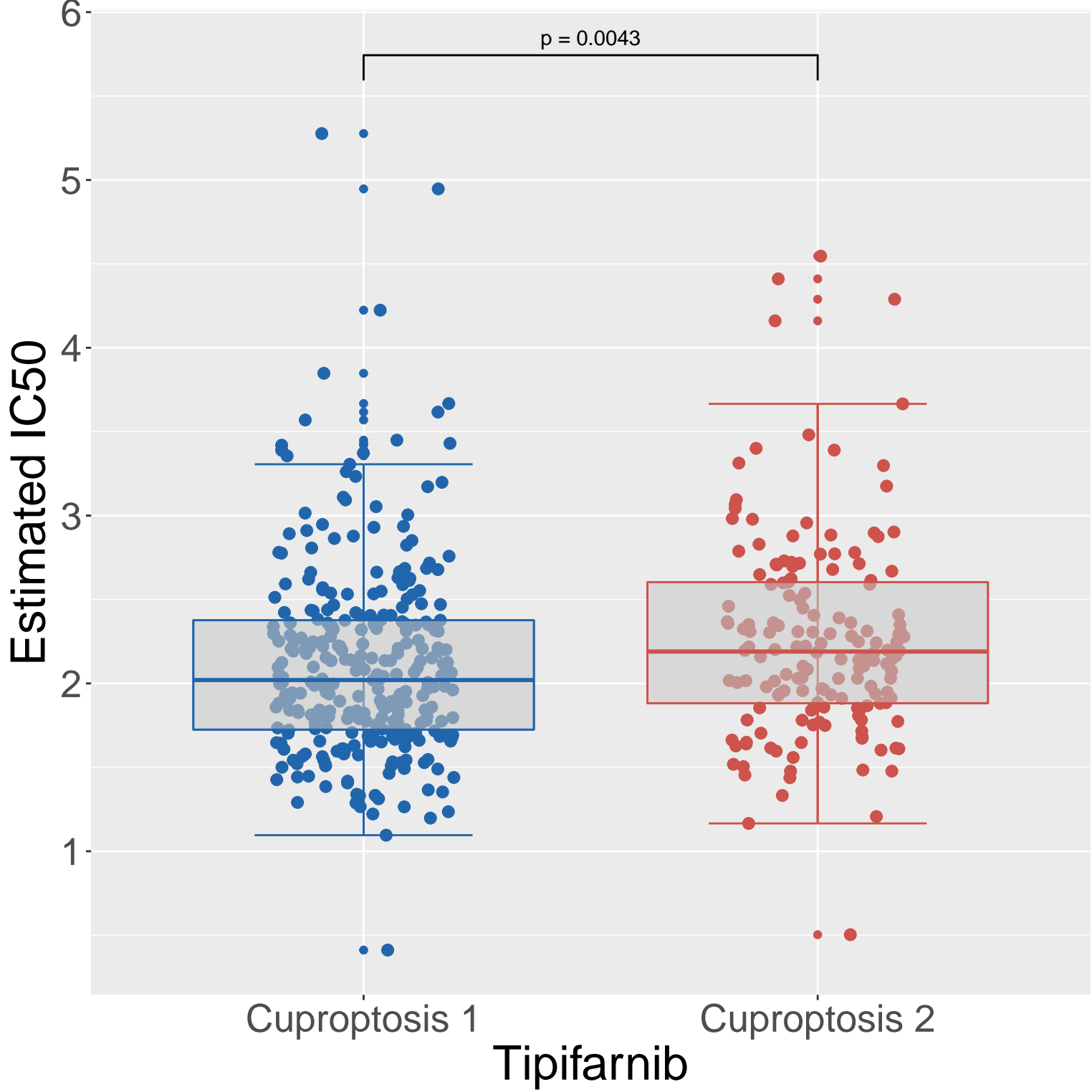

Supplement: Supplementary file 5 [file DataSheet_5.zip › Step5/yao/Tipifarnib.pdf]

Estimated IC50

$p = 0.15$

Cuproptosis 1

Cuproptosis 2

Vorinostat

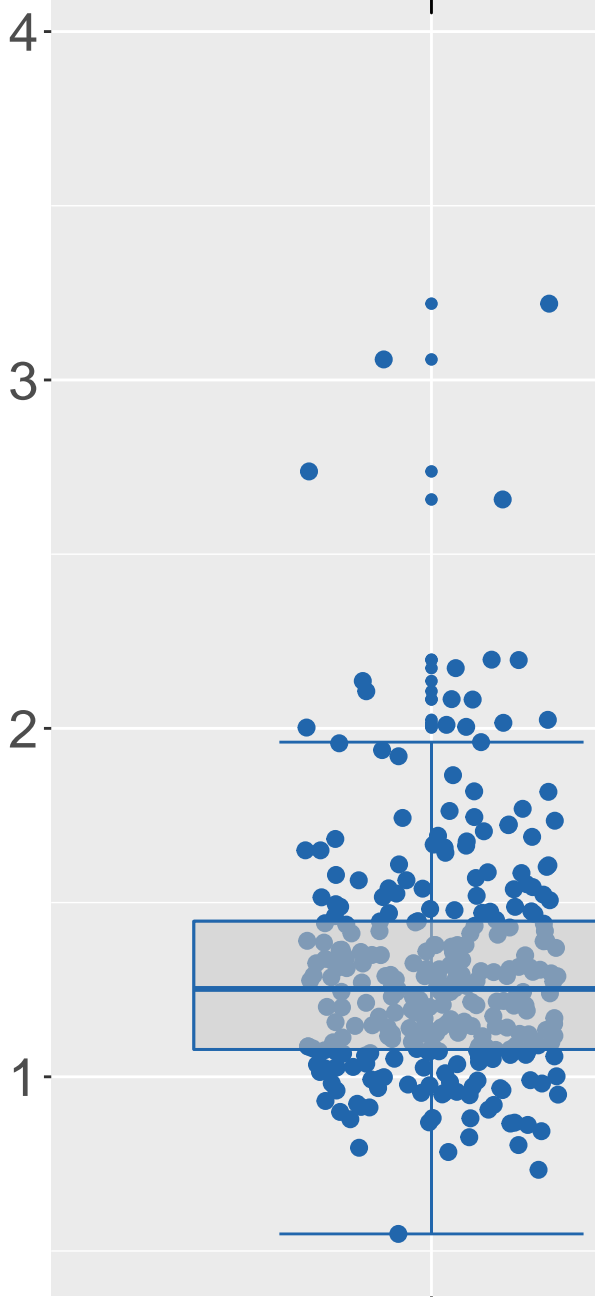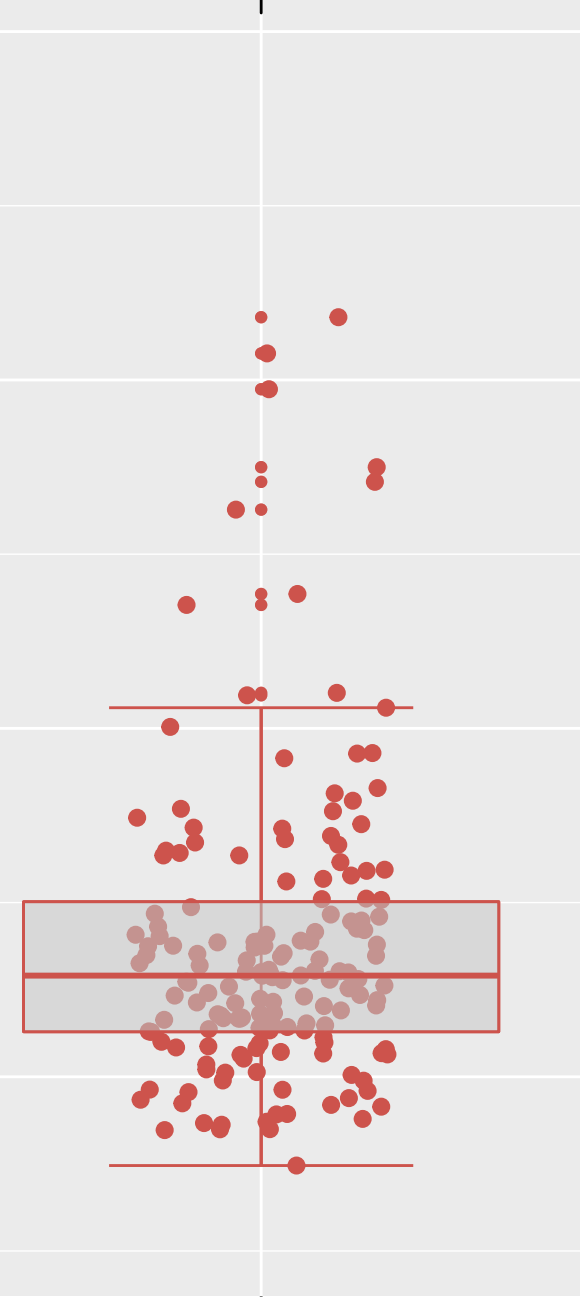

Supplement: Supplementary file 5 [file DataSheet_5.zip › Step5/yao/Vorinostat.pdf]

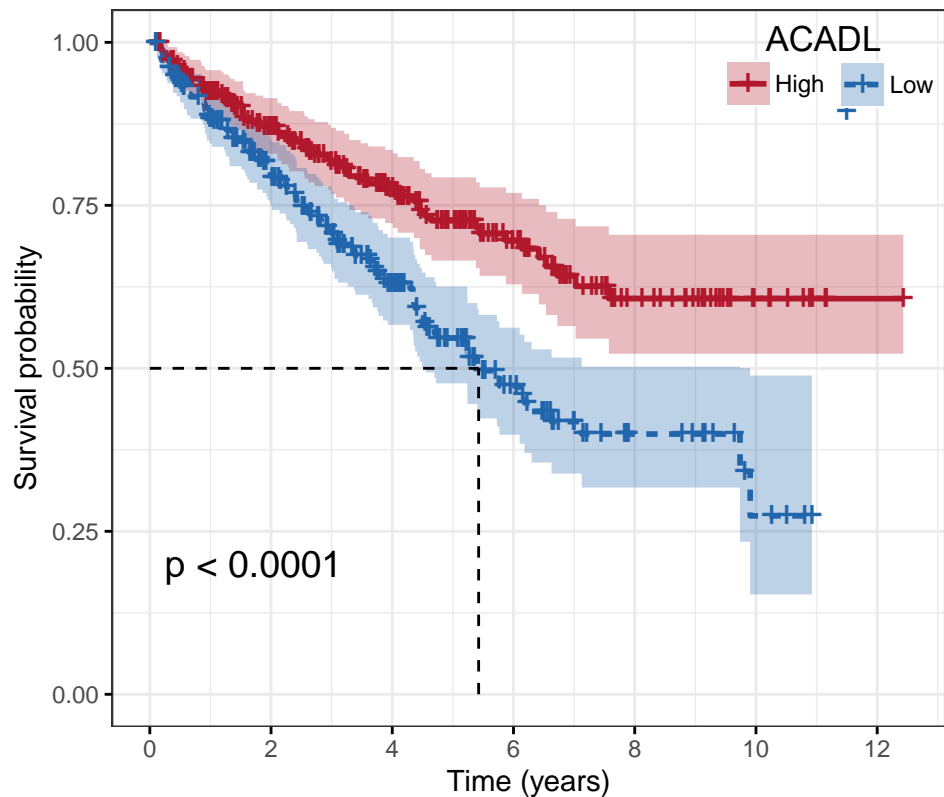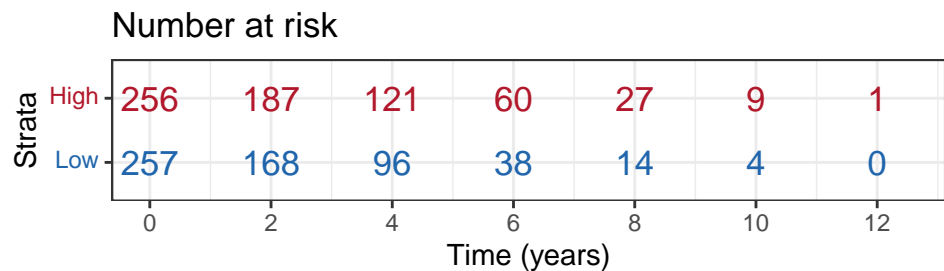

Supplement: Supplementary file 6 [file DataSheet_6.zip › Step6/sur/ACADL.pdf]

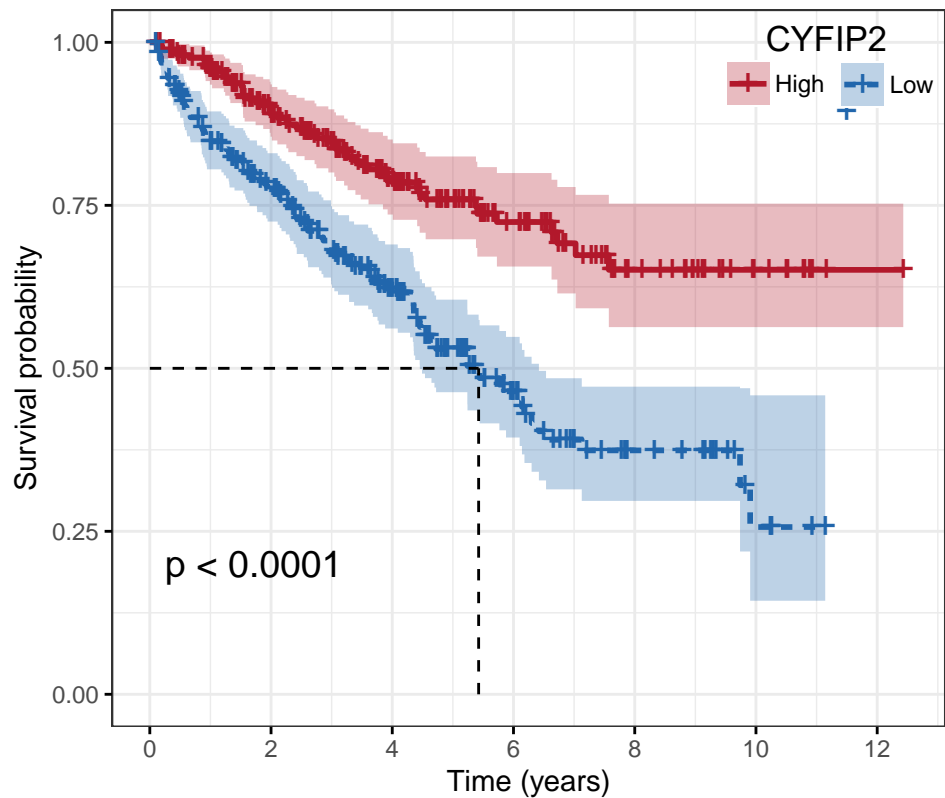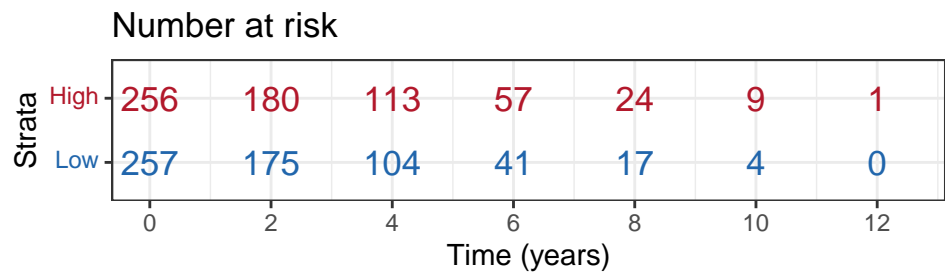

Supplement: Supplementary file 6 [file DataSheet_6.zip › Step6/sur/CYFIP2.pdf]

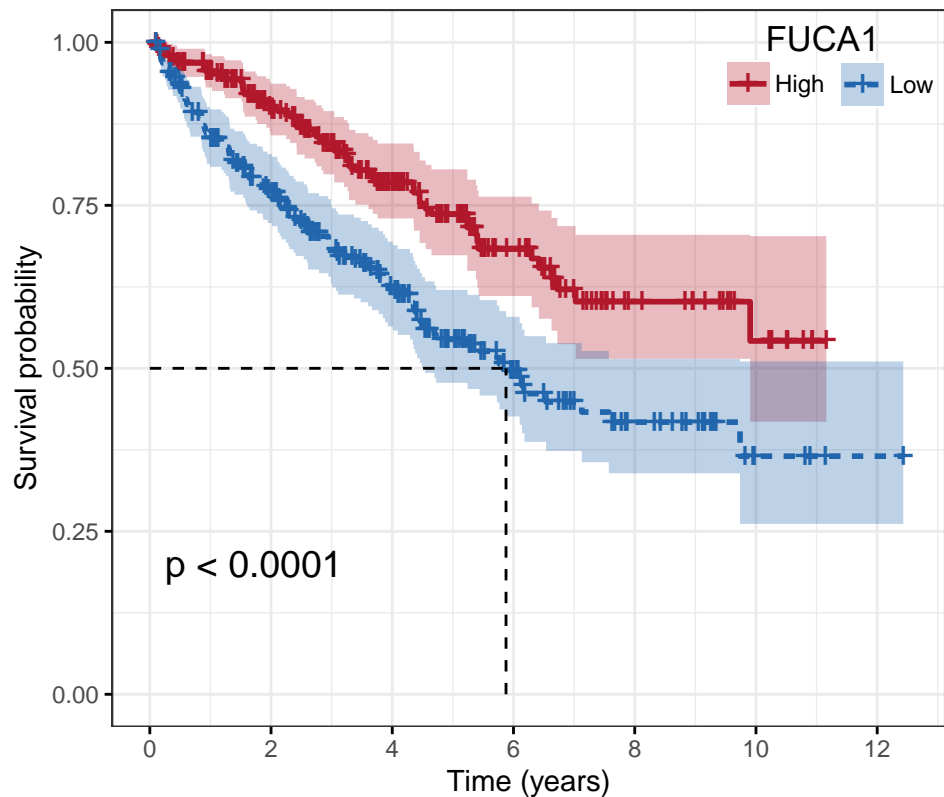

Number at risk

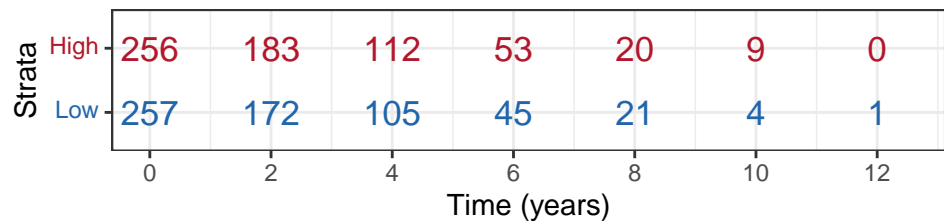

Supplement: Supplementary file 6 [file DataSheet_6.zip › Step6/sur/FUCA1.pdf]

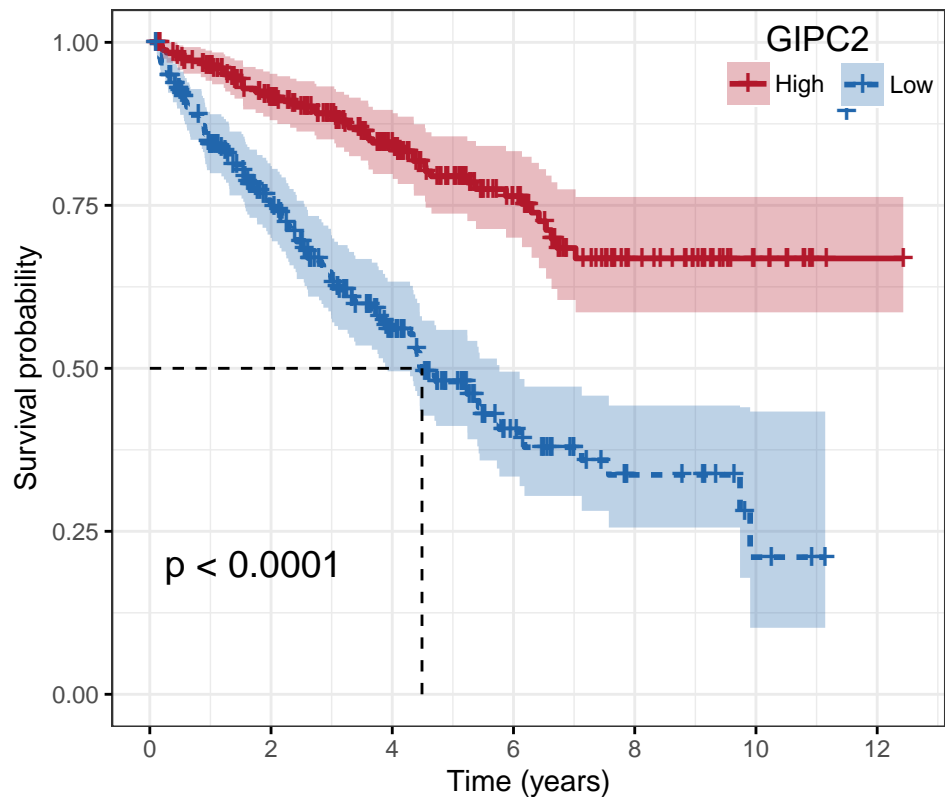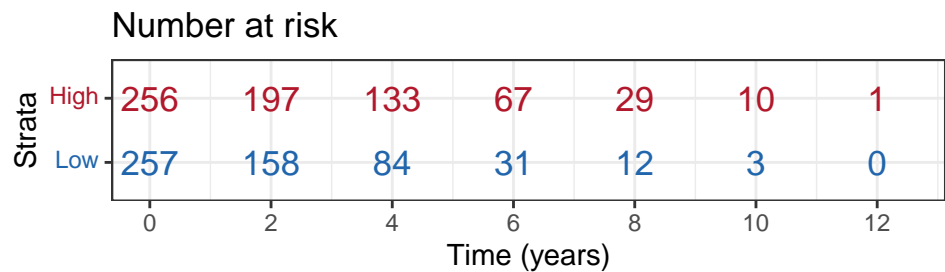

Supplement: Supplementary file 6 [file DataSheet_6.zip › Step6/sur/GIPC2.pdf]

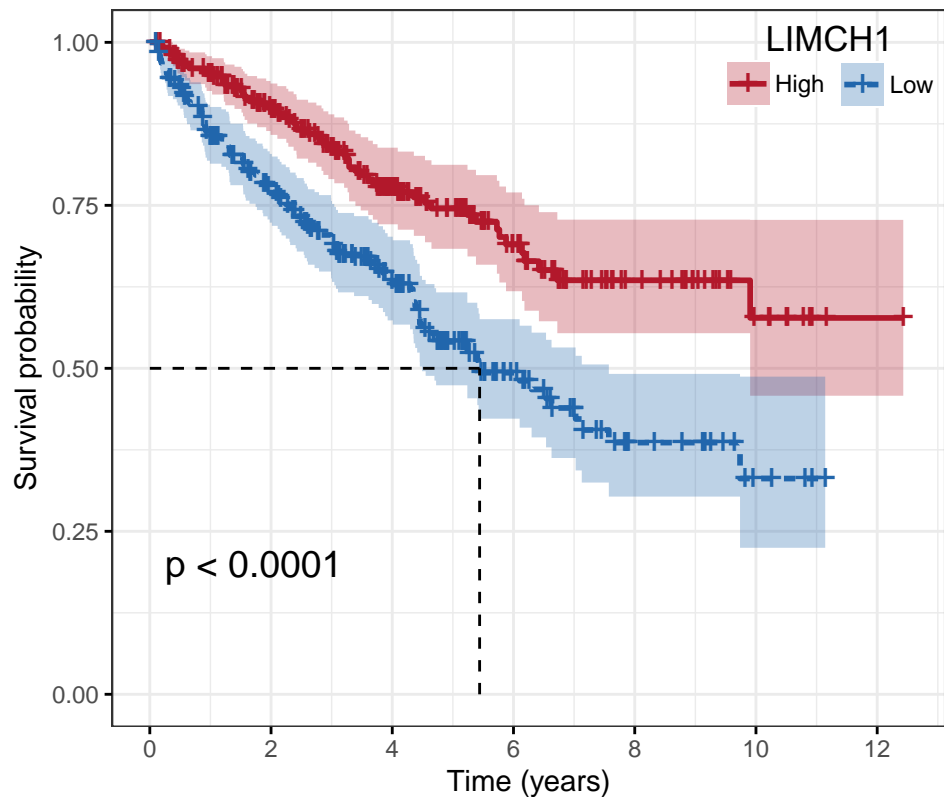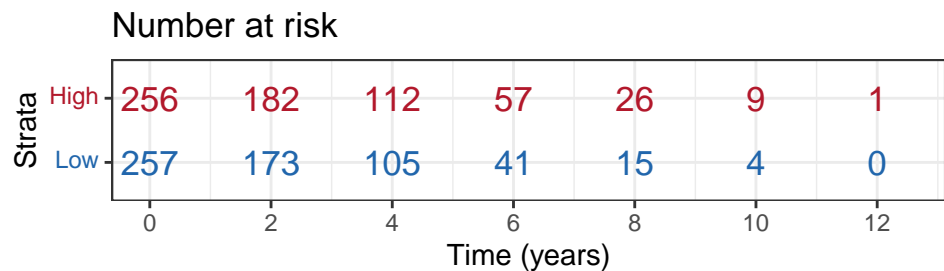

Supplement: Supplementary file 6 [file DataSheet_6.zip › Step6/sur/LIMCH1.pdf]

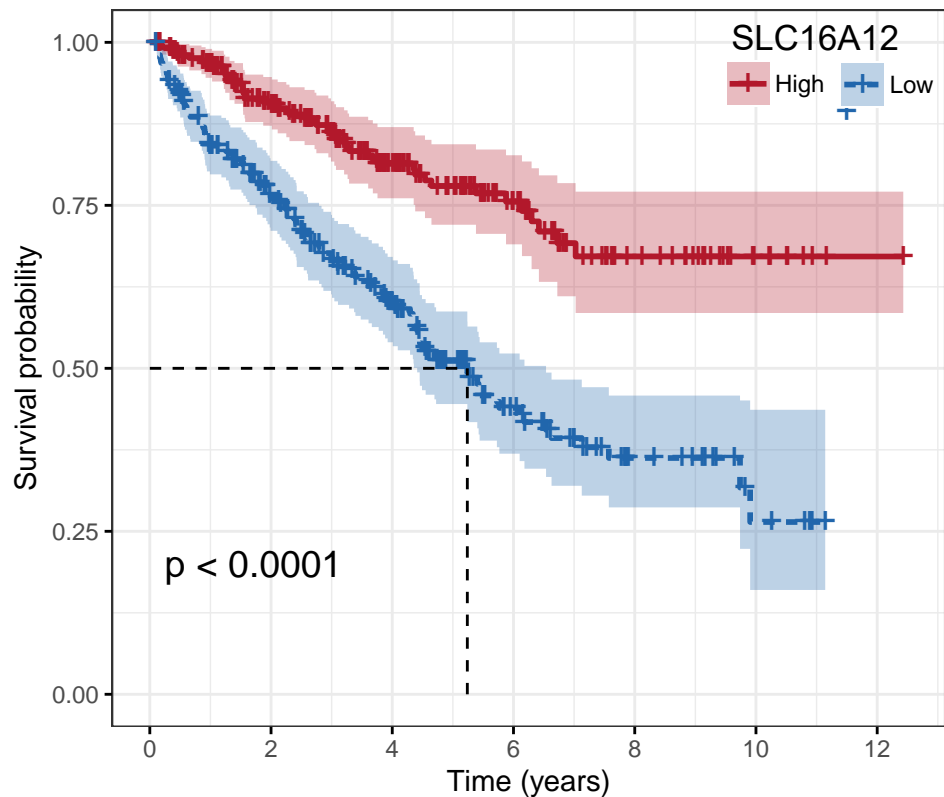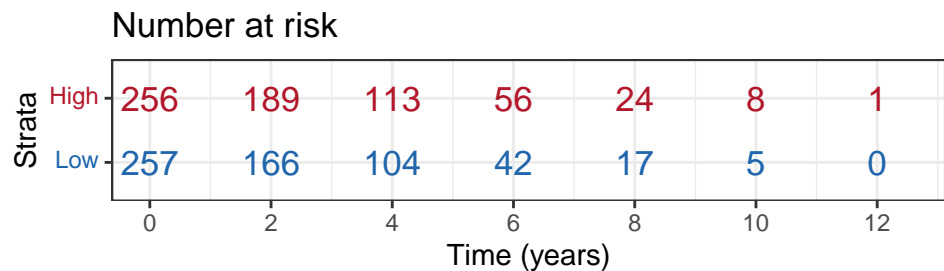

Supplement: Supplementary file 6 [file DataSheet_6.zip › Step6/sur/SLC16A12.pdf]

# Module-trait relationships

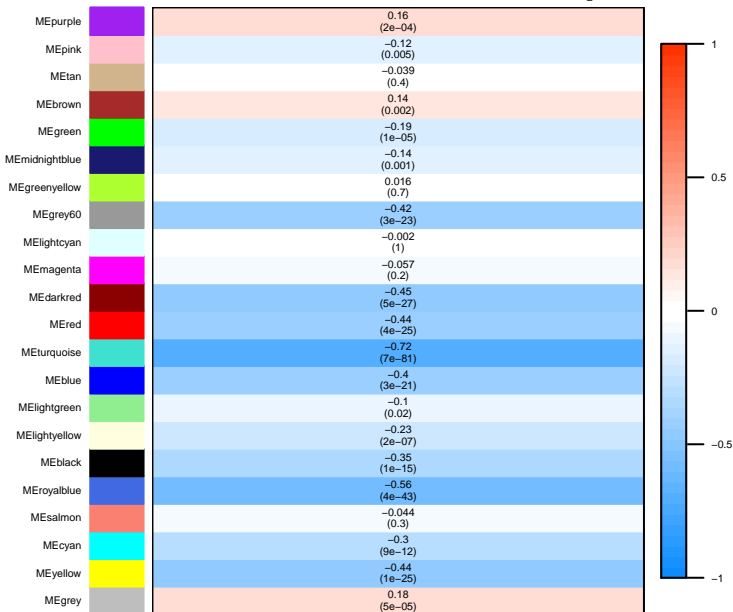

Cuproptosis cluster

Supplement: Supplementary file 6 [file DataSheet_6.zip › Step6/wgcna/4.3.pdf]

# Module-trait relationships

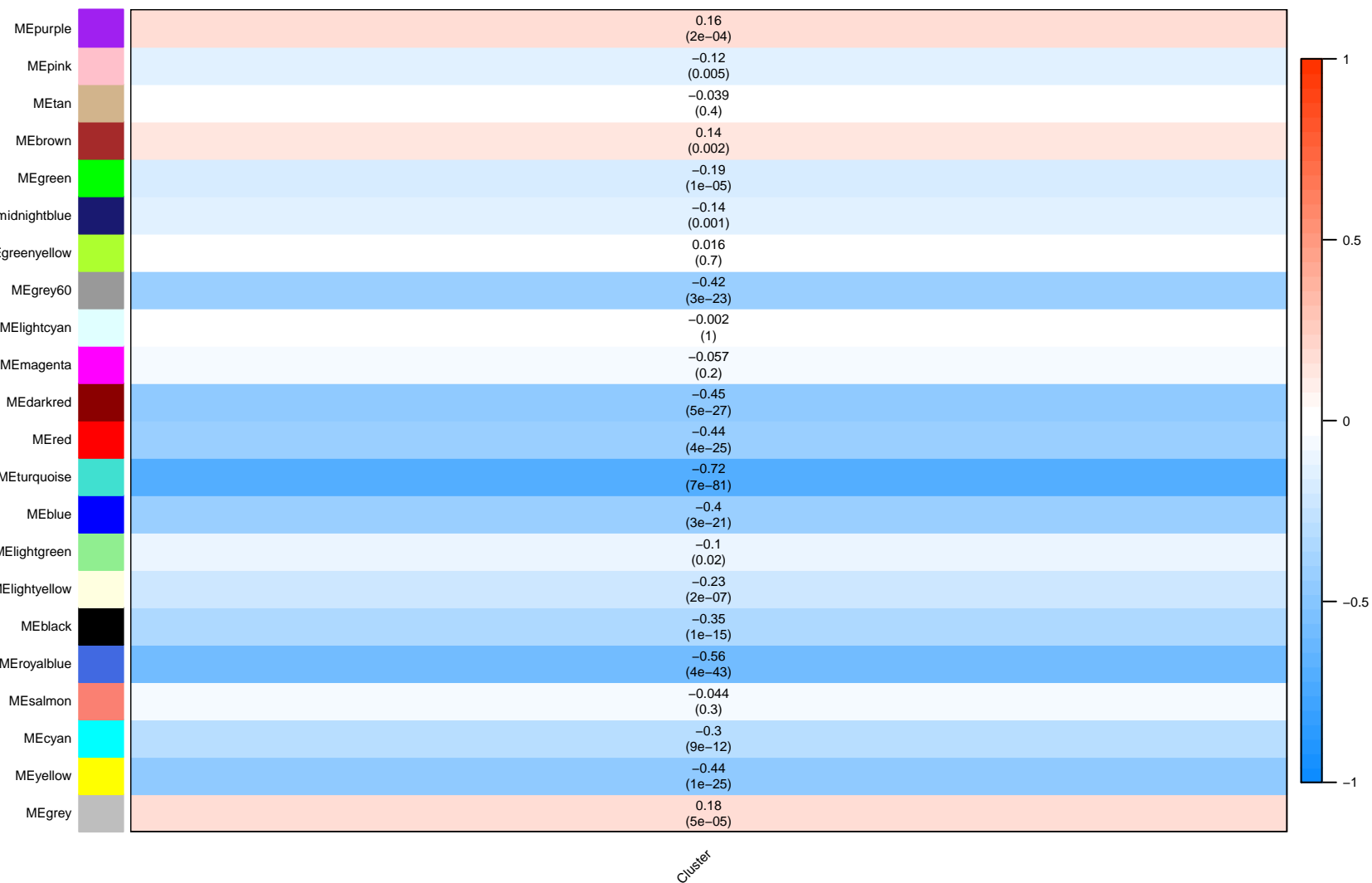

Supplement: Supplementary file 6 [file DataSheet_6.zip › Step6/wgcna/Module.pdf]

Gene dendrogram and module colors

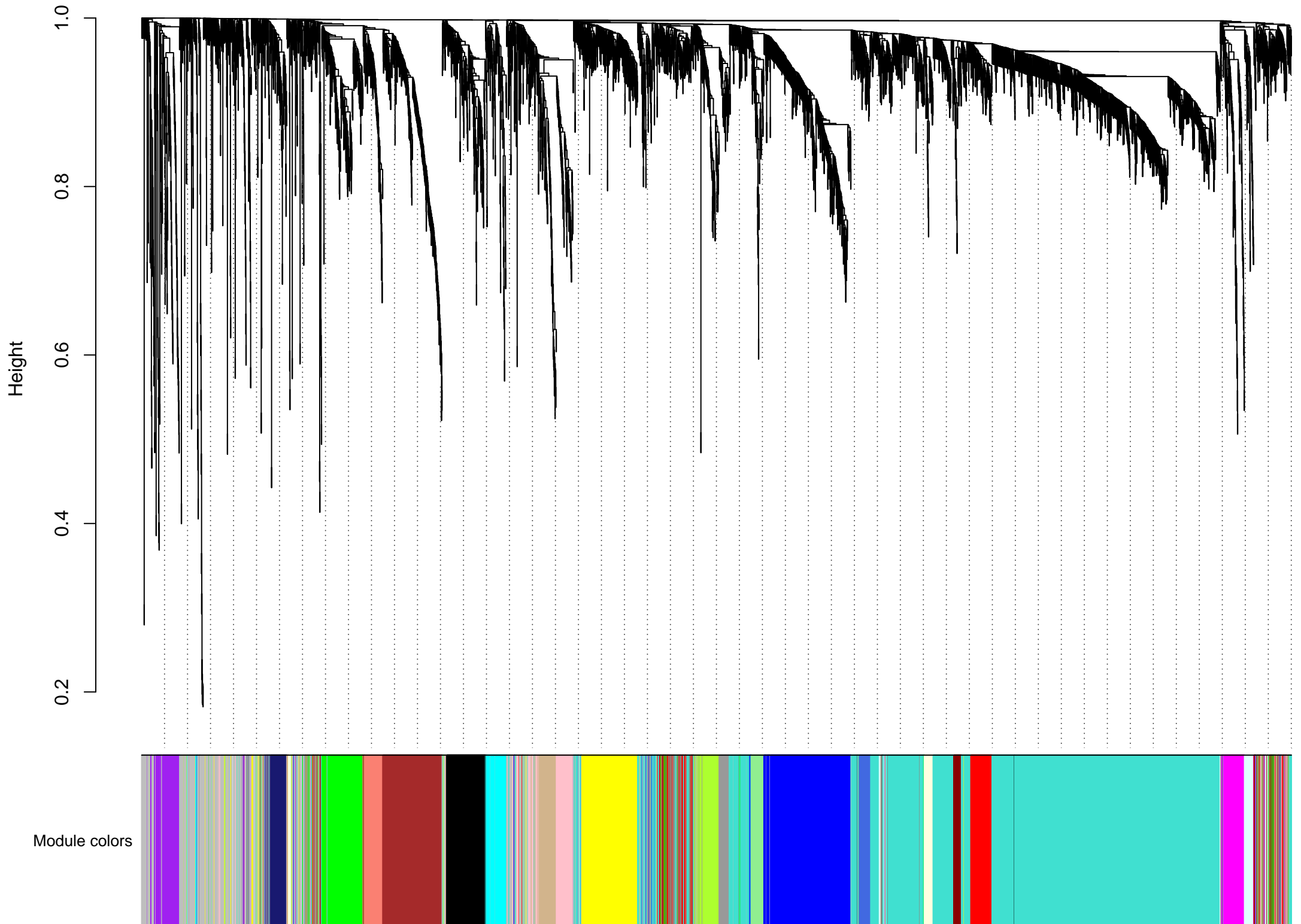

Supplement: Supplementary file 6 [file DataSheet_6.zip › Step6/wgcna/Step02-moduleCluster.pdf]

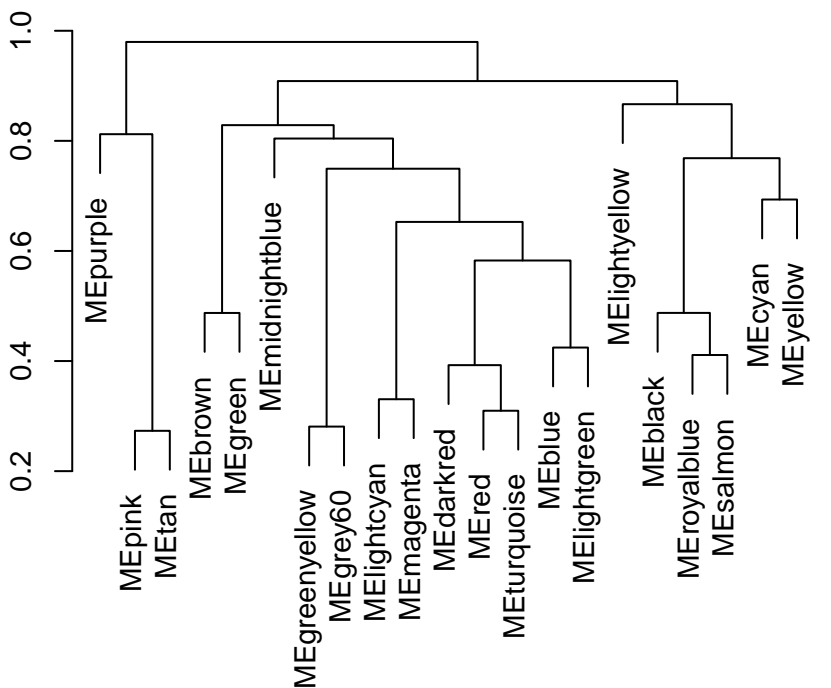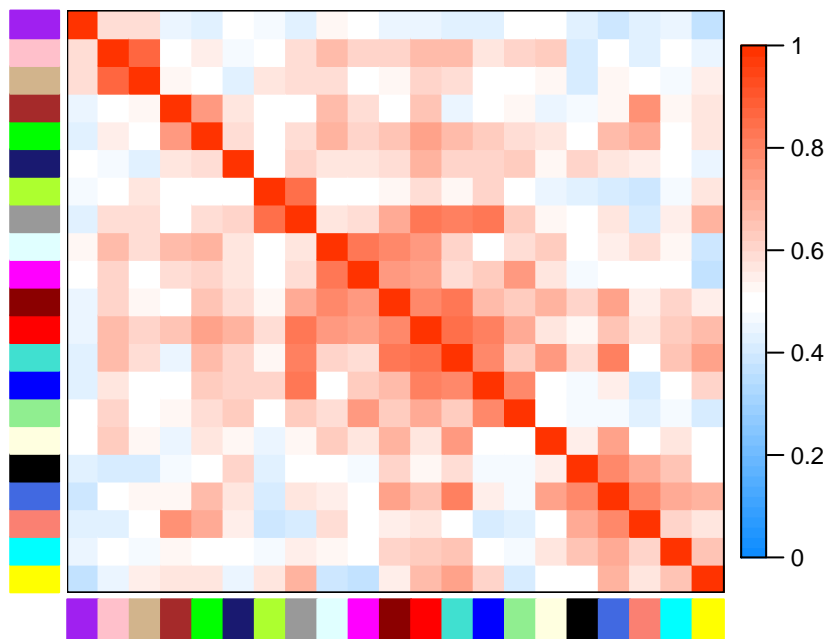

Supplement: Supplementary file 6 [file DataSheet_6.zip › Step6/wgcna/Step02-moduleCor.pdf]

Histogram of k

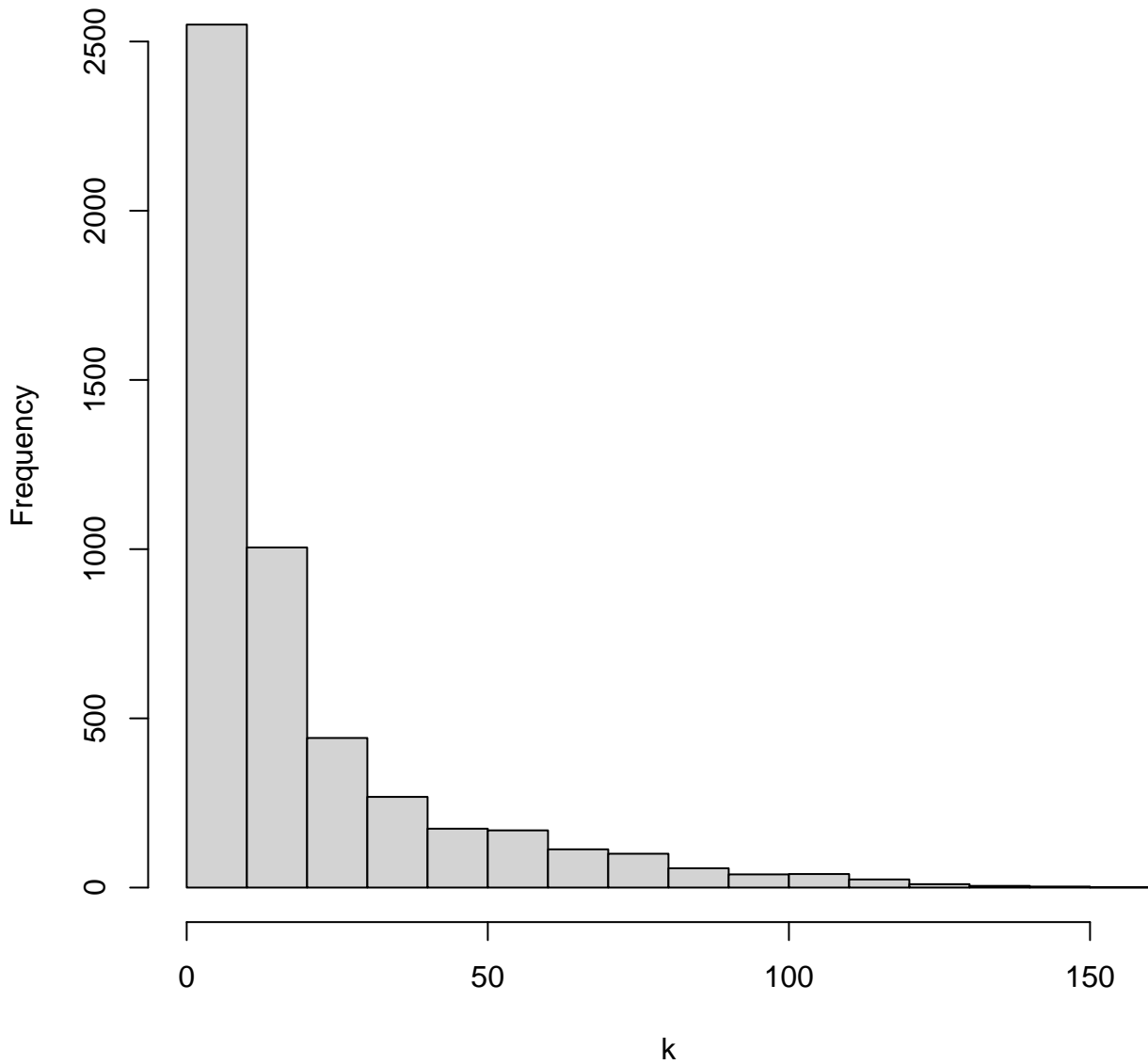

Check scale free topology scale  $R^2= 0.85$  , slope=  $-1.81$

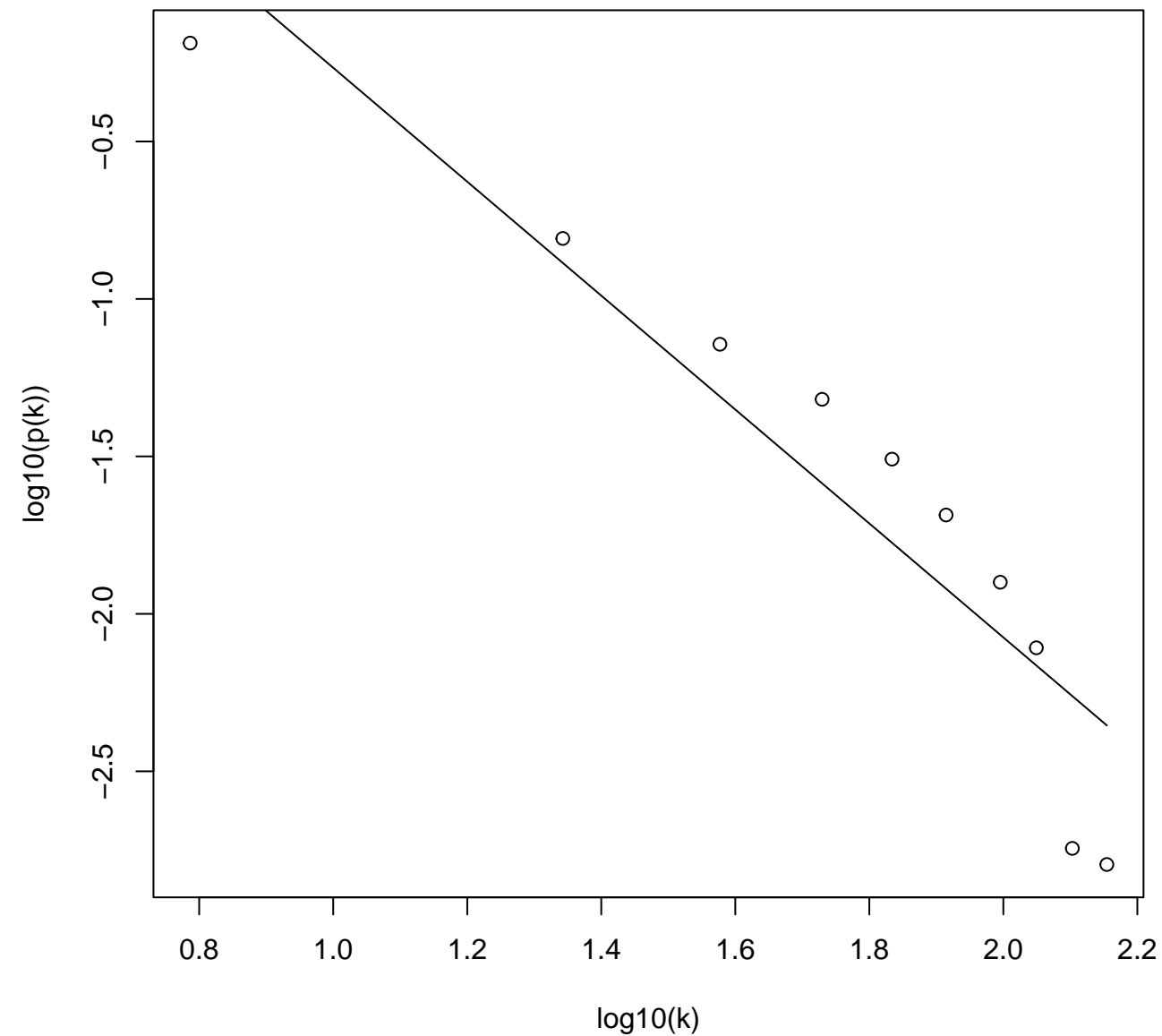

Supplement: Supplementary file 6 [file DataSheet_6.zip › Step6/wgcna/Step02-scaleFree.pdf]

### Scale independence

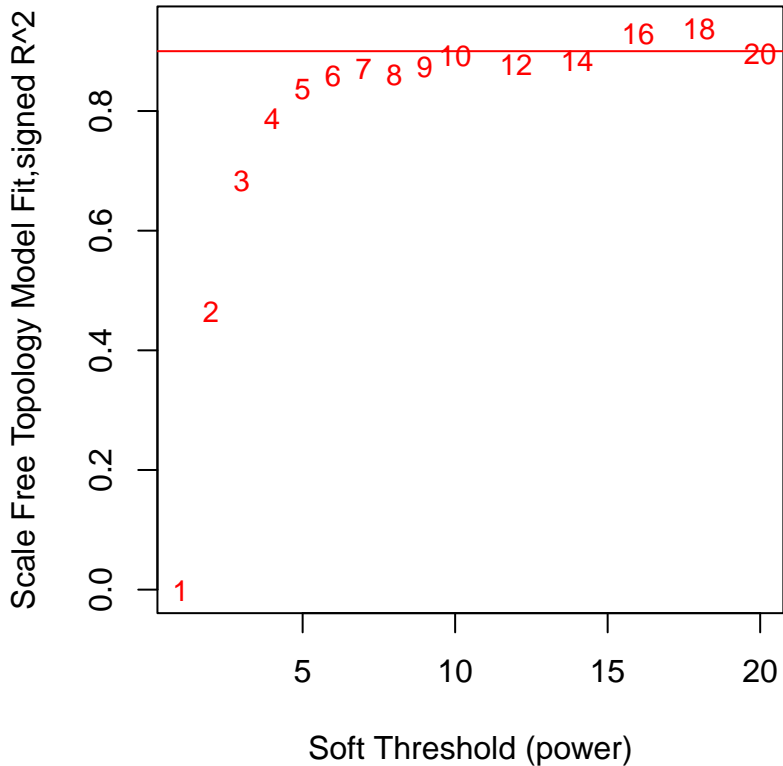

### Mean connectivity

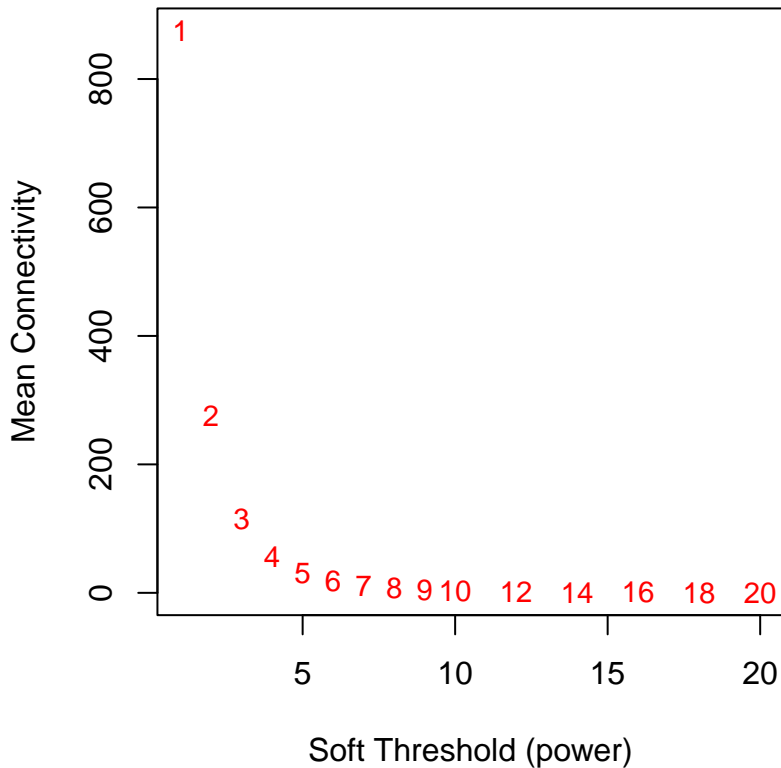

Supplement: Supplementary file 6 [file DataSheet_6.zip › Step6/wgcna/Step02-SoftThreshold.pdf]

Network heatmap plot, selected genes

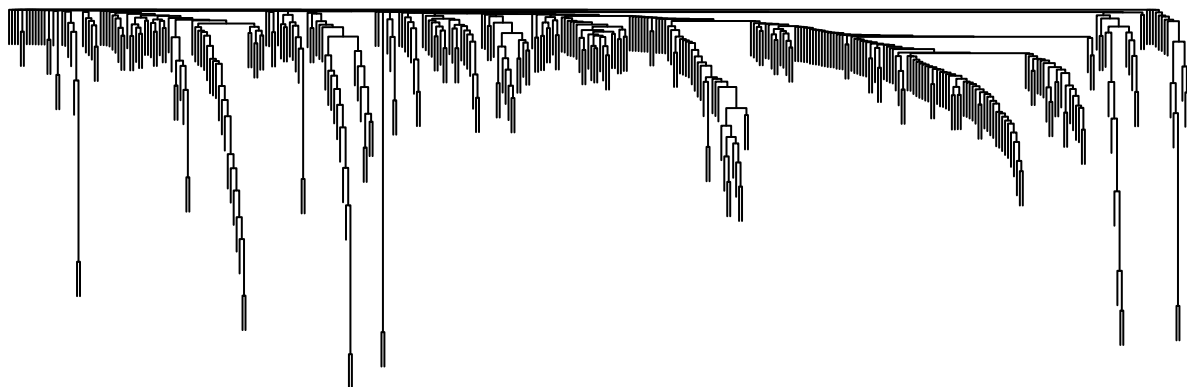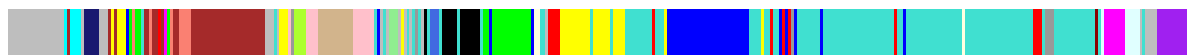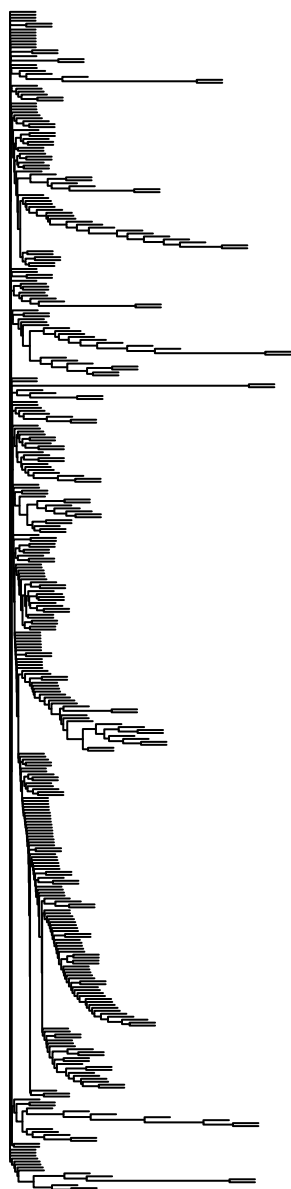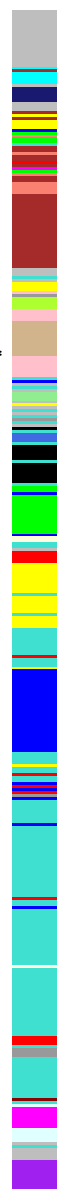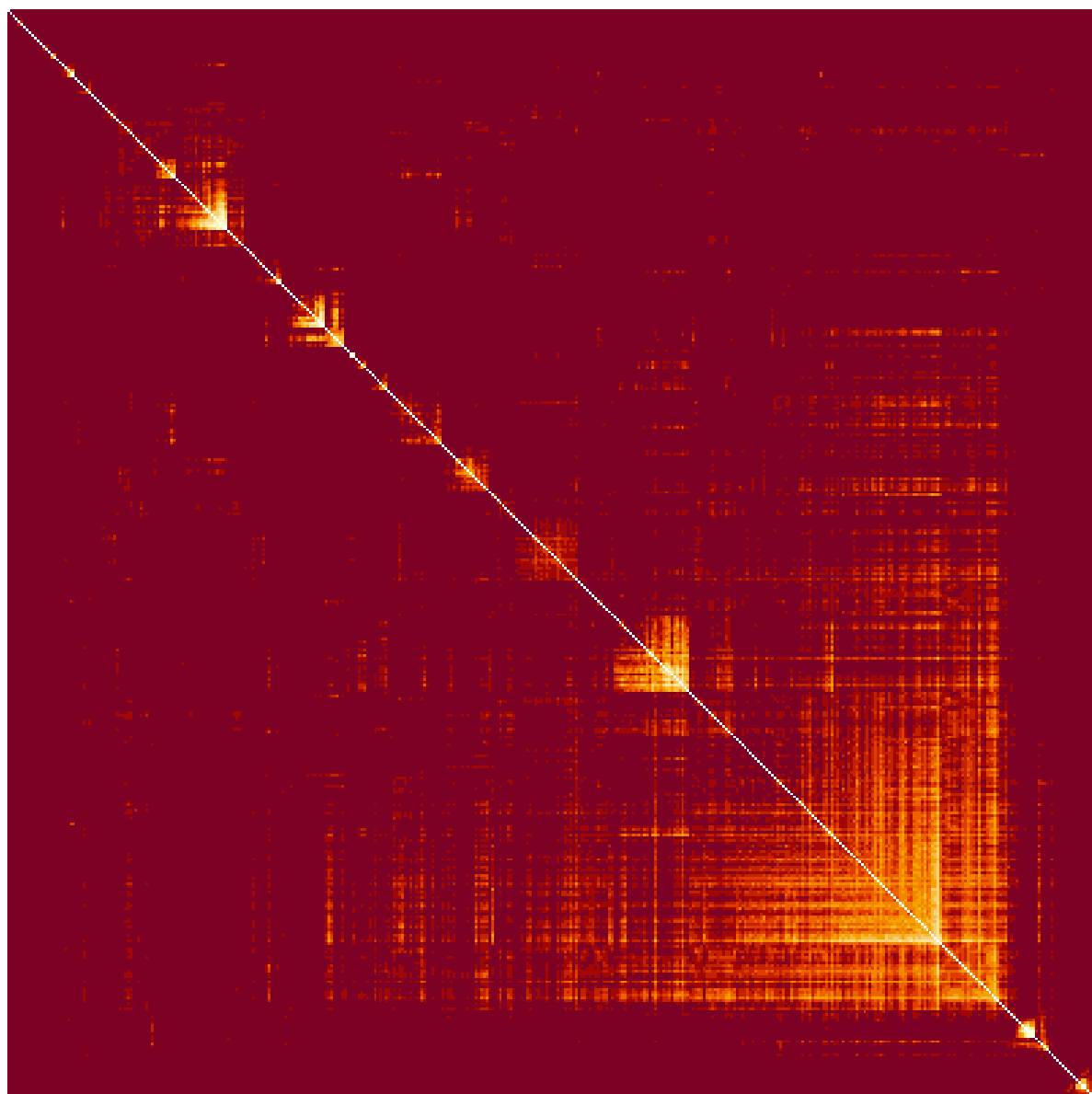

Supplement: Supplementary file 6 [file DataSheet_6.zip › Step6/wgcna/Step02-TOMplot.pdf]

**Module membership vs. gene significance**  
**cor=0.88,  $p < 1e-200$**

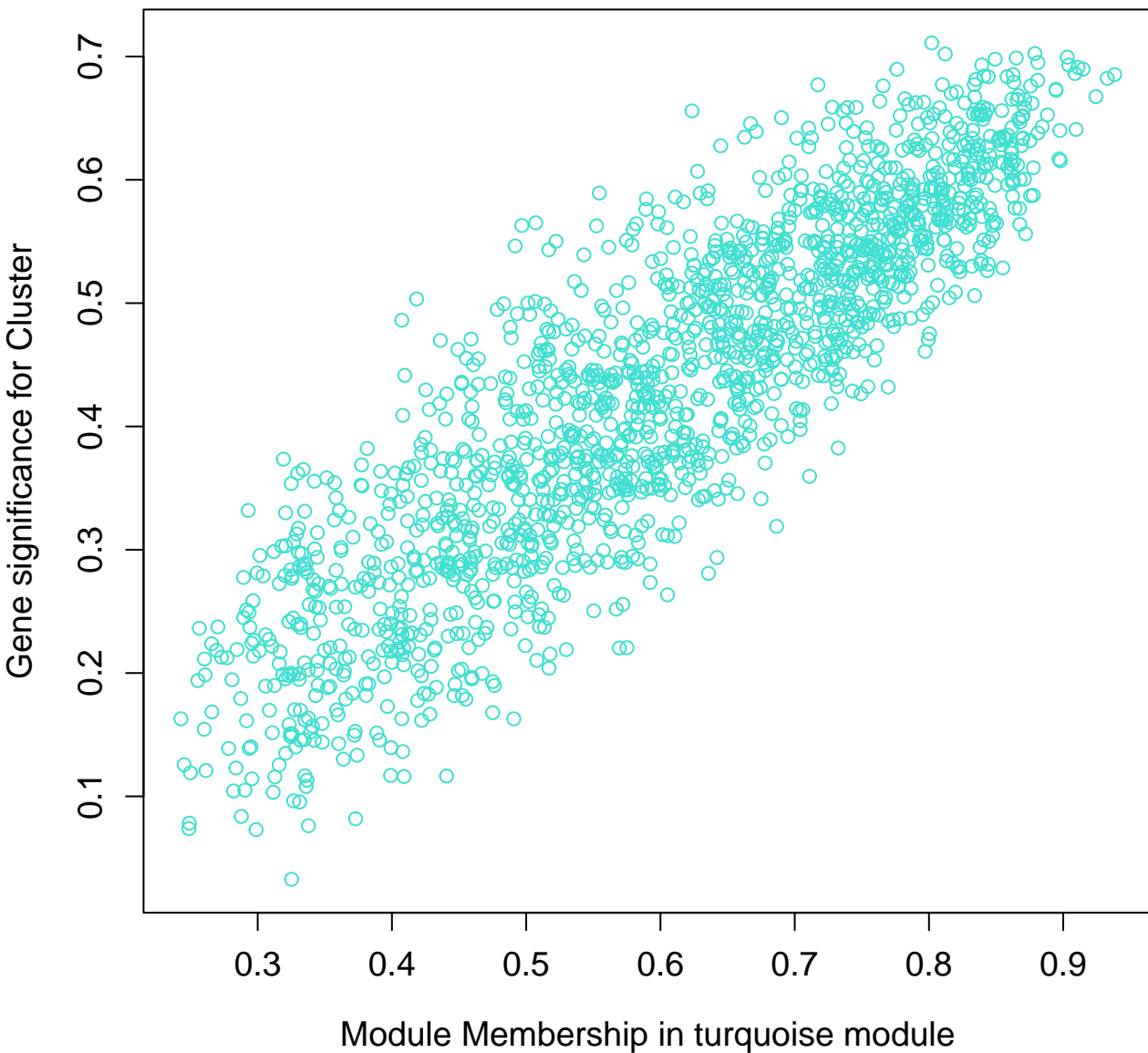

Supplement: Supplementary file 6 [file DataSheet_6.zip › Step6/wgcna/Step04-Module_membership_vs_gene_significance.pdf]

# Module-trait relationships

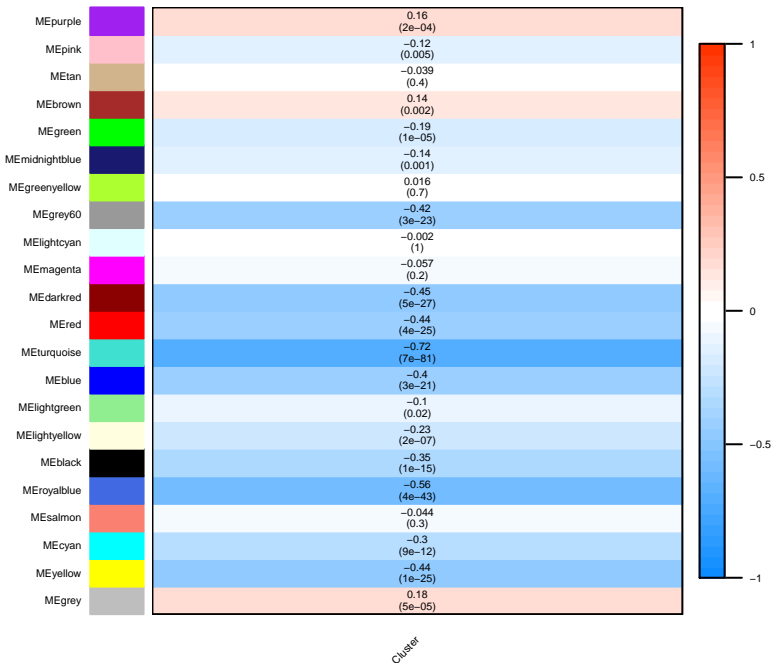

Supplement: Supplementary file 6 [file DataSheet_6.zip › Step6/wgcna/Step04-Module_trait_relationships(1).pdf]

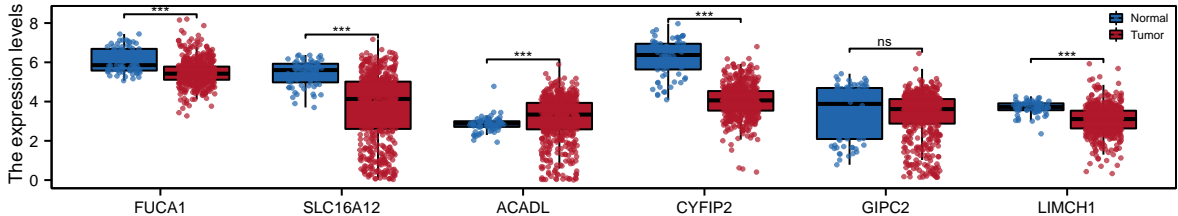

Supplement: Supplementary file 6 [file DataSheet_6.zip › Step6/▒φ┤∩▓ε╥∞.pdf]

riskScore

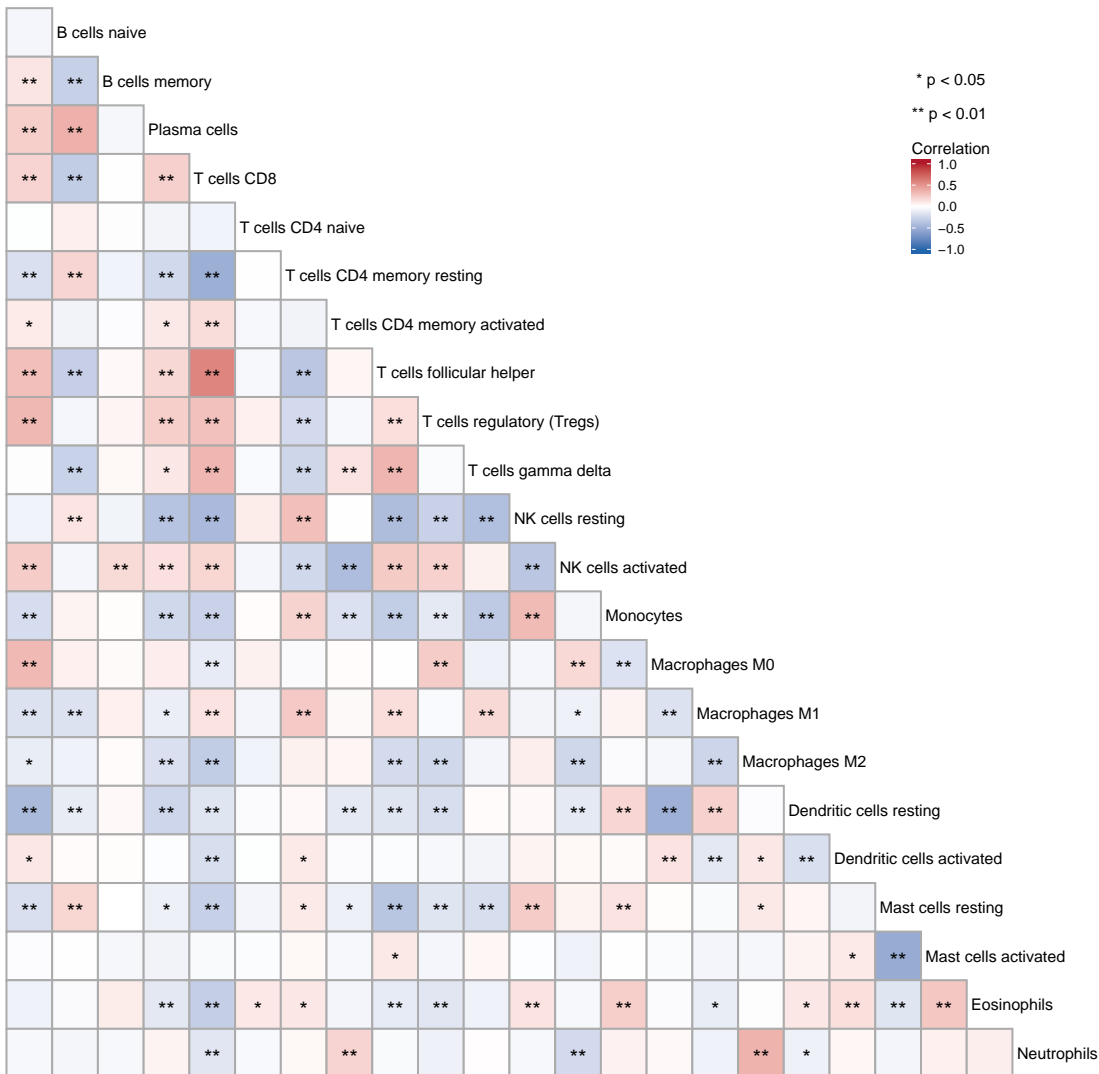

Supplement: Supplementary file 7 [file DataSheet_7.zip › Step7/immu/╧α╣╪╨╘╚╚═╝_immucells.pdf]

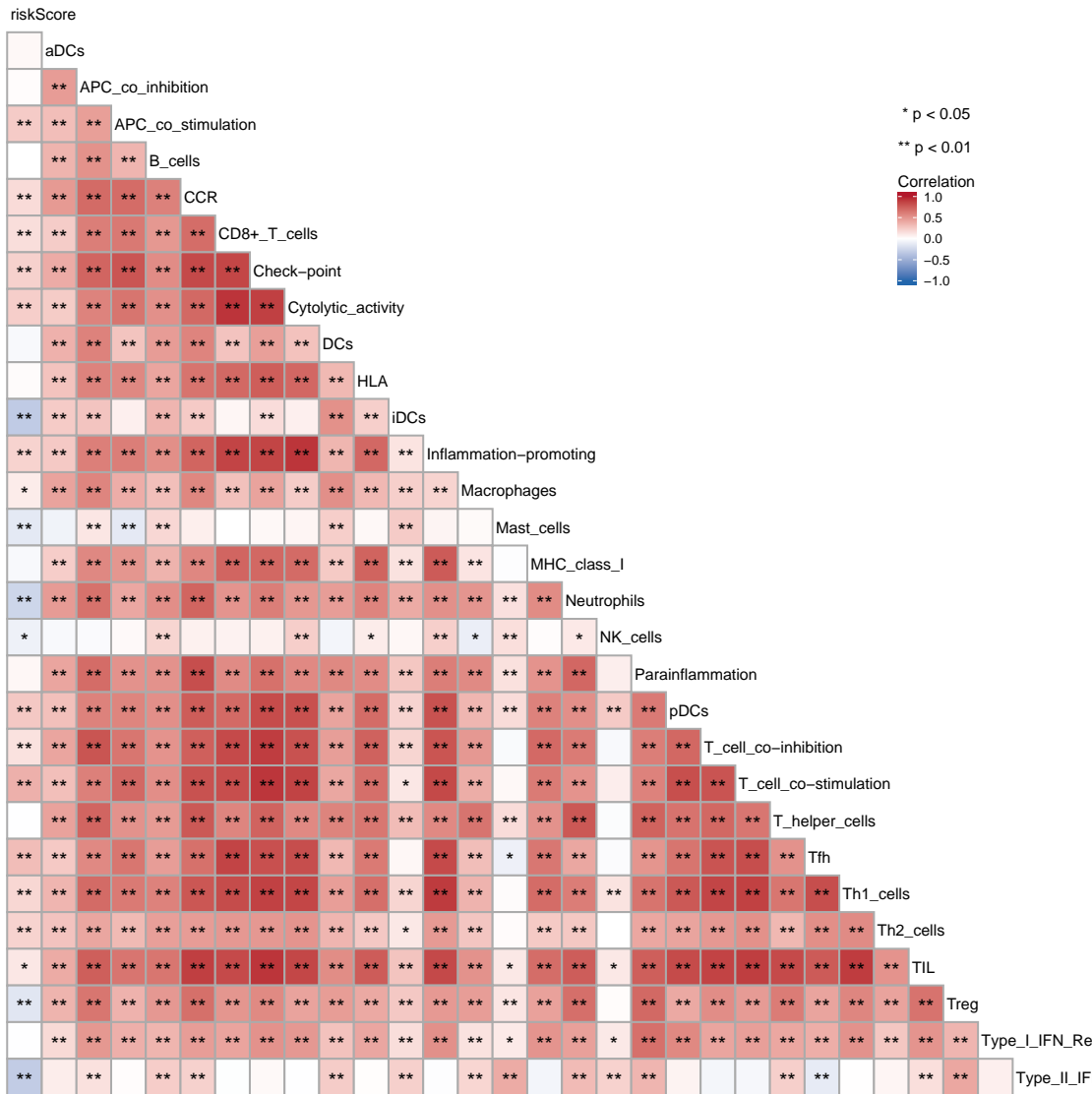

Supplement: Supplementary file 7 [file DataSheet_7.zip › Step7/immu/╧α╣╪╨╘╚╚═╝_immufun.pdf]

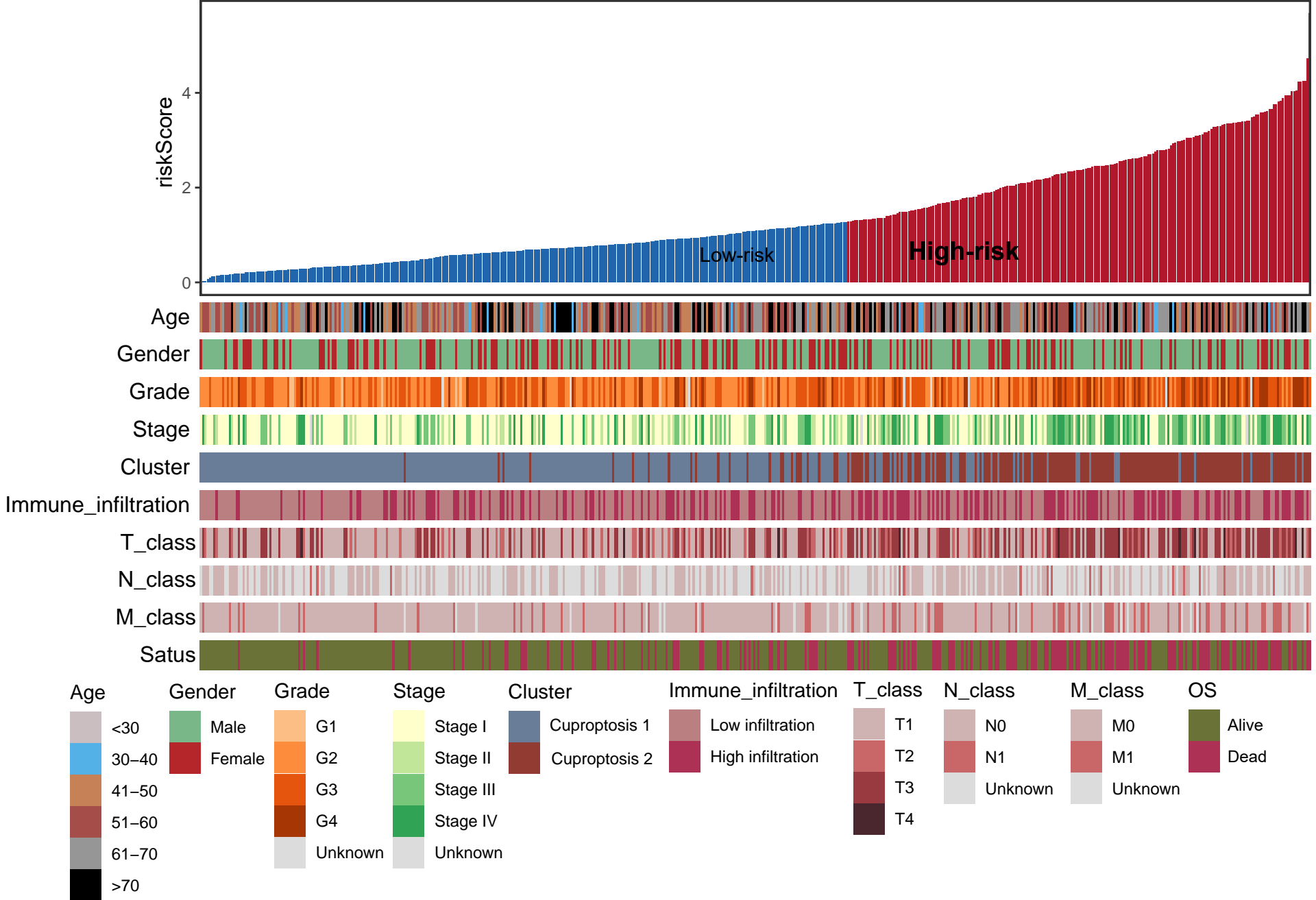

Supplement: Supplementary file 7 [file DataSheet_7.zip › Step7/risk/1.pdf]

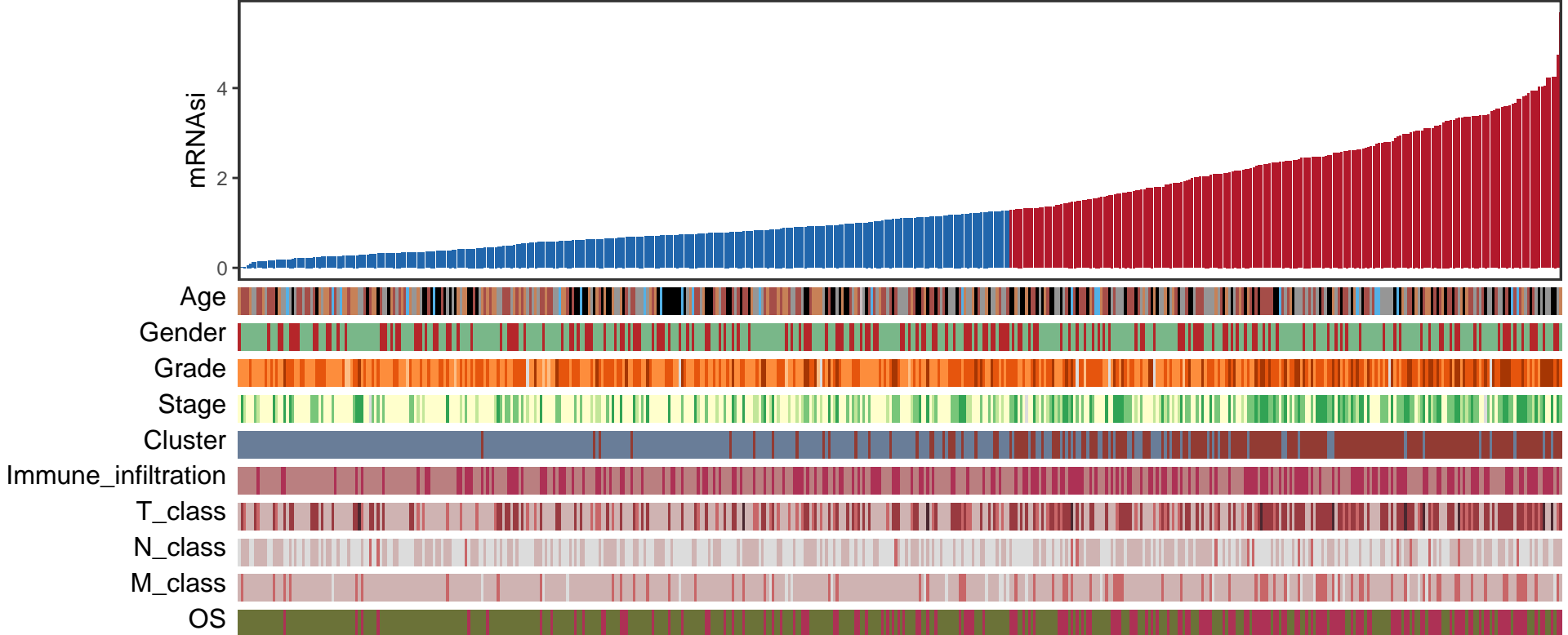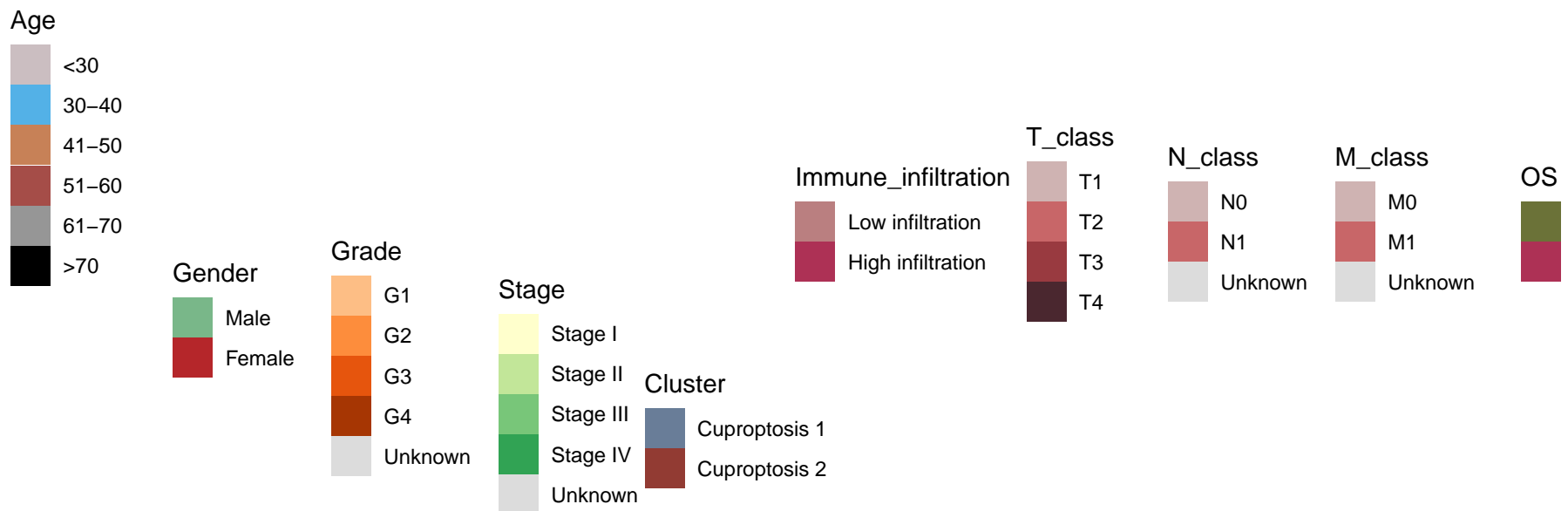

Supplement: Supplementary file 7 [file DataSheet_7.zip › Step7/risk/mRNAsi.pdf]

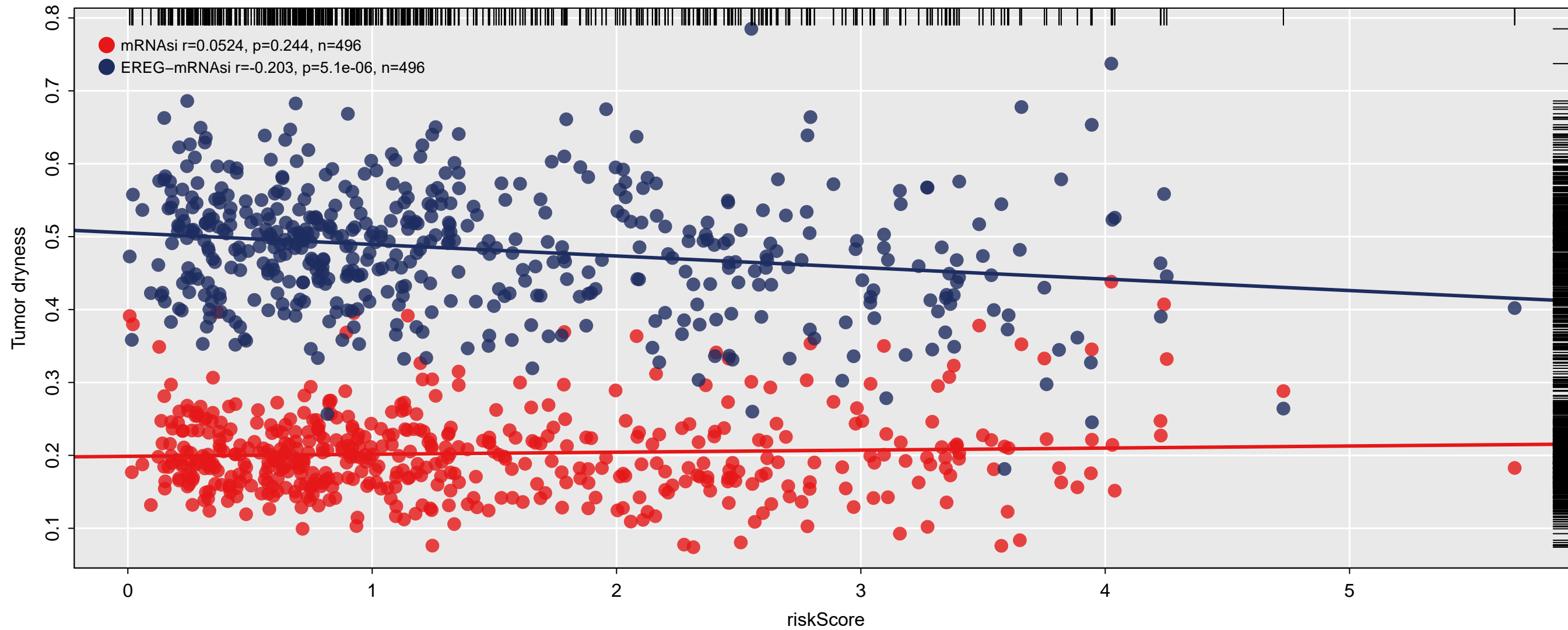

Supplement: Supplementary file 7 [file DataSheet_7.zip › Step7/STEM/Rplot02.pdf]

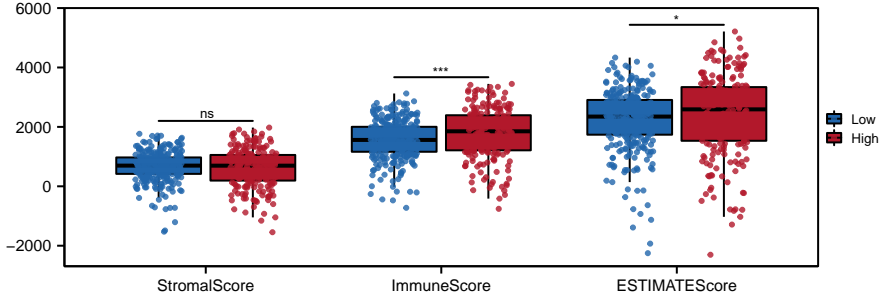

Supplement: Supplementary file 7 [file DataSheet_7.zip › Step7/╖╓╫Θ▒╚╜╧═╝_2022-04-06_13_14_13.pdf]

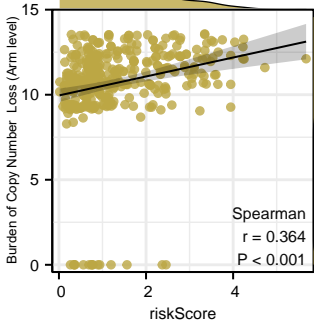

Supplement: Supplementary file 7 [file DataSheet_7.zip › Step7/╔ó╡π═╝_2022-04-06_21_04_06.pdf]

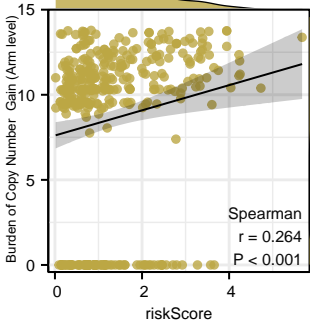

Supplement: Supplementary file 7 [file DataSheet_7.zip › Step7/╔ó╡π═╝_2022-04-06_21_04_42.pdf]

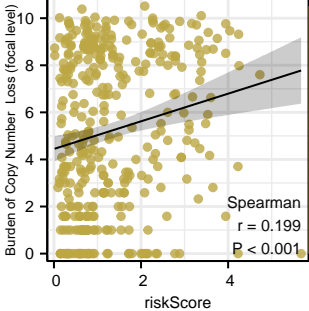

Supplement: Supplementary file 7 [file DataSheet_7.zip › Step7/╔ó╡π═╝_2022-04-06_21_08_43.pdf]

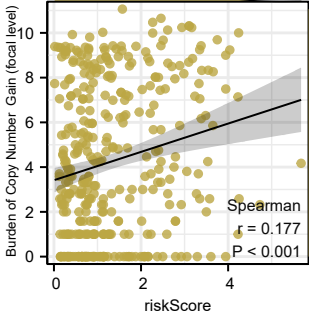

Supplement: Supplementary file 7 [file DataSheet_7.zip › Step7/╔ó╡π═╝_2022-04-06_21_09_10.pdf]
