# Supplementary material for: Cuproptosis in ccRCC: key player in therapeutic and prognostic targets
Source: Front Oncol. 2023 Oct 27;13:1271864. doi: 10.3389/fonc.2023.1271864 (PMC10642186; doi:10.3389/fonc.2023.1271864)
Supplement: Supplementary file 3 [file DataSheet_3.zip › Step3/mut/forestplot.pdf]

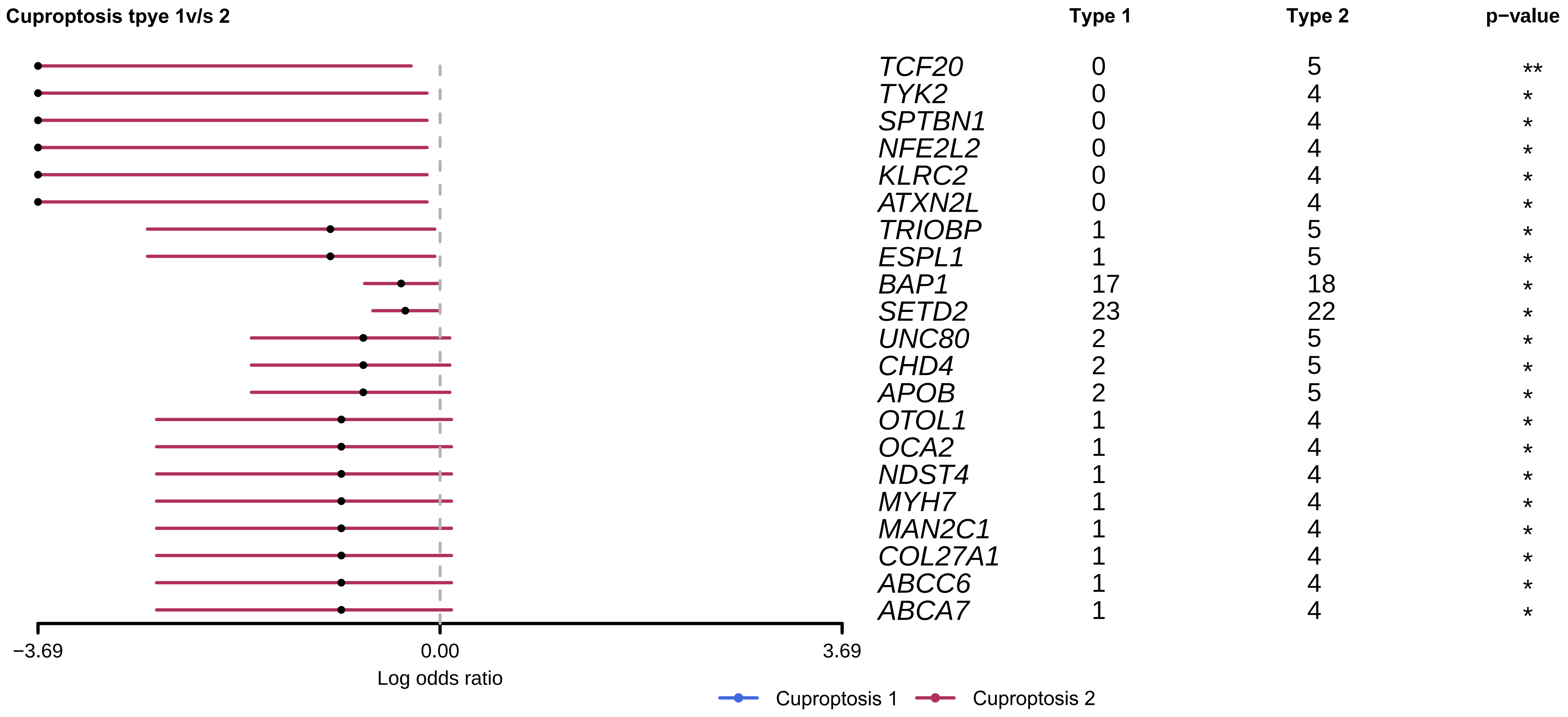
Cuproptosis tpye 1v/s 2
Type 1
Type 2
p−value
TCF20 0 5 **
TYK2 0 4 *
SPTBN1 0 4 *
NFE2L2 0 4 *
KLRC2 0 4 *
ATXN2L 0 4 *
TRIOBP 1 5 *
ESPL1 1 5 *
BAP1 17 18 *
SETD2 23 22 *
UNC80 2 5 *
CHD4 2 5 *
APOB 2 5 *
OTOL1 1 4 *
OCA2 1 4 *
NDST4 1 4 *
MYH7 1 4 *
MAN2C1 1 4 *
COL27A1 1 4 *
ABCC6 1 4 *
ABCA7 1 4 *
−3.69
0.00
3.69
Log odds ratio
Cuproptosis 1
Cuproptosis 2
