# Supplementary material for: Cuproptosis in ccRCC: key player in therapeutic and prognostic targets
Source: Front Oncol. 2023 Oct 27;13:1271864. doi: 10.3389/fonc.2023.1271864 (PMC10642186; doi:10.3389/fonc.2023.1271864)
Supplement: Supplementary file 4 [file DataSheet_4.zip › Step4/immu/immFunction.pdf]

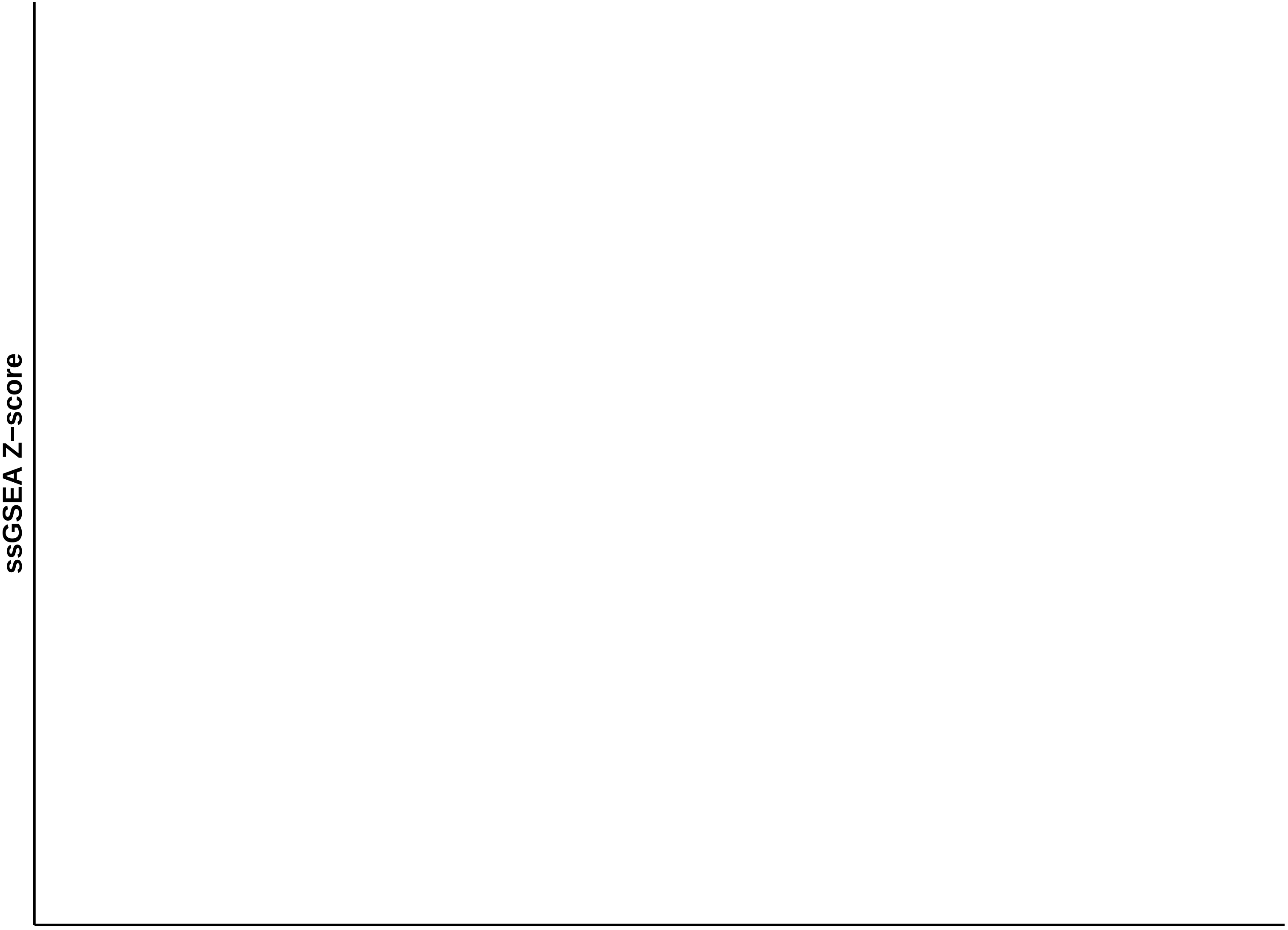
ssGSEA Z−score
