# Supplementary material for: Cuproptosis in ccRCC: key player in therapeutic and prognostic targets
Source: Front Oncol. 2023 Oct 27;13:1271864. doi: 10.3389/fonc.2023.1271864 (PMC10642186; doi:10.3389/fonc.2023.1271864)
Supplement: Supplementary file 5 [file DataSheet_5.zip › Step5/vol/FigureYa59volcanoV2/FigureYa59volcanoV2.html]

FigureYa59volcano V2


# FigureYa59volcano V2

#### *小丫画图出品*

#### *2018-12-9*

“小丫画图”公众号: FigureYa，小丫微信：epigenomics，E-mail: figureya@126.com

作者：Haitao Wang, 小丫, Jianming Zeng and Dapeng Hao

Dr. Haitao Wang: JNU,Guagnzhou => GIBH-CAS,Guagnzhou => BNU,Beijing => IMCB,Singapore => UMAC,Macao => NCCS,Singapore

Email: ht.wang@yahoo.com; wang.haitao@nccs.com.sg

小丫编辑校验

## 需求描述

画出像paper里这样美的火山图。

关注“小丫画图”公众号（微信ID：FigureYa），回复“火山”，查看火山图在paper里的高级用法。

出自https://www.nature.com/articles/s41467-018-06944-1

## 应用场景

展示两组之间总体的基因表达变化趋势。

## 环境设置

```
#使用国内镜像安装包
#options("repos"= c(CRAN="https://mirrors.tuna.tsinghua.edu.cn/CRAN/"))
#options(BioC_mirror="http://mirrors.ustc.edu.cn/bioc/")

library(ggplot2)
library(ggrepel)
library(ggthemes)

Sys.setenv(LANGUAGE = "en") #显示英文报错信息
options(stringsAsFactors = FALSE) #禁止chr转成factor
```

## 输入文件

- easy\_input\_limma.csv，此处用limma结果，还可以用EdgeR、DESeq2等工具获得的差异分析结果作为输入。

至少包含三列：基因名、变化倍数（logFC）、P value(P.Value)或adjust Pvalue(adj.P.Val) 最后一列pathway为可选项，是每个基因所在的pathway，不同pathway的基因名将用不同颜色的字突出显示出来。

- easy\_input\_selected.csv，有时希望突出显示几个基因，例如后文进行功能验证的基因。会在突出显示的基因外面画黑色圆圈，并标出基因名。

至少包含第一列：基因名。第二列是该基因所在的pathway，为可选项。

```
# 全部基因差异表达分析结果
x <- read.csv("easy_input_limma.csv", row.names = 1)
x$label<- rownames(x)
head(x)
```

```
##           logFC   AveExpr         t      P.Value    adj.P.Val        B
## KLK10  8.778049 10.506750 111.57547 3.802229e-11 1.760315e-07 15.41764
## FXYD3  7.748971 10.459578 107.27217 4.809044e-11 1.760315e-07 15.29791
## KLK7   9.138924 10.596380 102.65832 6.252983e-11 1.760315e-07 15.15779
## SPRR1B 9.646357 10.570985 101.27838 6.779348e-11 1.760315e-07 15.11331
## KLK8   7.419248  8.592425 100.42890 7.129085e-11 1.760315e-07 15.08531
## SPRR1A 9.001559 10.692375  98.45984 8.023989e-11 1.760315e-07 15.01850
##         label
## KLK10   KLK10
## FXYD3   FXYD3
## KLK7     KLK7
## SPRR1B SPRR1B
## KLK8     KLK8
## SPRR1A SPRR1A
```

```
# 突出展示感兴趣的基因
selectedGeneID <- read.csv("easy_input_selected.csv")
head(selectedGeneID)
```

```
##    gsym  pathway
## 1 KLK10 pathway1
## 2  KLK7 pathway1
## 3  KLK8 pathway1
## 4 KRT16 pathway2
## 5  KLK5 pathway2
## 6 KRT6A pathway2
```

```
# 提取感兴趣的基因的差异分析结果
x$gsym <- row.names(x)
selectgenes <- merge(selectedGeneID, x, by = "gsym")
head(selectgenes)
```

```
##     gsym  pathway     logFC   AveExpr         t      P.Value    adj.P.Val
## 1  KCNK1 pathway3  4.348004  9.275305  48.07464 5.787268e-09 1.229221e-06
## 2 KCTD12 pathway3 -3.597860  9.976829 -49.23835 5.018151e-09 1.188589e-06
## 3  KLK10 pathway1  8.778049 10.506750 111.57547 3.802229e-11 1.760315e-07
## 4   KLK5 pathway2  7.567174 10.651720  67.88031 7.390827e-10 4.483544e-07
## 5   KLK7 pathway1  9.138924 10.596380 102.65832 6.252983e-11 1.760315e-07
## 6   KLK8 pathway1  7.419248  8.592425 100.42890 7.129085e-11 1.760315e-07
##          B  label
## 1 11.74301  KCNK1
## 2 11.87553 KCTD12
## 3 15.41764  KLK10
## 4 13.51478   KLK5
## 5 15.15779   KLK7
## 6 15.08531   KLK8
```

## 参数设置

点的颜色和虚线的位置都由下面的阈值决定，根据具体需求调整。

```
#plot_mode <- "classic" #经典版
plot_mode <- "advanced" #酷炫版

logFCcut <- 1.5 #log2-foldchange
pvalCut <- 0.05 #P.value
adjPcut <- 0.05 #adj.P.value

#for advanced mode
logFCcut2 <- 2.5
logFCcut3 <- 5
pvalCut2 <- 0.0001
pvalCut3 <- 0.00001

#置x，y軸的最大最小位置
xmin <- (range(x$logFC)[1]- (range(x$logFC)[1]+ 10))
xmax <- (range(x$logFC)[1]+ (10-range(x$logFC)[1]))
ymin <- 0
ymax <- -log10(x$P.Value)[3] * 1.1

# 基因名的颜色，需大于等于pathway的数量，这里自定义了足够多的颜色
mycol <- c("darkgreen","chocolate4","blueviolet","#223D6C","#D20A13","#088247","#58CDD9","#7A142C","#5D90BA","#431A3D","#91612D","#6E568C","#E0367A","#D8D155","#64495D","#7CC767")
```

## 开始画图

```
if (plot_mode == "classic"){
  # 簡單的setting for color
  x$color_transparent <- ifelse((x$P.Value < pvalCut & x$logFC > logFCcut), "red", ifelse((x$P.Value < pvalCut & x$logFC < -logFCcut), "blue","grey30"))
  # 簡單的setting for size
  size <- ifelse((x$P.Value < pvalCut & abs(x$logFC) > logFCcut), 4, 2)
  
} else if (plot_mode == "advanced") {
  # 複雜的的setting for color
  n1 <- length(x[, 1])
  cols <- rep("grey30", n1)
  names(cols)<- rownames(x)
  
  #不同阈值的点的颜色
  cols[x$P.Value < pvalCut & x$logFC >logFCcut]<- "#FB9A99"
  cols[x$P.Value < pvalCut2 & x$logFC > logFCcut2]<- "#ED4F4F"
  cols[x$P.Value < pvalCut & x$logFC < -logFCcut]<- "#B2DF8A"
  cols[x$P.Value < pvalCut2 & x$logFC < -logFCcut2]<- "#329E3F"
  color_transparent <- adjustcolor(cols, alpha.f = 0.5)
  x$color_transparent <- color_transparent
  
  # 複雜的的setting for size
  n1 <- length(x[, 1])
  size <- rep(1, n1)
  
  #不同阈值的点的大小
  size[x$P.Value < pvalCut & x$logFC > logFCcut]<- 2
  size[x$P.Value < pvalCut2 & x$logFC > logFCcut2]<- 4
  size[x$P.Value < pvalCut3 & x$logFC > logFCcut3]<- 6
  size[x$P.Value < pvalCut & x$logFC < -logFCcut]<- 2
  size[x$P.Value < pvalCut2 & x$logFC < -logFCcut2]<- 4
  size[x$P.Value < pvalCut3 & x$logFC < -logFCcut3]<- 6
  
} else {
  stop("Unsupport mode")
}

# Construct the plot object
p1 <- ggplot(data=x, aes(logFC, -log10(P.Value), label = label, color = pathway)) +
  geom_point(alpha = 0.6, size = size, colour = x$color_transparent) +

  labs(x=bquote(~Log[2]~"(fold change)"), y=bquote(~-Log[10]~italic("P-value")), title="") + 
  ylim(c(ymin,ymax)) + 
  scale_x_continuous(
    breaks = c(-10, -5, -logFCcut, 0, logFCcut, 5, 10), #刻度线的位置
    labels = c(-10, -5, -logFCcut, 0, logFCcut, 5, 10),
    limits = c(-11, 11) #x轴范围，两侧对称才好看
  ) +
  #或用下面这行：
  #xlim(c(xmin, xmax)) + 

  #画阈值分界线
  geom_vline(xintercept = c(-logFCcut, logFCcut), color="grey40", 
             linetype="longdash", lwd = 0.5) + #虚线的形状和粗细
  geom_hline(yintercept = -log10(pvalCut), color="grey40", 
             linetype="longdash", lwd = 0.5) +
  
  theme_bw(base_size = 12#, base_family = "Times" #修改字体
           ) +
  theme(panel.grid=element_blank())

if (plot_mode == "advanced") {
  p1 <- p1 + 
  geom_vline(xintercept = c(-logFCcut2, logFCcut2), color="grey40", 
             linetype="longdash", lwd = 0.5) +
  geom_hline(yintercept = -log10(pvalCut2), color="grey40", 
             linetype="longdash", lwd = 0.5)
}
p1
```

```
# 显示 logFC > n 的基因的基因名
n = 9
p1 + geom_text_repel(aes(x = logFC, y = -log10(P.Value), 
                               label = ifelse(logFC > n, rownames(x),"")),
        colour="darkred", size = 5, box.padding = unit(0.35, "lines"), 
        point.padding = unit(0.3, "lines"))
```

```
# 突出显示候选基因
p2 <- p1 + 
  # 在感兴趣的基因外面画个黑色圈
  geom_point(data = selectgenes, alpha = 1, size = 4.6, shape = 1, 
             stroke = 1, #圈粗细
             color = "black") +
  
  # 显示感兴趣的基因的基因名
  scale_color_manual(values = mycol) + 
  geom_text_repel(data = selectgenes, 
                  show.legend = FALSE, #不显示图例
                  size = 5, box.padding = unit(0.35, "lines"), 
        point.padding = unit(0.3, "lines")) +
  guides(color=guide_legend(title = NULL)) 

p2
```

```
# 保存到PDF文件
if (plot_mode == "classic"){ggsave("volcano_classic.pdf", width=6,height=5)} else if (plot_mode == "advanced") {ggsave("Volcano_advanced.pdf",width=6,height=5)} else {stop("Unsupport mode")}
```

```
sessionInfo()
```

```
## R version 3.5.1 (2018-07-02)
## Platform: x86_64-apple-darwin15.6.0 (64-bit)
## Running under: macOS  10.14.1
## 
## Matrix products: default
## BLAS: /Library/Frameworks/R.framework/Versions/3.5/Resources/lib/libRblas.0.dylib
## LAPACK: /Library/Frameworks/R.framework/Versions/3.5/Resources/lib/libRlapack.dylib
## 
## locale:
## [1] en_US.UTF-8/en_US.UTF-8/en_US.UTF-8/C/en_US.UTF-8/en_US.UTF-8
## 
## attached base packages:
## [1] stats     graphics  grDevices utils     datasets  methods   base     
## 
## other attached packages:
## [1] ggthemes_4.0.1 ggrepel_0.8.0  ggplot2_3.1.0 
## 
## loaded via a namespace (and not attached):
##  [1] Rcpp_1.0.0       rstudioapi_0.8   bindr_0.1.1      knitr_1.20      
##  [5] magrittr_1.5     tidyselect_0.2.5 munsell_0.5.0    colorspace_1.3-2
##  [9] R6_2.3.0         rlang_0.3.0.1    stringr_1.3.1    plyr_1.8.4      
## [13] dplyr_0.7.8      tools_3.5.1      grid_3.5.1       gtable_0.2.0    
## [17] withr_2.1.2      htmltools_0.3.6  assertthat_0.2.0 yaml_2.2.0      
## [21] lazyeval_0.2.1   rprojroot_1.3-2  digest_0.6.18    tibble_1.4.2    
## [25] crayon_1.3.4     bindrcpp_0.2.2   purrr_0.2.5      glue_1.3.0      
## [29] evaluate_0.12    rmarkdown_1.10   labeling_0.3     stringi_1.2.4   
## [33] compiler_3.5.1   pillar_1.3.0     scales_1.0.0     backports_1.1.2 
## [37] pkgconfig_2.0.2
```
