# Supplementary figures and images for: Cuproptosis in ccRCC: key player in therapeutic and prognostic targets (part 2 of 2)
Source: Front Oncol. 2023 Oct 27;13:1271864. doi: 10.3389/fonc.2023.1271864 (PMC10642186; doi:10.3389/fonc.2023.1271864)

sign

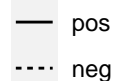

pSeg

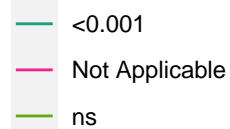

rSeg

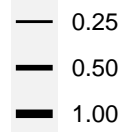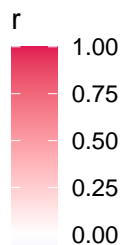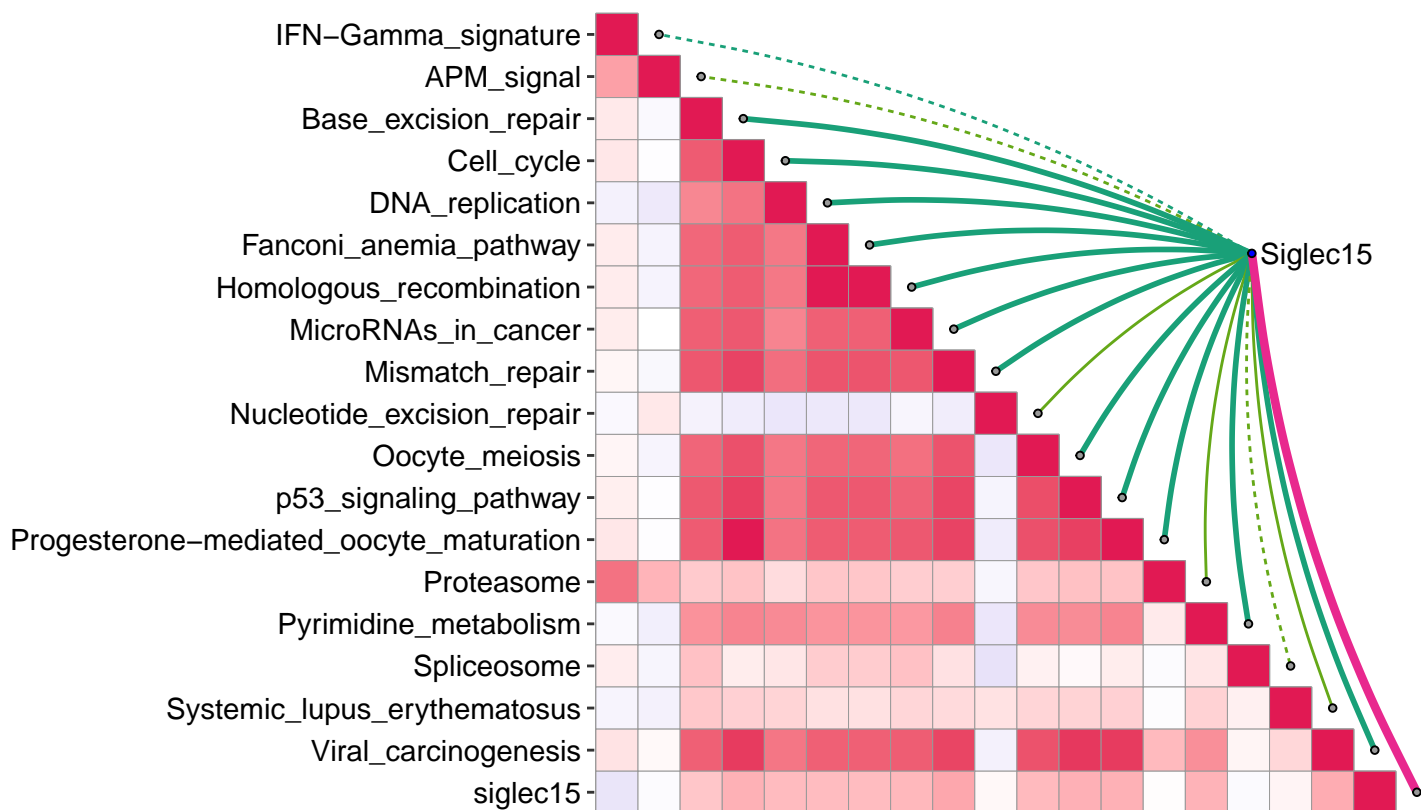

Supplement: Supplementary file 7 [file DataSheet_7.zip › Step7/╨┬╜¿╬─╝■╝╨/ggcor plot in bottom left.pdf]

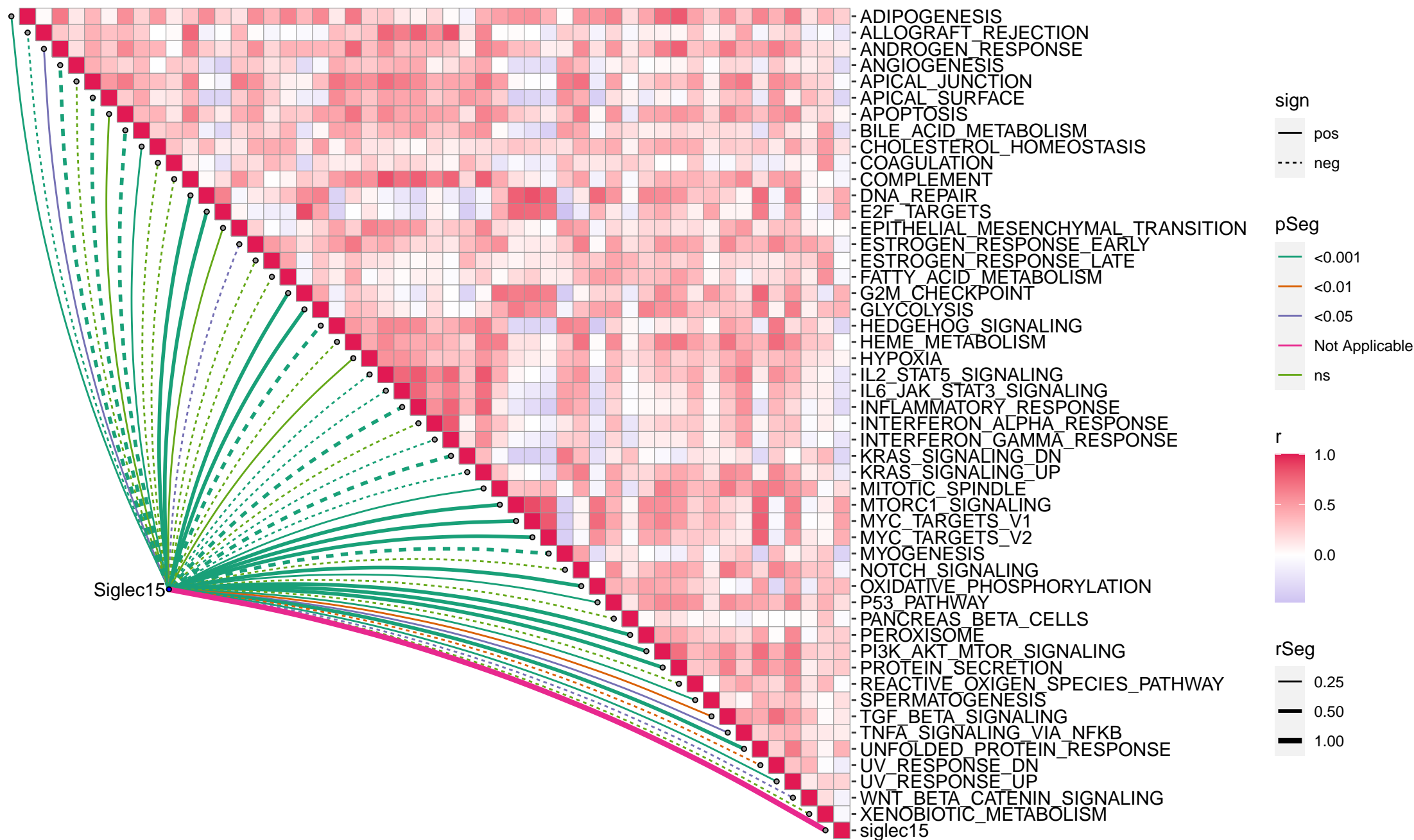

Supplement: Supplementary file 7 [file DataSheet_7.zip › Step7/╨┬╜¿╬─╝■╝╨/ggcor plot in top right.pdf]

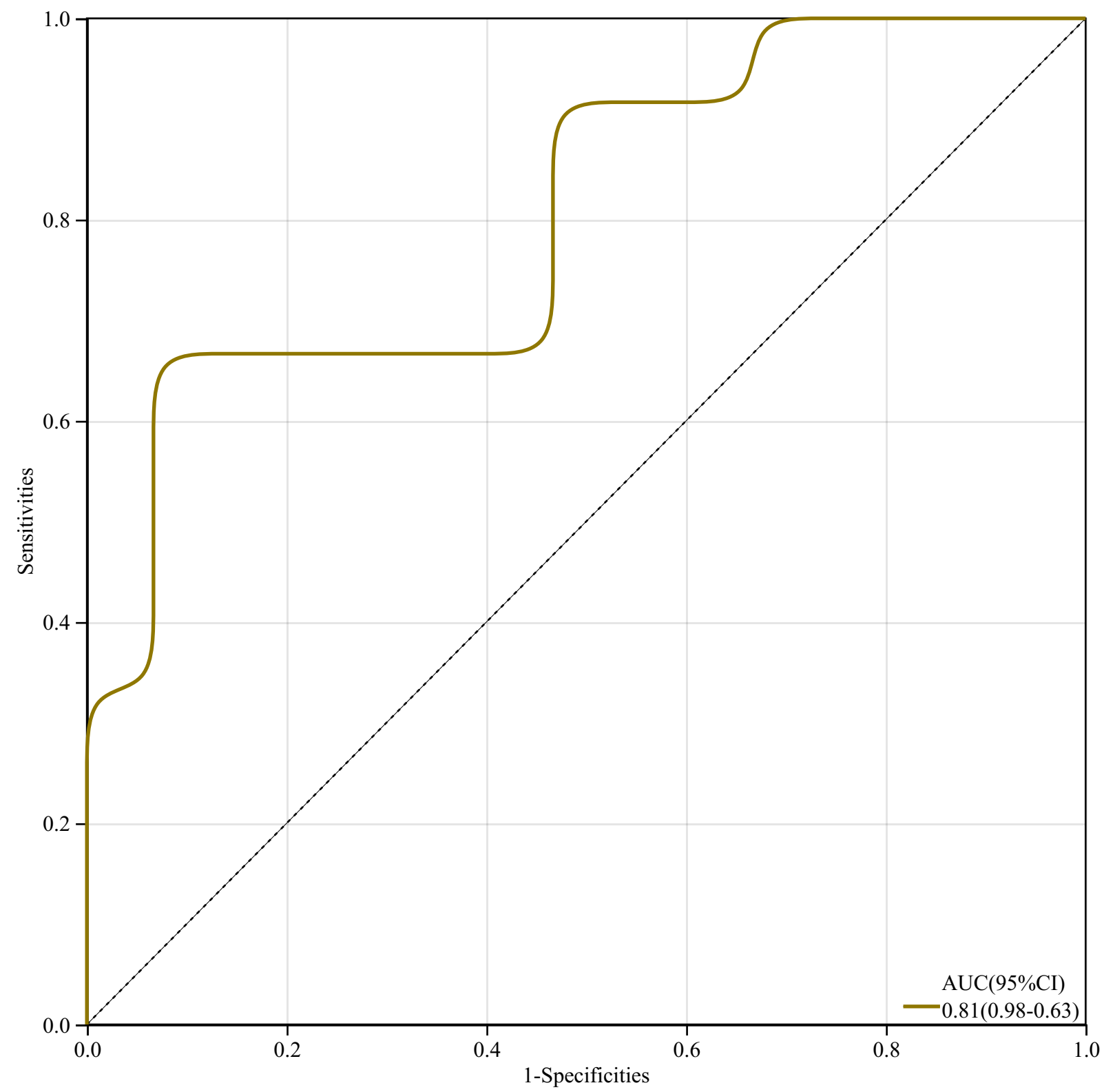

Supplement: Supplementary file 8 [file DataSheet_8.zip › Step8/1.pdf]

riskScore

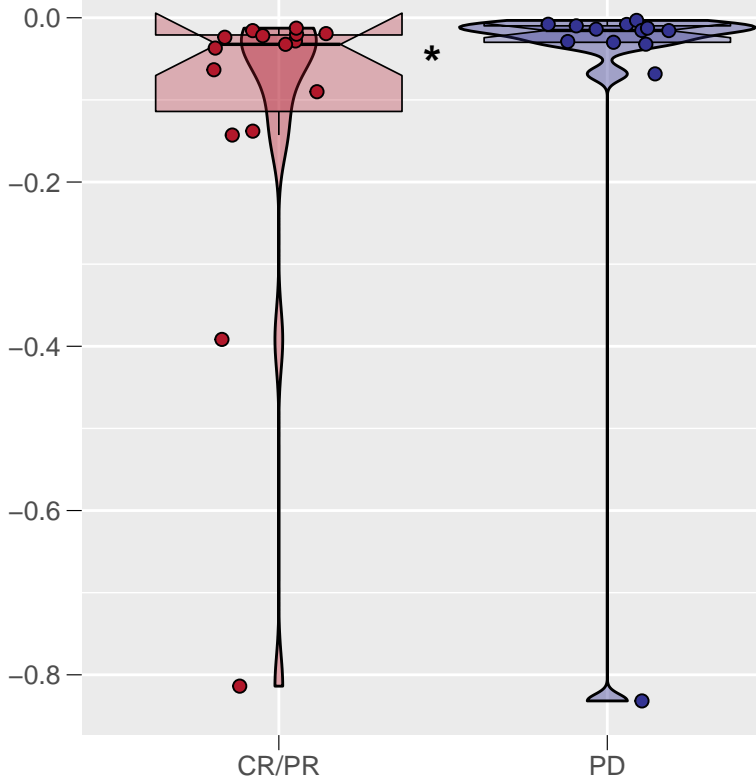

Supplement: Supplementary file 8 [file DataSheet_8.zip › Step8/GSE78220/1.pdf]

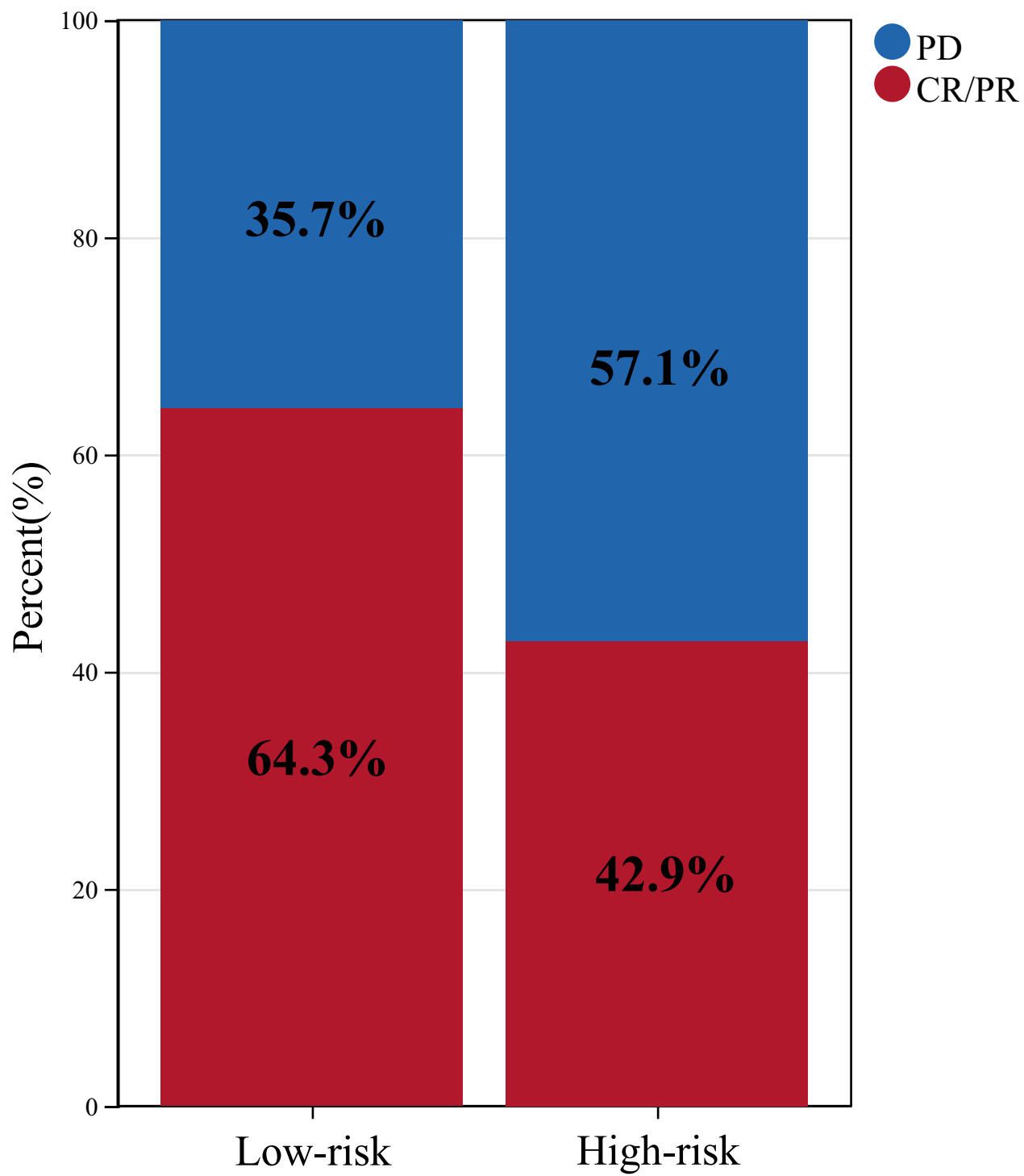

Supplement: Supplementary file 8 [file DataSheet_8.zip › Step8/GSE78220/2.pdf]

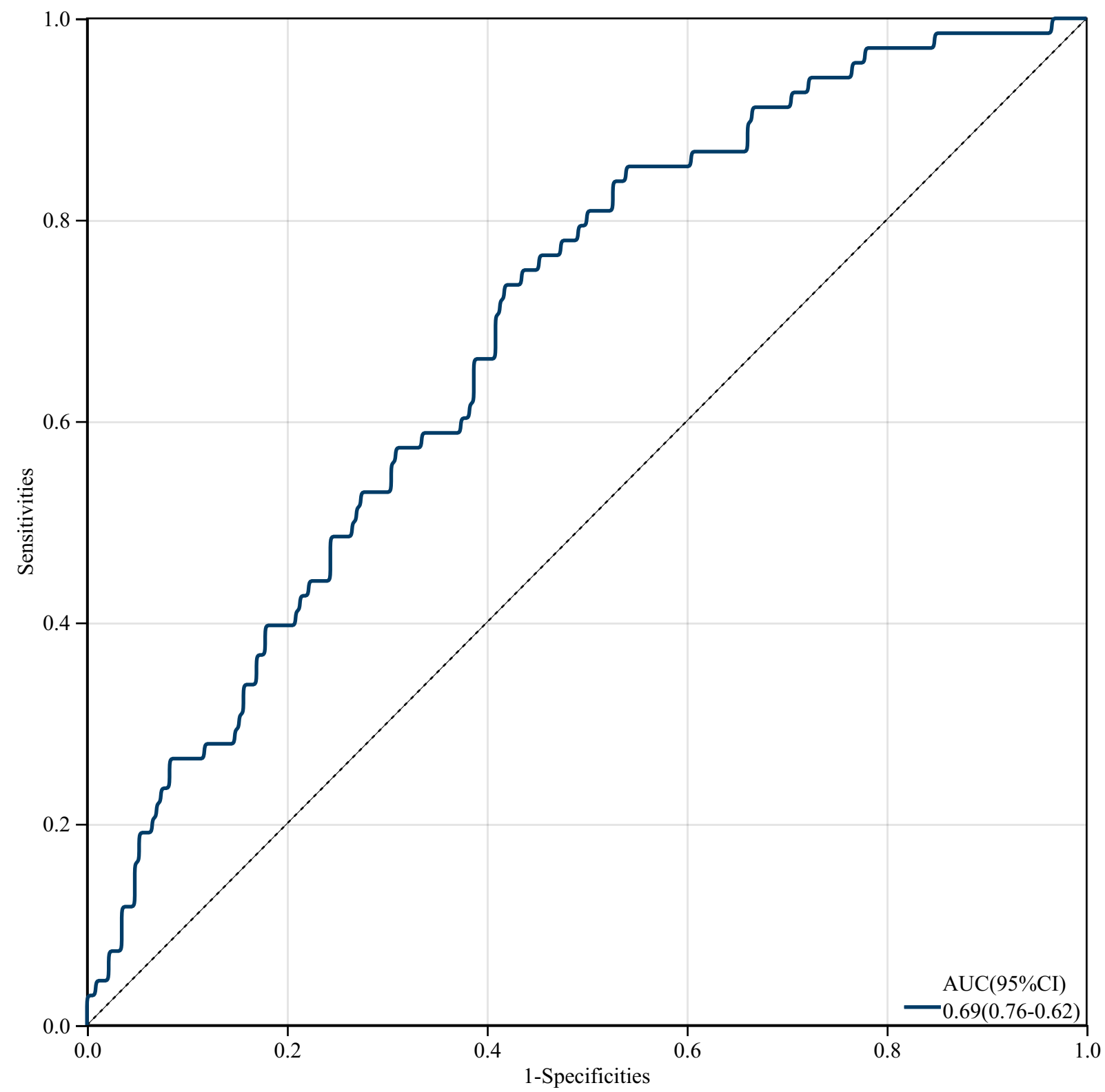

Supplement: Supplementary file 8 [file DataSheet_8.zip › Step8/im.pdf]

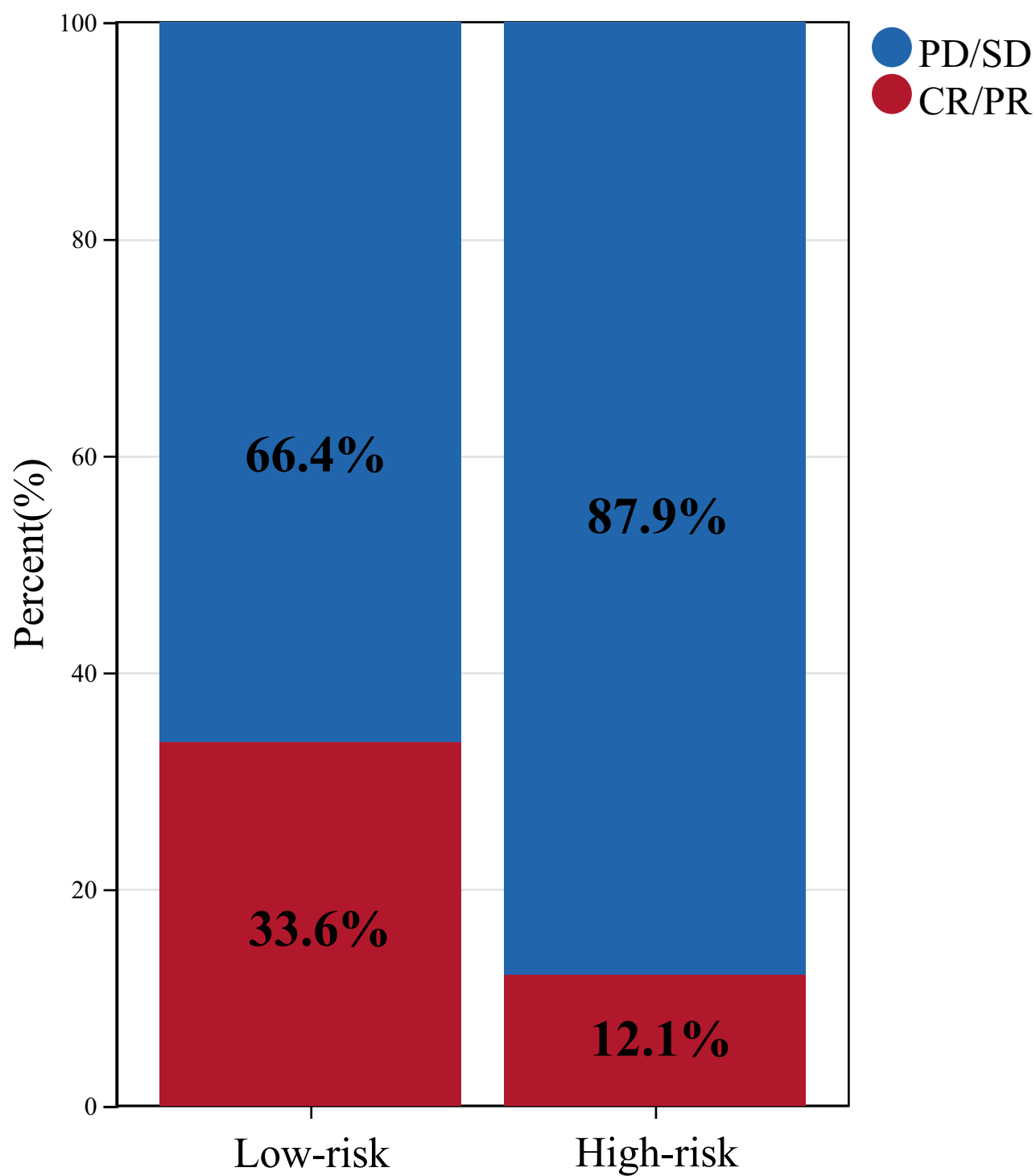

Supplement: Supplementary file 8 [file DataSheet_8.zip › Step8/iMvigor/imv.pdf]

riskScore

\*\*\*

CR/PR

PD/SD

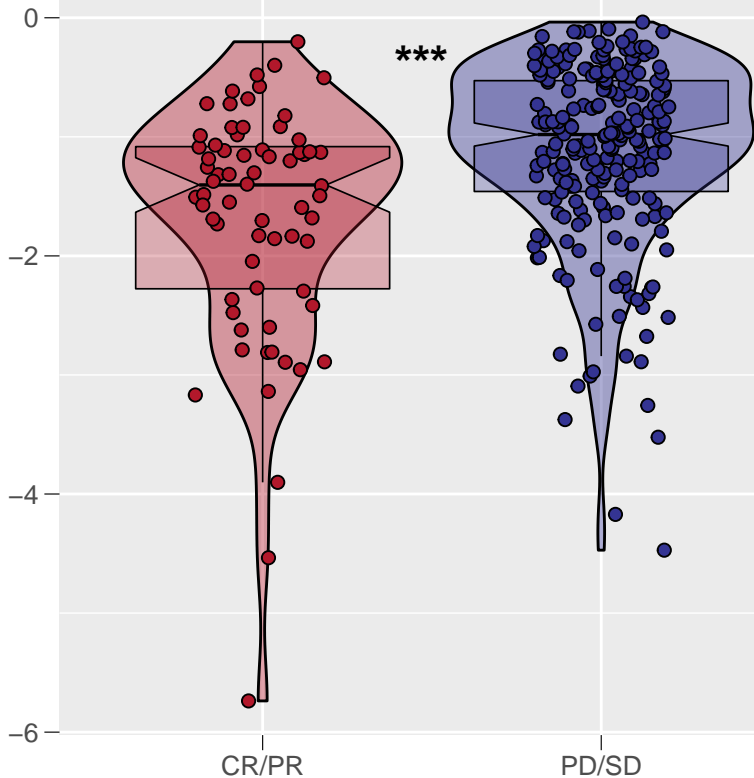

Supplement: Supplementary file 8 [file DataSheet_8.zip › Step8/iMvigor/IMV1.pdf]

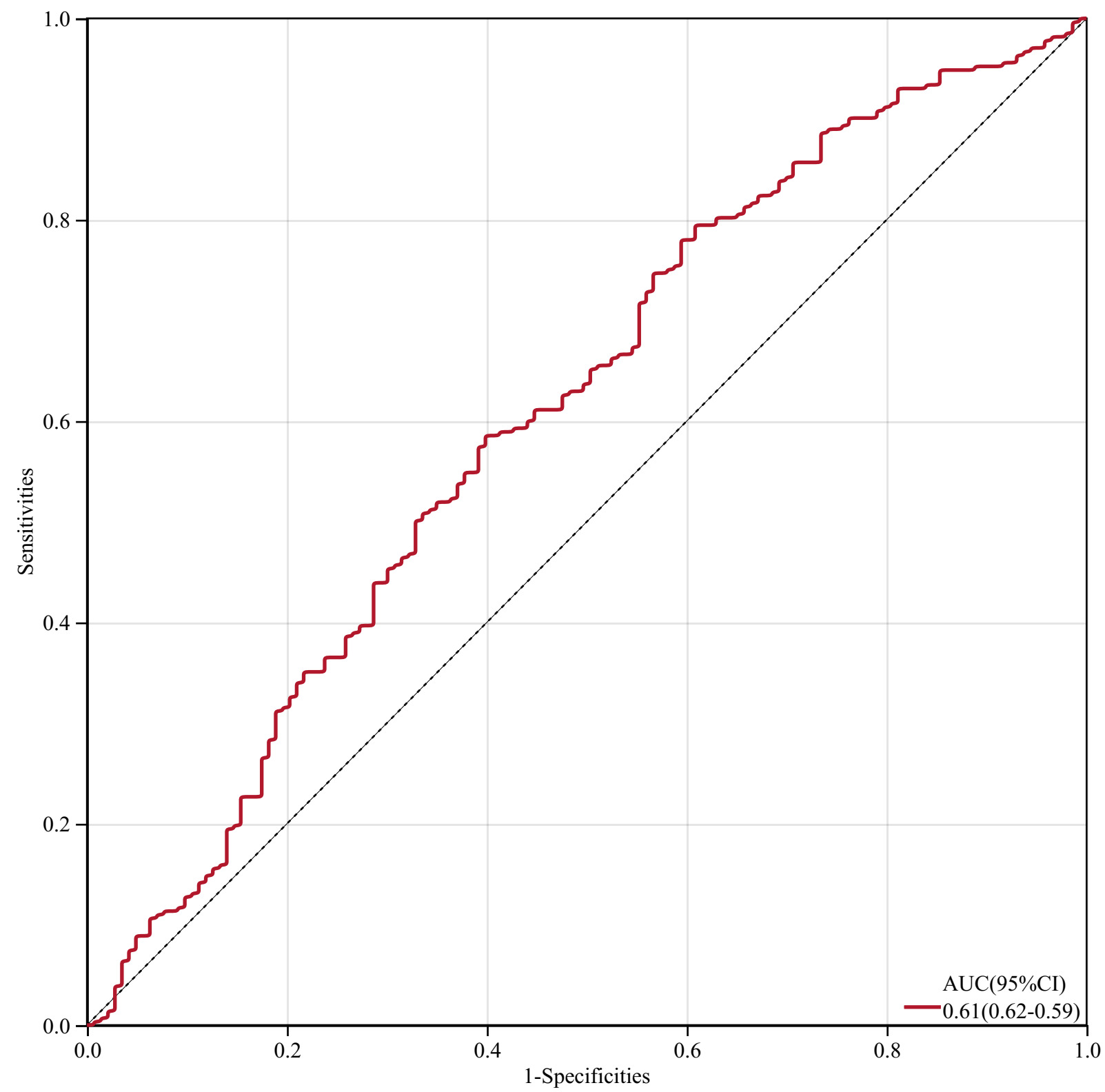

Supplement: Supplementary file 8 [file DataSheet_8.zip › Step8/tide.pdf]

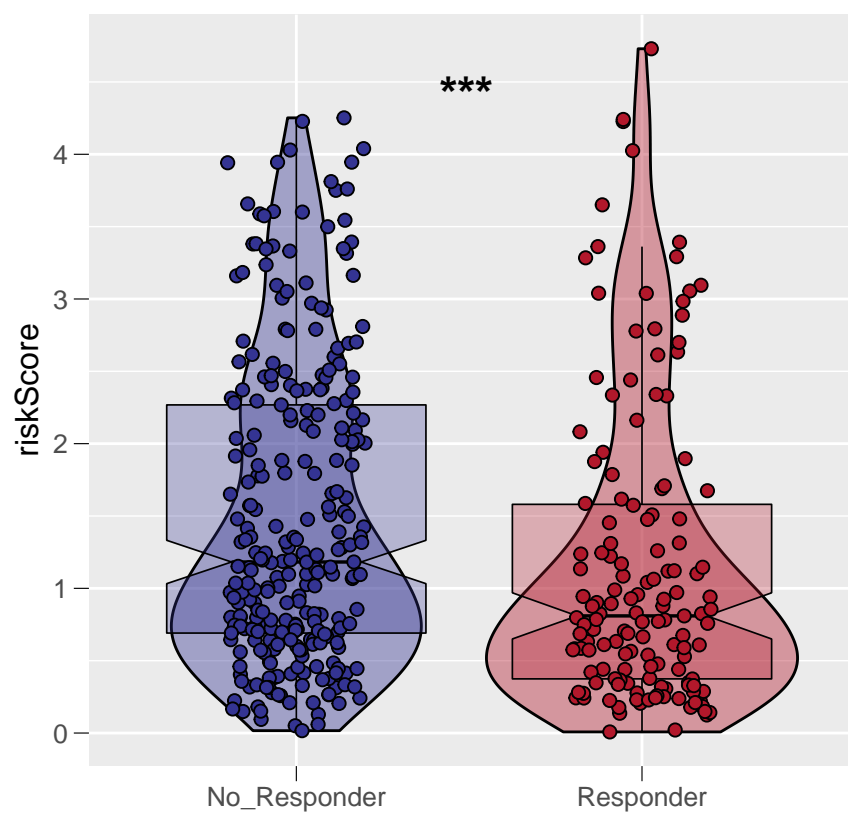

Supplement: Supplementary file 8 [file DataSheet_8.zip › Step8/TIDE/2.pdf]

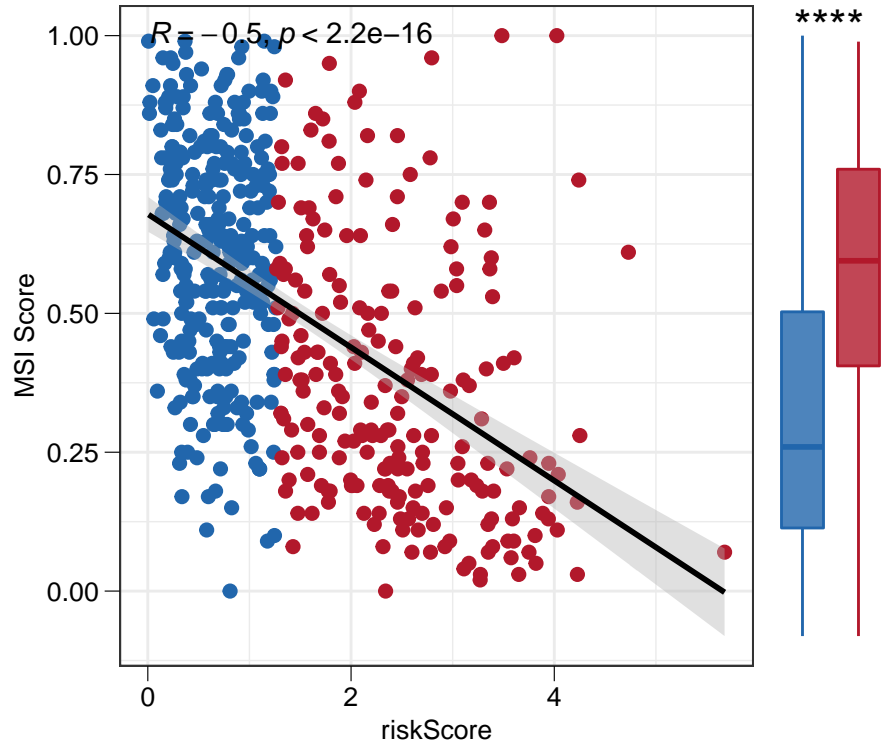

Supplement: Supplementary file 8 [file DataSheet_8.zip › Step8/TIDE/MSI.pdf]

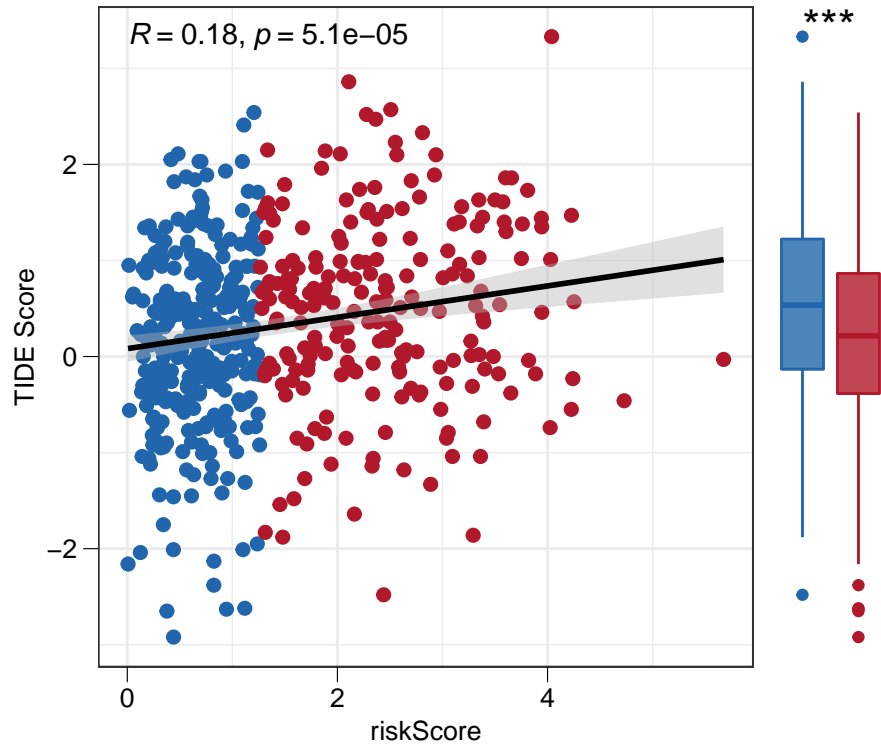

Supplement: Supplementary file 8 [file DataSheet_8.zip › Step8/TIDE/TIDE.pdf]

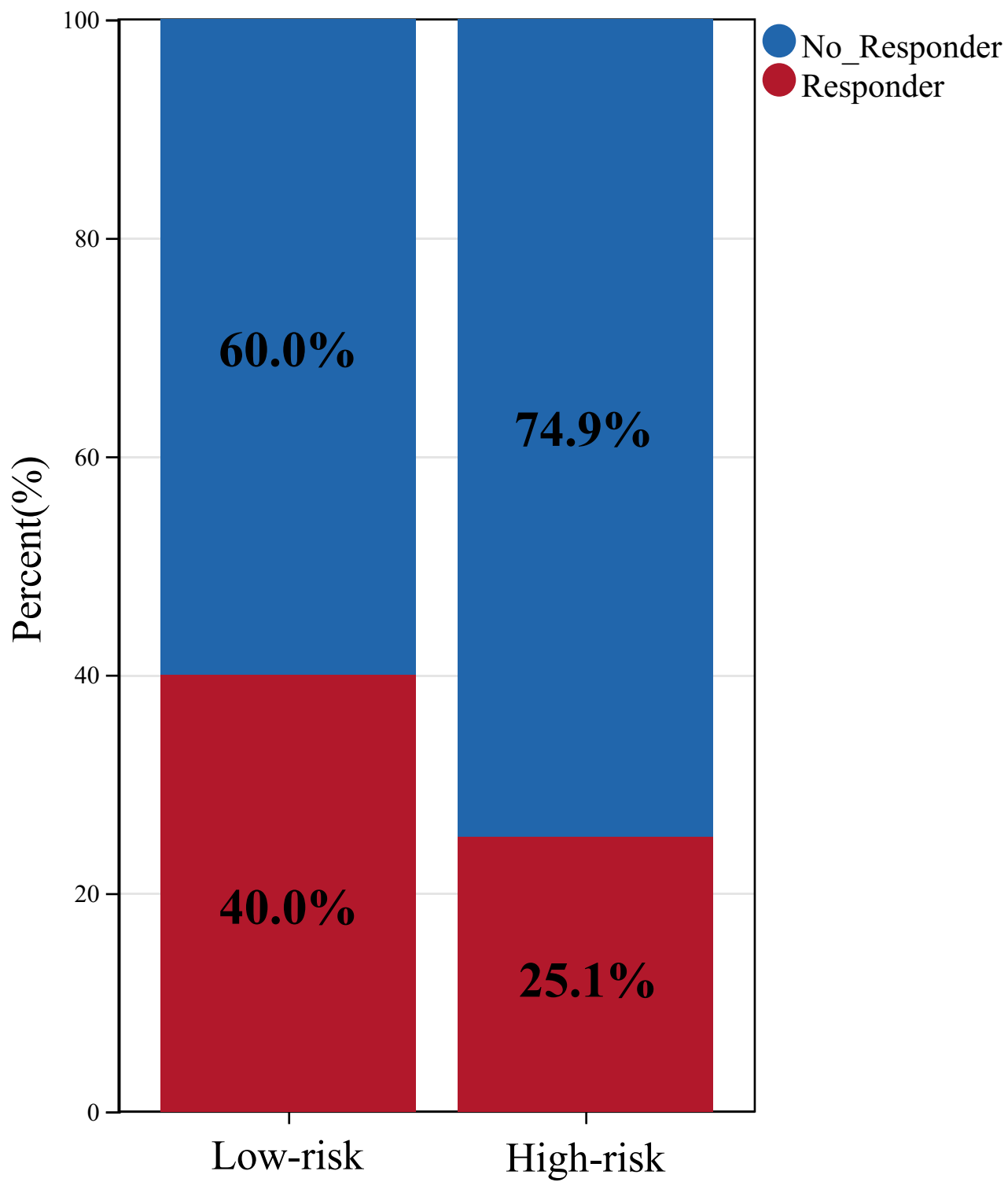

Supplement: Supplementary file 8 [file DataSheet_8.zip › Step8/TIDE/zhu.pdf]
